# Consecutive non-natural PZ nucleobase pairs in DNA impact helical structure as seen in 50 μs molecular dynamics simulations

**DOI:** 10.1093/nar/gkx144

**Published:** 2017-02-28

**Authors:** Robert W. Molt, Millie M. Georgiadis, Nigel G.J. Richards

**Affiliations:** 1 Department of Biochemistry and Molecular Biology, Indiana University School of Medicine, Indianapolis, IN 46202, USA; 2 School of Chemistry, Cardiff University, Cardiff, CF10 3AT, UK; 3 Department of Chemistry and Chemical Biology, Indiana University Purdue University Indianapolis, Indianapolis, IN 46202, USA; 4 ENSCO, Inc., 4849 North Wickham Road, Melbourne, FL 32940, USA

## Abstract

Little is known about the influence of multiple consecutive ‘non-standard’ (**Z**, 6-amino-5-nitro-2(1H)-pyridone, and **P**, 2-amino-imidazo[1,2-a]-1,3,5-triazin-4(8H)-one) nucleobase pairs on the structural parameters of duplex DNA. **P:Z** nucleobase pairs follow standard rules for Watson–Crick base pairing but have rearranged hydrogen bonding donor and acceptor groups. Using the X-ray crystal structure as a starting point, we have modeled the motions of a DNA duplex built from a self-complementary oligonucleotide (5΄-CTTATPPPZZZATAAG-3΄) in water over a period of 50 μs and calculated DNA local parameters, step parameters, helix parameters, and major/minor groove widths to examine how the presence of multiple, consecutive **P:Z** nucleobase pairs might impact helical structure. In these simulations, the **PZ**-containing DNA duplex exhibits a significantly wider major groove and greater average values of stagger, slide, rise, twist and h-rise than observed for a ‘control’ oligonucleotide in which **P:Z** nucleobase pairs are replaced by **G:C**. The molecular origins of these structural changes are likely associated with at least two differences between **P:Z** and **G:C**. First, the electrostatic properties of **P:Z** differ from **G:C** in terms of density distribution and dipole moment. Second, differences are seen in the base stacking of **P:Z** pairs in dinucleotide steps, arising from energetically favorable stacking of the nitro group in **Z** with π–electrons of the adjacent base.

## INTRODUCTION

**Figure 1.**
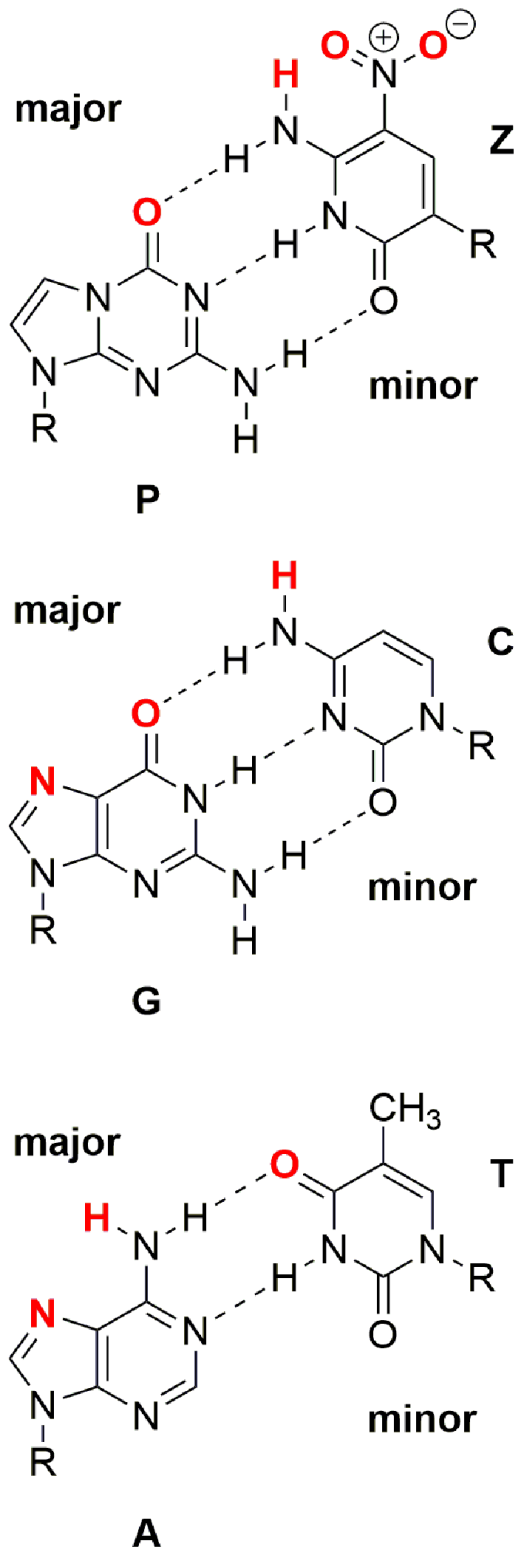
Chemical structures of **P**:**Z, G**:**C**, and **A**:**T** nucleobase pairs. R is 2΄-deoxyribose in duplex DNA. Atoms shown in red can participate in intermolecular hydrogen bonding interactions in the major groove.

The design and synthesis of artificially expanded genetic information systems (AEGIS) capable of Darwinian evolution is a theme of growing importance in synthetic biology (1–5). A number of novel nucleobase pairs that meet the size and/or complementarity rules of Watson–Crick base pairing have been described over the past two decades (6–9), and the successful replication of one of these ‘non-standard’ nucleobase pairs in *Escherichia coli* has been reported (10). More specifically, the Benner laboratory recognized that the four distinct ‘standard’ building blocks (**A, G, C, T** and its equivalent **U**) in DNA and RNA do not exhaust the constraints imposed by rules guiding Watson–Crick pairing in natural nucleic acids. As a result, the number of nucleobase pairs can be increased from two to six merely by rearranging hydrogen bond donor and acceptor groups (11). An essential property of duplex DNA containing AEGIS nucleobase pairs is that it must be efficiently replicated by DNA polymerases (12,13). Thus, the DNA template–primer substrate must adopt an A-form helix within the active site of the polymerase rather than B-form (14), even though the preferred form of duplex DNA in solution is B-form (15). Duplex DNA containing AEGIS nucleobases must therefore retain the ability to interconvert between helical forms. In this regard, DNA molecules containing the **P:Z** nucleobase pair (Figure 1) are of especial interest given that polymerase variants have been obtained that can replicate this nucleobase pair (16,17). The **Z** nucleobase differs from a standard pyrmidine nucleobase in that it includes a nitro group and a C-glycosidic linkage to the sugar rather than the standard N-glycosidic linkage. This nitro group, which is unique to **Z**, provides functionality in the major groove not present in standard nucleobases. The purine nucleobase **P**, which is complementary to **Z**, is similar to G differing only in the donor acceptor patterns. Moreover, our group has reported crystal structures of duplex DNA containing multiple copies of the **P:Z** nucleobase pair (18). These studies have shown that B-form DNA tolerates the inclusion of two consecutive **P:Z** nucleobase pairs with minimal structural impact on the double helix when compared to B-form DNA containing only **A**:**T** or **G:C** base pairs (18).

We note that duplex DNA sequences comprised of several consecutive purines (**A** or **G**) have unique structural properties with underlying biological function, and, in a similar manner, the properties of duplex DNA containing greater numbers of consecutive **P:Z** nucleobase pairs are of considerable interest. For example, a self-complementary 16-bp oligonucleotide containing six consecutive **P:Z** nucleobase pairs (5΄-CTTAT**PPPZZZ**ATAAG-3΄) crystallizes as an A-form duplex (18), exhibiting a novel stacking interaction between the nitro group of **Z** and the rings of adjacent **Z** or **P** nucleobase. On the other hand, this same 16-bp oligonucleotide under low salt conditions gives a CD spectrum corresponding to B-form, that is similar to that of an oligonucleotide (5΄-CTTATGGGCCCATAAG-3΄) in which **P:Z** nucleobase pairs are replaced by **G:C**. A previous quantum mechanical study has addressed the stability of hydrogen-bonding in the **Z:P** pair finding it comparable to G:C (19). This same study compared base stacking of the Z-nitro group with another Z or P nucleobase as compared to Z with the nitro group replaced by an H atom and found the ZP/ZP or ZZ/PP stacking interactions to be more favorable (19).

Of course, X-ray crystallography provides an averaged static description of the conformational properties of duplex DNA oligonucleotides. A prior molecular dynamics (MD) study, however, concluded that helix dynamics are not significantly affected by inclusion of a *single***Z:P** nucleobase pair within a double helical DNA molecule (20). This conclusion is encouraging because disruption of the structure of DNA by a single **Z:P** nucleobase pair would not bode well for expansion of the genome. On the other hand, nothing is known about the effects of incorporating *multiple***Z:P** nucleobase pairs into a DNA duplex. It is known that *multiple, consecutive* base-pair sequences can result in a cumulative effect on overall helix properties (21,22), and so we sought to understand how the 16-bp DNA duplex containing six consecutive **P:Z** nucleobase pairs might undergo conformational transitions in water. Here, we report long timescale (50 μs) MD simulations using an explicit solvent model to understand the nature of the solution-phase dynamic structure. Similar calculations have previously provided considerable insight into the conformational preferences and dynamic properties of ‘natural’ DNA (23–28). Our results suggest that the presence of six consecutive **P:Z** nucleobase pairs changes the structural properties when compared to a ‘control’ oligonucleotide containing only standard base pairs. The molecular origins of these changes are likely associated with differences in the electrostatic properties and stacking interactions of **P:Z** and **G:C** nucleobase pairs.

## MATERIALS AND METHODS

Initial structures were prepared using the AMBER 2015 software suite (29) from the crystal structure of the self-complementary oligonucleotide (5΄-C**B**TAT**PPPZZZ**ATAAG-3΄) (PDB ID: 4XNO) after the bromine atom within the 5-bromo-uridine (**B**), present in the crystal structure for phasing purposes, was replaced with a methyl group. Similarly, the resulting **PZ**-containing duplex was used to generate coordinates for the control oligonucleotide structure by replacing **P** with **G** and **Z** with **C** nucleobases. Each DNA duplex was placed in a 10 Å height truncated octahedral box, and 30 sodium ions were added to neutralize the overall charge of the system. Fifteen extra Na^+^ and Cl^−^ ions were added to result in the chosen ionic strength of 149 mM (as close to 150 mM as we could approximate).

We utilized the ff99bsc0 force field (30) to describe all atoms of each oligonucleotide, except for the **P** and **Z** nucleobases, which were assigned the parameter values from the ff99bsc0 force field where possible, with values for missing parameter types being obtained from the generalized AMBER force field (GAFF) force field (Supplementary Data) (31). The rest of the system was modeled with the TIP3P water model (32) in conjunction with the ion force field of Joung/Cheatham (33,34). We note that the default GAFF improper dihedral parameter for the fused-ring nitrogens in the **P** nucleobase must be changed to 5.6 kcal/mol in order to maintain ring planarity.

Each system was energy minimized to remove bad contacts (4000 steepest descent followed by 4000 conjugate gradient steps), heated (0–300 K over a period of 10 ps in the NVT ensemble), and then equilibrated for 100 ns (to allow the quasi-immobile ions sufficient time to equilibrate as reported previously (25)) prior to the production phase of the trajectory, from which snapshots were taken at intervals of 2 ps. The SHAKE algorithm (35) was used so that 2 fs time steps were possible in the MD simulations. Langevin dynamics were used to regulate the temperature, with a collision frequency of 1 ps^−1^. Periodic boundary conditions were used in all MD simulations, with an 8.5 Å cutoff being used for non-bonded interactions, and particle-mesh Ewald methods were used to describe long-range electrostatics (36–38). Equilibration/production simulations were run in the NPT ensemble using a Monte Carlo barostat (39). This allowed proper equilibration of the density as well as rapid execution of dynamics without a need for switching ensembles. The total simulation time for both 16-bp oligonucleotides was 50 μs, and trajectories were generated using the GPU implementation of the MD algorithms (40). The CPPTRAJ (41) and VMD (42) software packages were used to convert trajectory snapshots into GROMACS format for statistical analysis using the 3DNA (43) and do_x3DNA programs (44).

Dipole moments for both nucleobase pairs were calculated via CCSD (45–48)/aug’-cc-pVDZ (49,50) using the ACES4 (51) software package at M06-2X (52)/aug’-cc-pVDZ optimized geometries. The prime denotes no diffuse functions were used on hydrogen atoms (53). Spherical *d* functions were used throughout, and core basis functions were omitted. All physical constants came from the 2014 CODATA standards (54). All reference determinants were converged to 10^−6^ change in density matrix elements. Stationary states were confirmed to be minima using harmonic normal mode analysis. Kohn-Sham Density Functional Theory (KS-DFT) (55,56) calculations used a Lebedev grid consisting of 99 radial and 590 solid angle points, as implemented in Gaussian09 (57). Converged geometries were defined as having a maximum Root Mean Square (RMS) force on any geometric parameter and a total RMS force of no greater than 3.3 × 10^−4^ and 1.0 × 10^−4^ Hartree/Bohr, respectively. Electrostatic potential maps were constructed from the KS-DFT density matrix and visualized using the Gaussview software (58). The isocontour value of density was 0.001 elementary charge per cubic Bohr.

Qualitative differences in van der Waals energetics for stacked gas-phase **PZ**-containing and GC-containing Watson–Crick dinucleotides were obtained from single point energy calculations using M06-2X functional, with the ribose ring being represented by a methyl group substituent. We performed two sets of KS-DFT calculations for dinucleotide steps: an energy scan, in which we varied the stacking distance between the Watson–Crick pairs without re-optimization, and a global optimization of all degrees of freedom for the minimum dinucleotide step structures. The purpose of the former was to gauge the qualitative differences in van der Waals energetics of stacking. The latter corresponded to a comparison of optimized dinucleotides and thus which conformations of **PZ** were more favorable energetically (and thus required optimization).

Structures for the vertically stacked dinucleotide step energy scan were obtained by placing individual M06-2x/aug’-cc-pVDZ optimized nucleobase pairs in identical, vertically shifted orientations. Subsequent structures were generated by changing the vertical separation of the nucleobase pairs. We did not globally re-optimize these new structures; any quantitative description would require the use of computationally prohibitive van der Waals descriptions (59,60). We calculated the stacking interaction energy in both the gas phase (with basis-set superposition error accounted for via standard counter-poise correction) as well as with implicit solvent via the Solvation Model based on Density (SMD) (61) self-consistent reaction field model. We used the aug’-cc-pVDZ basis with the implicit solvent calculations, as it was also used for the optimizations. For the counter-poise corrections, diffuse functions on carbons were omitted due to difficulty in converging the ghost atom Self Consistent Field (SCFs) (likely due to linear dependencies of the rings closely interacting).

The optimized stacked **PP/ZZ** and GG/CC structures followed the above procedures associated with KS-DFT calculations in terms of functional, basis set, integration grid, SCF convergence criteria, geometry optimization and software used. In particular, the conformations were generated starting from the purely vertically stacked dimers and ‘pulling’ one Watson–Crick pair along either the slide axis or shift axis. The result of many attempted optimizations of different stacked-ring conformations for **PP/ZZ** were two conformers, one labeled as the ‘slide’ conformer and one as the ‘shift’ conformer for reasons which will become evident. All calculations were counterpoise-corrected.

Estimates of Watson–Crick hydrogen bonding energy employed CCSD(T) (62–64)/aug’’-cc-pVTZ, calculations on structures optimized at the M06-2X/aug’-cc-pVDZ, with the ribose ring being represented by a methyl substituent. The double prime indicates the absence of diffuse functions on hydrogen and carbon. Hydrogen bonding energy was defined as the difference in energy between the sum of individually optimized nucleobase energies and the energy of the optimized Watson–Crick pair. Free energies were calculated via partition functions (65–67); analytic partition functions are known for translational contributions via quantum particle-in-a-box, quantum rigid rotor and quantum harmonic oscillator. The conformational partition function is 1 for the hydrogen-bonded Watson–Crick structure (there are no other conformations possible) (68). The requisite chemical parameters in the above stated partition functions (force constants, moments of inertia, etc.) are standard outputs of any calculation of harmonic vibrational modes (69).

## RESULTS AND DISCUSSION

### Time convergence and statistics of structural parameters in the MD simulations

Long time-scale MD simulations (50 μs) were performed on the two 16-bp, self-complementary DNA oligonucleotides (PZ: 5΄-CTTATPPPZZZATAAG-3΄ and GC: 5΄-CTTATGGGCCCAT-AAG-3΄) in water using the AMBER software suite (29). These simulations used the AMBER ff99bsc0 force field (30), with missing parameters for the **P** and **Z** nucleobases being obtained from the GAFF (31) following standard procedures. Both initial structures were in A-form, as observed in the X-ray crystal structure of the 16-bp oligonucleotide containing six consecutive **P**:**Z** nucleobase pairs. The *natural* DNA ‘control’ oligonucleotide rapidly converted to B-form in the MD simulation, consistent with prior literature observations (70). We calculated the average values of parameters describing the helical properties of each DNA duplex from ‘snapshots’ taken along each trajectory. The convergence of average parameter values was monitored (Table 1), being much faster for the **GC**-containing ‘control’ than for the **PZ**-containing duplex. Table 2 provides a comparison of selected experimental crystal helix parameters versus calculated liquid helix parameters. Overall, there is good agreement between the crystal and simulation values for both GC and **PZ**, and the same trends are observed in the comparisons of crystal to simulation for both sequences. The major groove widths for the MD simulation derived structures are larger for both **PZ** and GC, while the helical twist values are larger for both in the crystal structures. The reproduction of trends in helix parameters between the crystal structure and the MD simulation gives greater support that the GAFF parameters chosen for **PZ** nucleobases are adequate to describe the basic chemistry in this simulation. Differences observed support the greater conformational space sampled by the GC and **PZ** sequences during the MD simulations.

**Table 1.** Time convergence of shear (Å), roll (°) and inclination (°) for the **PZ**-containing and control oligonucleotides^a^

Parameter	500 ns	5 μs	25 μs	50 μs
Shear (PZ)	0.04	−0.39	−0.21	−0.22
Shear (GC)	−0.13	−0.13	−0.13	−0.13
Roll (PZ/PZ)	2.76	3.67	3.19	3.24
Roll (GC/GC)	2.93	2.81	2.88	2.87
Inclination (PZ/PZ)	2.59	2.09	2.73	2.98
Inclination (GC/GC)	5.19	4.98	5.11	5.09

^a^The convergence of all helix parameter values is provided in Supplementary Tables S1 and S2.

**Table 2.** Comparison of experimental and computational PZ and GC Helix parameters^a^

	PZ Exp.	PZ Comp.	GC Exp.	GC Comp.
Slide (Å)	−2.3	−2.9	−0.3	−0.8
Rise (Å)	3.2	3.3	3.4	3.2
Roll (°)	1.1	3.2	−2.3	2.9
H-Twist (°)	29.4	22.1	45.0	34.3
Major Groove (Å)	20.1	26.8	15.6	21.0
Minor Groove (Å)	15.8	13.4	11.2	12.9

^a^Values are presented for the central PZ/PZ or GC/GC dinucleotide step in the experimental crystal structures and MD-derived structures.

The mean values of all local, step, helical and groove width parameters were determined from the two types of MD trajectories for the self-complementary **PZ** and GC oligonucleotide duplexes (Table 3). All of the average values for the helix parameters (25) of the ‘control’ GC oligonucleotide were in statistical agreement via t-test with the previously published Drew–Dickerson dodecamer helix values (71), aside from a small difference in slide (Table 3 and Supplementary Table S3). Calculated average parameter values for symmetry unique positions are within ± 0.1°, suggesting that there was adequate phase space sampling in the MD simulations. The mean and variation computed for the local parameters of the two DNA duplexes were similar with the exception of stagger, which is twice as large for the **PZ** than the GC helix. More interestingly, the dinucleotide step parameters for the **PZ** and GC helices show greater differences. For example, the average ‘slide’ value is three times larger for the oligonucleotide containing six consecutive **P:Z** nucleobase pairs compared with that of the control oligonucleotide (Table 3). On the other hand, the mean values for twist and h-twist are about 10° smaller on average for **PZ/PZ** than GC/GC pairs, with a greater variance (Table 3). Most noticeably, the major and minor grooves are wider in the **PZ** duplex than those in the GC duplex. Lastly, we note that the values of X-displacement and Y-displacement appear to have no physical meaning for the **PZ**-containing duplex due to the internal reference frames and definitions used in standard helix analysis. An example of such a structure is one in which the X-displacement is calculated to be 66 Å for the central dinucleotide step (see Supplementary Data). This is, however, an artifact of strict adherence to the reference frames defined in 3DNA (see Supplementary Data). Moreover, from the distributions of all the helix parameters, including X-displacement and Y-displacement (provided in Supplementary Data), one can readily see that extreme values represent only a small portion of those observed within the distribution tails. The average is still zero, consistent with other calculations in the literature, despite the extreme values of the tails (71).

**Table 3.** Helix parameters calculated from the MD trajectories of the **PZ** and **GC**-containing DNA duplexes^a^

	Shear (Å)	Stretch (Å)	Stagger (Å)	Buckle (°)	Propeller (°)	Opening (°)
PZ	−0.10 ± 0.41	−0.04 ± 0.14	−0.19 ± 0.42	−4.3 ± 9.8	−3.2 ± 8.4	−2.6 ± 4.0
GC	−0.15 ± 0.31	−0.06 ± 0.11	−0.08 ± 0.37	−0.5 ± 10.3	−6.8 ± 8.0	−0.3 ± 3.2
	**Shift (Å)**	**Slide (Å)**	**Rise (Å)**	**Tilt (°)**	**Roll (°)**	**Twist (°)**
PZ/PZ^b^	−0.04 ± 0.82	−2.86 ± 1.32	3.29 ±0.32	0.2 ± 4.6	3.2 ± 5.9	21.6 ± 15.2
GC/GC^b^	0.00 ± 0.55	−0.78 ± 0.57	3.19 ± 0.30	0.0 ± 3.9	2.9 ± 5.0	33.6 ± 4.3
	**H-Rise (Å)**	**Inclination (°)**	**Tip (°)**	**H-Twist (°)**	**Major Groove (Å)^c^**	**Minor Groove (Å)^c^**
PZ/PZ^b^	3.11 ± 1.15	3.0±16.6	0.0 ± 12.0	22.1 ± 16.4	26.8 ± 1.7	13.4 ± 1.3
GC/GC^b^	3.08 ± 0.36	5.1 ± 8.6	0.0 ± 6.8	34.3 ± 4.2	21.0 ± 2.4	12.9 ± 1.1

^a^The standard deviation reported here represents the distributional variation of the fluctuating dynamic quantity. Precision bounds are based on a 95% confidence interval with respect to the standard error of the mean (<0.08 Å and 1° for lengths and angles, respectively). See Supplementary Table S3.

^b^We have arbitrarily chosen to list only GC/GC and PZ/PZ dinucleotide steps; values for GG/CC and PP/ZZ are given in Supplementary Tables S5–S8.

^c^Major and minor groove are averages for the central PZ or GC dinucleotide steps.

**Figure 2.**
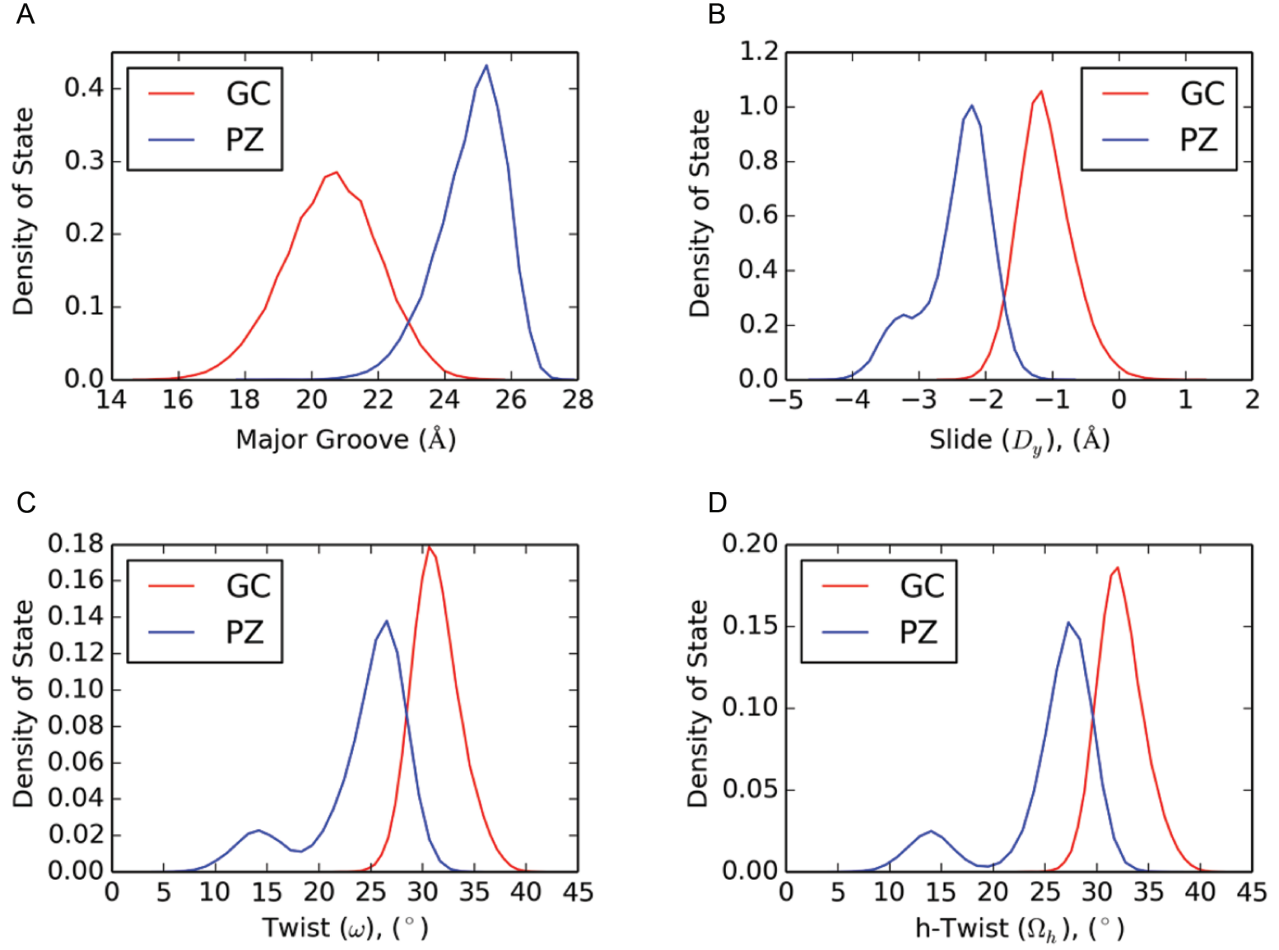
Representative histograms for selected helical parameters (**A**: major groove width; **B**: slide; **C**: twist; **D**: h-twist) for the **PZ**-containing (blue) and the control (red) oligonucleotide in the MD simulations. Plots for all other parameters are provided elsewhere (Supplementary Figure S1). The histograms show distributions over GC/GC *and* GG/CC dinucleotide steps, and over PZ/PZ *and* PP/ZZ dinucleotide steps, whereas averages are given for *only* GC/GC and PZ/PZ steps in Table 2. Density of state is defined as the number of structures for which the parameter falls in a defined range divided by the total number of structures sampled in each trajectory.

**Figure 3.**
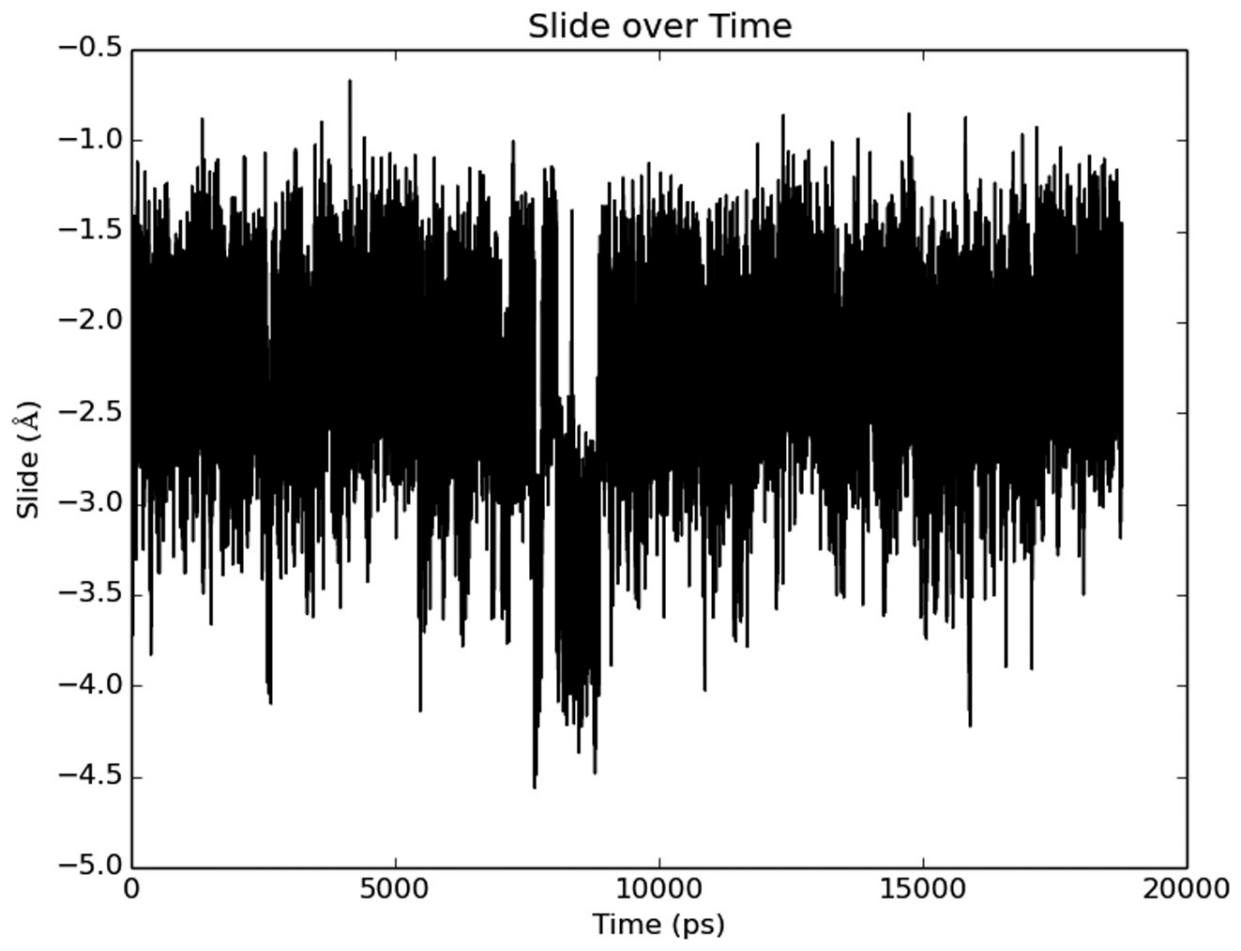
Time-dependence of slide value for the **PZ** duplex over a randomly chosen 20 ns period taken from the overall trajectory. Two densities of state are visible, one centered at ∼−2.25Å and one centered at ∼−3.5Å. All slide values for PZ-containing dinucleotide step parameters were considered for the above time evolution.

We also calculated distribution functions for selected helical parameters of structures sampled in the two types of MD trajectories (**PZ** variable helix and GC control helix) (Figure 2 and Supplementary Data). Such plots clearly show the shift in mean and standard deviation for the width of the major groove in the **PZ** (26.8 Å) and GC (21.0 Å) DNA duplexes (Figure 2A). In contrast to GC, distributions for **PZ** are bimodal for slide, twist and h-twist. The values associated with the larger peak in each case are within the range of values reported for A-form. However, the smaller peak does not correspond to values reported for A- or B-form DNA. The bimodal distributions of certain helix parameters demonstrate that at least two distinct conformers (in the free energy definition of a ‘conformer’) exist. Only a single Gaussian distribution is obtained for these parameters in the ‘control’ GC duplex (Figure 2). The variance (σ) in the data for the **PZ**-containing duplex clearly shows that a greater range of helical parameter values are sampled; the histograms also suggest that distinct conformations exist for this oligonucleotide, given that certain parameters exhibit multi-mode Gaussian distributions. A plot of slide value versus time shows the appearance of a high-density cluster within 20 ns in a randomly chosen time interval (Figure 3). The slide values show clustering of states roughly around ∼−3.5 Å and ∼−2.25 Å, in concordance with distribution of states shown in Figure 2. This observation, coupled with visual inspection of the trajectories (condensed movies of the MD simulations are provided in Supplementary Data) shows that interconversion between conformers occurs readily during the simulation. Representative structures for the PZ-containing and GC-containing oligonucleotide structures obtained from the MD simulations are provided elsewhere (Supplementary Figure S3). Global helix Root Mean Square Deviation (RMSD) plots are also available in the Supplementary Data.

**Figure 4.**
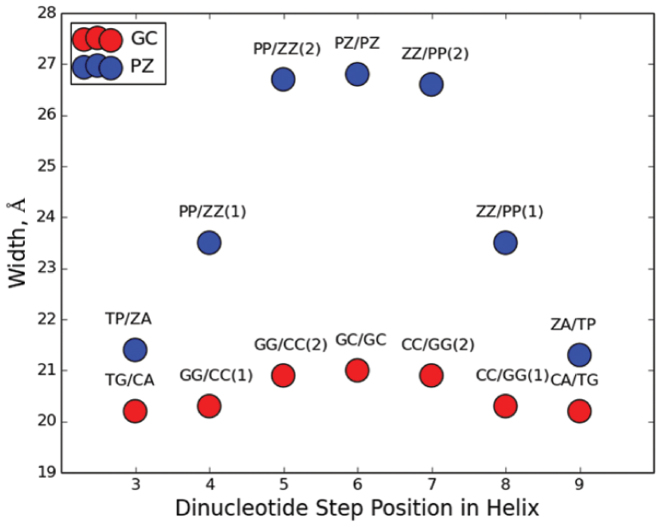
Cumulative effect of consecutive **P:Z** and **G:C** nucleobase pairs on the ‘refined’ average major groove width (39). Groove widths are shown for the central dinucleotide steps of the **PZ**- (blue) or **GC**-containing (red) oligonucleotides. The **PZ** oligonucleotide exhibits a much wider major groove centered within the region of consecutive **P:Z** nucleobase pairs than the same region of GC control oligonucleotide. The standard error of the mean for each average measured is 0.04 Å.

In addition to the variation in parameter and dinucleotide step values between **P**:**Z** and **G**:**C** nucleobase pairs (see Tables S5–S7 in Supplementary Data), the cumulative effect of having three **P:Z** pairs is evident on the major groove width of the **PZ**-containing duplex (Figure 4). In contrast, there is little cumulative effect on the major groove width seen for the **G:C** base pairs in the control duplex. One implication of this finding might be that the inclusion of large numbers of consecutive **P:Z** nucleobase pairs will yield duplex DNA that is unable to maintain a stable helical form.

### PZ-containing DNA has properties associated with both B- and A-form DNA

Common indicators of helical form (43) include scatter plots of Zp as a function of Zp(h), inclination as a function of X-displacement, roll as a function of slide, the magnitude of Zp alone, combinations of sugar dihedral angles (72–74), Zp and sugar dihedral angles (72) and the widths of the major and minor grooves (43). B-DNA possesses distinct grooves: a wide major groove and narrow minor groove. Over the course of the simulation, the GC-containing duplex also has distinctly different major and minor groove widths of 21 Å and 13 Å, respectively, which correspond well with the groove widths of this same oligonucleotide observed in the crystal structure of 18 Å and 13 Å, respectively (18). It is known, however, that ‘natural’ DNA (composed of only A, G, T and C) in MD simulations rapidly assumes B form in solution, regardless of the starting conditions and remains in this form (70,75). **PZ** features a very wide major groove (27 Å) and much narrower minor groove (13 Å) on average through the course of the simulation. In contrast, the crystal structure of the same **PZ** oligonucleotide in A-form has major and minor groove widths of 19 Å and 16.5 Å, respectively (18). In this sense, PZ DNA is more B-like, given the presence of distinctly different groove widths, albeit far wider.

In DNA crystal structures, plots of selected parameters, such as roll versus slide and Zp versus Zp(h), segregate into distinct populations associated with B- or A-forms (43). To explore in more detail how the helical preferences differ in the two oligonucleotides, we used 2000 structures from MD simulations to generate contour, rather than scatter, plots of these correlations given that conformational diversity within the solution phase for the oligonucleotides is greater than in crystal lattices. Although qualitative in nature, these plots give important insights into general trends for the helical preferences of the two oligonucleotides.

**Figure 5.**
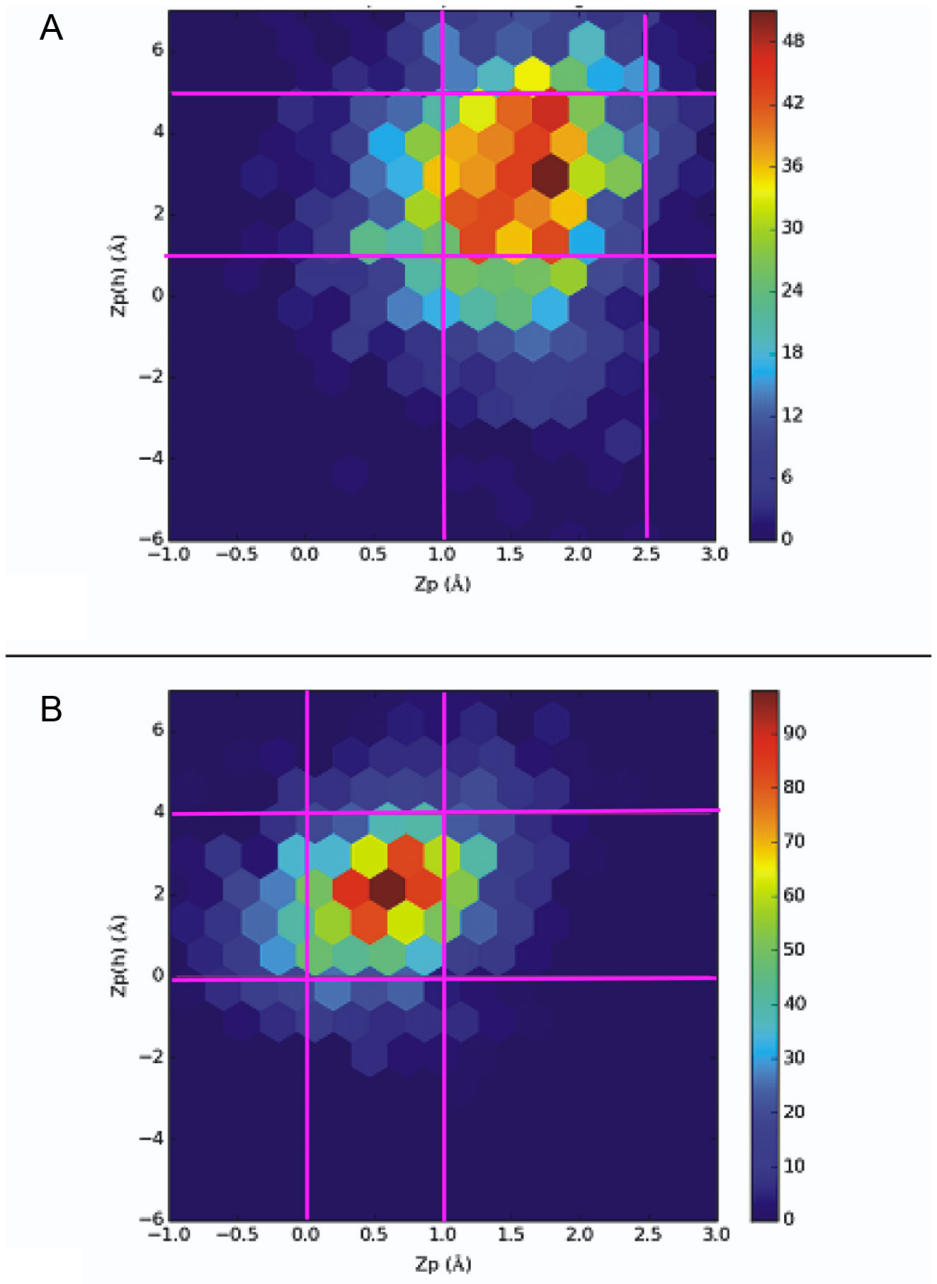
Contour plots of Zp versus Zp(h) for the (**A**) **PZ**- and (**B**) **GC**-containing DNA duplexes. Values plotted are for PZ/PZ dinucleotide steps. The pink gridlines demarcate ranges for high densities of state. Differences in scale reflect the tighter sampling of conformational forms for the GC control compared to the **PZ**-containing oligonucleotide.

In Zp(h) versus Zp scatter plots for natural DNA, A-form structures group together to show a high density of *crystalline* states greater than 1.5 Å in Zp, within a height range for Zp(h) of 2.0–6.0 Å (43). This density of states is well separated from crystalline B-like states, which have Zp values less than 0.5 Å and Zp(h) values between −2.0 and 4.0 Å. If a dinucleotide step has a Zp value of greater than 1.5 Å, it is A-like on the basis of definitions implemented in the 3DNA analysis software package (43). The contour plots of Zp versus Zp(h) show that the solution phase **PZ**-containing duplex exhibits a wider distribution of populated states when compared to the control (GC) oligonucleotide in the two MD simulations (Figure 5). In particular, the control duplex adopts a high number of structures in which the ranges of Zp and Zp(h) values are 0.0–1.0 Å and 0.0–4.0 Å, respectively (Figure 5B); similar to the values seen for B-form DNA in the crystalline state. By contrast, the density of states for the **PZ**-containing duplex is more diffuse than for GC and is concentrated in the region bounded by Zp and Zp(h) values in the range of 1.0–2.5 Å and 0.5–4.5 Å, respectively. Based on this metric, and in contrast to conclusions based on average groove width (see above), the **PZ**-containing helix is more A-like in nature. An analysis of dinucleotide steps in the 2000 ‘snapshots’ for each oligonucleotide supports this finding. We found that 53% of the **PZ/PZ** dinucleotide steps are classified as A-like and 46% are unclassified, whereas 0% of GC/GC steps are classified as A-like, with 32% being classified as B-like and 65% being unclassified.

**Figure 6.**
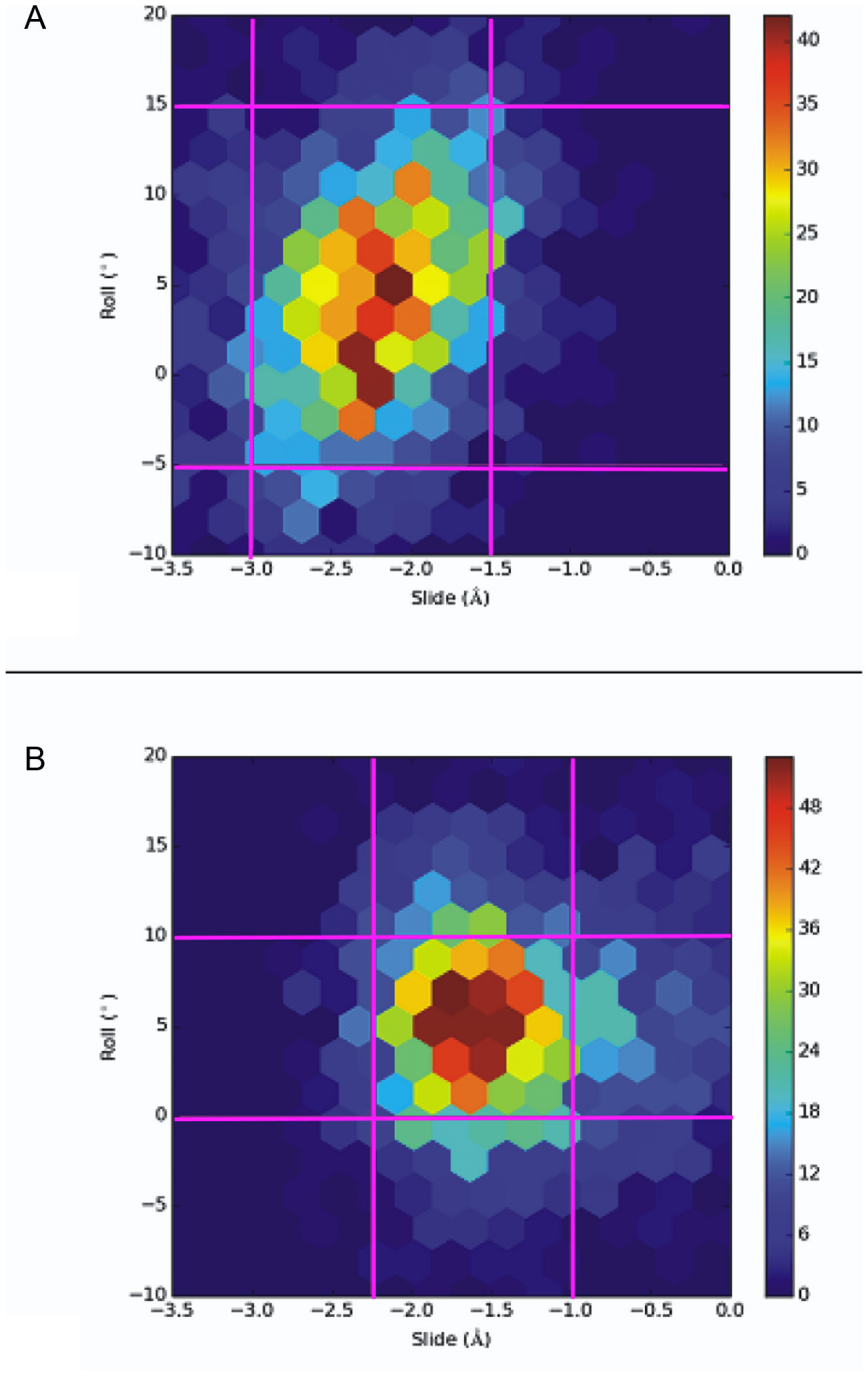
Contour plots of slide versus roll for the (**A**) **PZ**- and (**B**) **GC**-containing DNA duplexes. Values plotted are for PZ/PZ dinucleotide steps. The pink gridlines demarcate ranges for high densities of state.

For the roll versus slide metric, which segregates A-form and B-form in crystal structures, both oligonucleotides exhibit densities of state that occupy roughly the same range of values (Figure 6). Given that the control duplex is B-like in solution during the course of the MD simulation, then the correlation of roll versus slide suggests that the **PZ**-containing oligonucleotide is also B-like in solution. In addition, B-form DNA containing only Watson–Crick base pairs usually has an inclination of 0–5° whereas the value for A-form DNA is more commonly in the range of 15–20° (75); again, based on the average inclination value of 3°, the **PZ**-containing oligonucleotide is closer in structure to B-form DNA. The rise of ‘natural’ A-form and B-form DNA is 2.6 and 3.4 Å, respectively; the average rise value of 3.3 Å therefore again makes the average form of the **PZ**-containing oligonucleotide closer to B-DNA. Thus, although the **PZ**-containing oligonucleotide has a propensity to adopt a more A-like helical form as assessed by the correlation of Zp and Zp(h) values, it clearly exhibits unusual properties in that other metrics suggest it resembles B-form DNA. This complexity is only apparent upon consideration of the effect of multiple, consecutive PZ sequences. In contrast, inclusion of a single **P:Z** pair within a natural DNA context minimally perturbs the overall properties of the molecule and maintains B-form (20).

### Differences in the helical forms of the PZ- and GC-containing oligonucleotides are linked to the molecular properties of P:Z and G:C nucleobase pairs

In light of the unexpected conformational behavior observed for the **PZ**-containing oligonucleotide in water during the MD simulations, we sought to find a correlation between the AEGIS nucleobase molecular properties and the global helical structure. We examined the variation in electron density and hydrogen bonding energies between **P:Z** and **G:C** nucleobase pairs as Watson–Crick pairs and the differences in van der Waals energies for stacked bases. We emphasize that the correlation between nucleobase pair properties and helical properties is not necessarily causal.

**Figure 7.**
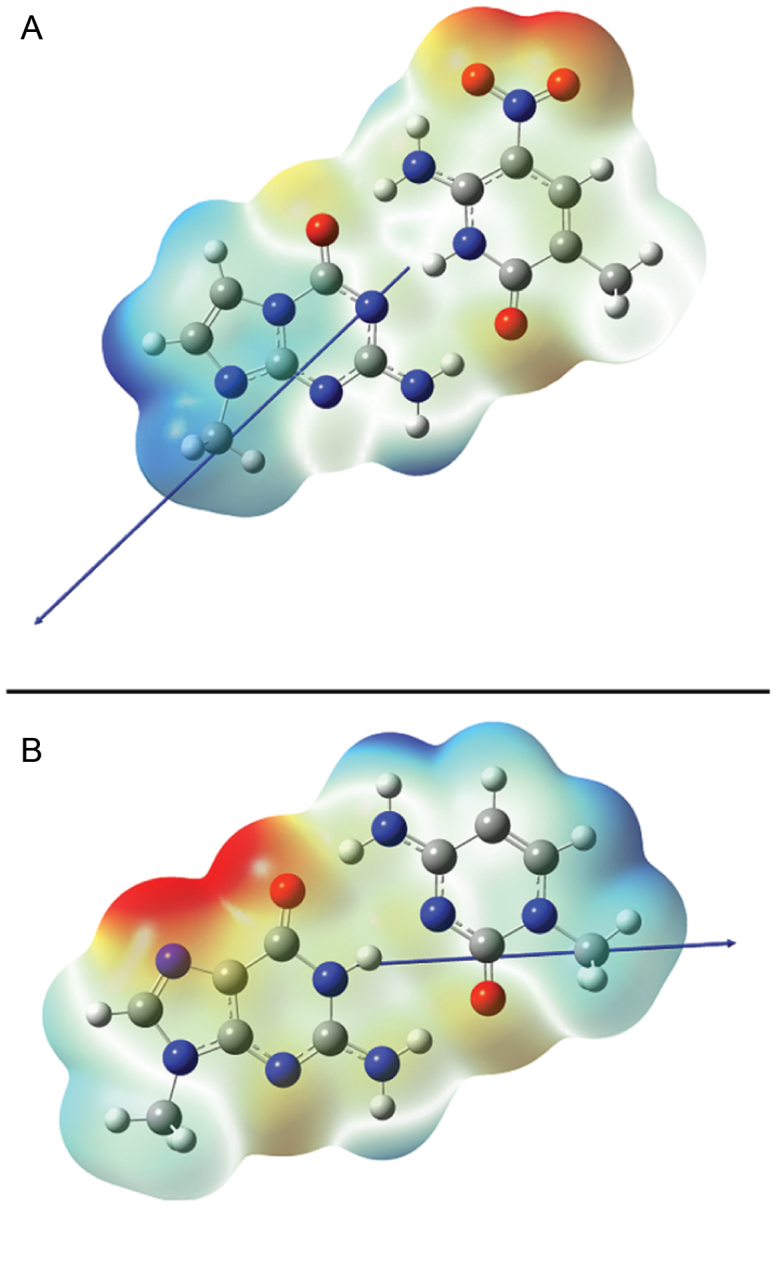
Dipole moments and the ESP for (**A**) **P:Z** and (**B**) **G:C** nucleobase pairs. Methyl substituents are used in place of C-1’ in the deoxyribose ring. The ESP is rendered on the van der Waals surface of the molecules, with values in both figures being colored over the range of ∼−40 kcal/mol (red) to ∼+40 kcal/mol (blue). Dipole moments are shown as blue arrows (a positive vector points toward positive charge density).

Using CCSD/aug’-cc-pVDZ, we calculated the dipole moment of **P:Z** and **G:C**, which is a valid descriptor of the electric potential far from the nucleobase pair (Figure 7). For **P:Z**, the gas phase dipole moment is 11.95 D, which is roughly twice that computed for **G:C** (5.99 D), with an uncertainty of 0.025 D (45,48). Importantly, the dipole vectors for the two nucleobase pairs have very different orientations, with that of **P:Z** being rotated about −135° relative to that for **G:C** (Figure 7). We also computed the electrostatic potential (ESP), which gives a qualitative indication of short-range electrostatic interactions, of the nucleobase pairs. When visualized on the van der Waals surface, significant differences in the ESP are also evident (Figure 7). Although the region of negative ESP is associated with the nitro substituent of the **Z** base, the **P** and **G** nucleobases have very different electrostatic properties in the region near to the glycosidic linkage. Similarly, it is less energetically favorable to protonate the carbonyl group of the P nucleobase, implying differences in the energy of hydrogen bonding for the two purines. Significant differences in the electronic distribution therefore exist between **P:Z** and **G:C** nucleobase pairs; this variation no doubt underlies some of the differences in the conformational preferences of the PZ-containing oligonucleotide in water.

**Figure 8.**
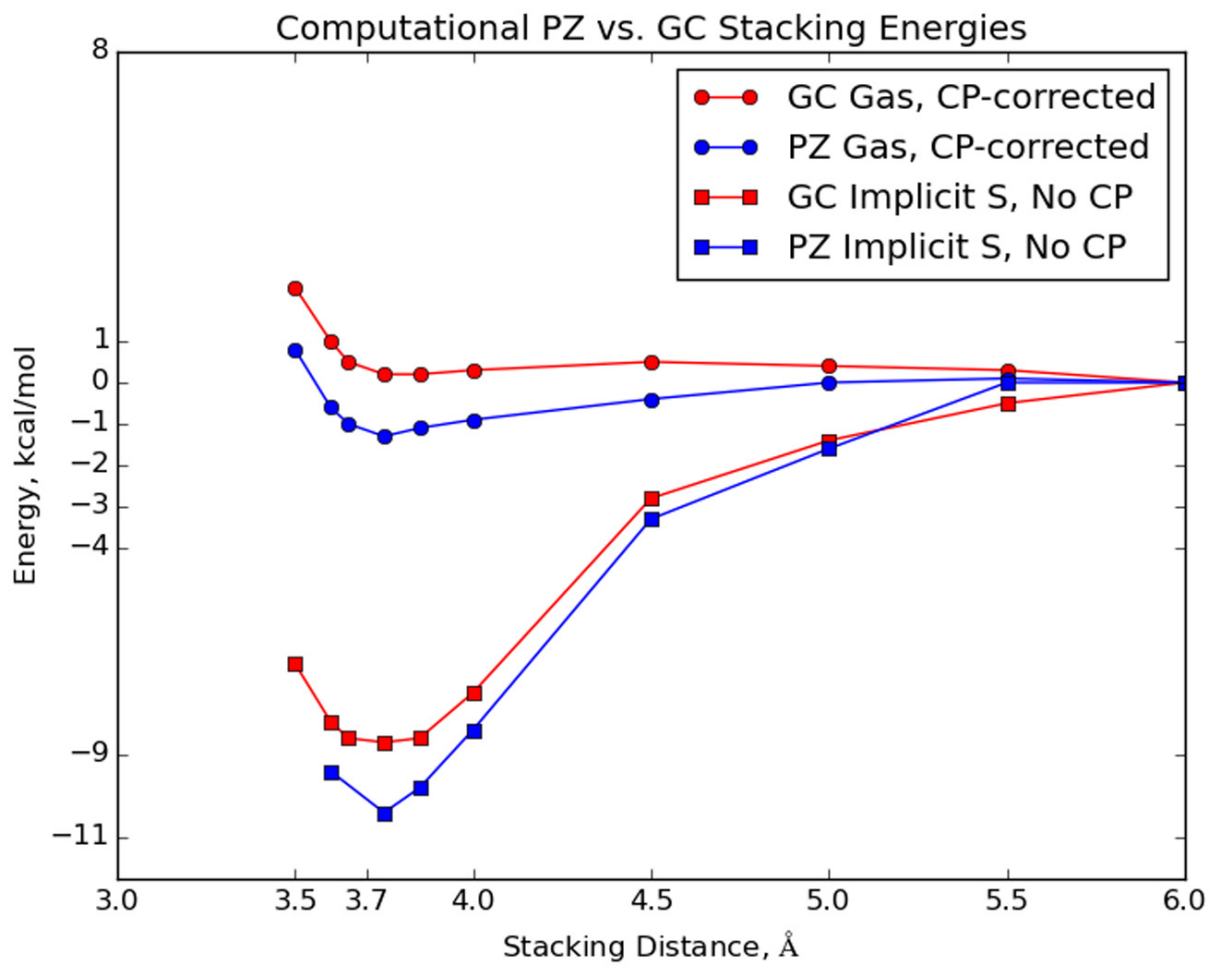
van der Waals stacking energies for stacked **PZ**-containing dinucleotide steps (blue) and corresponding GC-containing dinucleotide steps (blue) nucleobases. Electronic energies were computed using the algorithm outlined in Materials and Methods. ‘CP-corrected’ denotes counterpoise-corrected; ‘Implicit S’ denotes implicit solvent.

The dispersion and repulsion van der Waals forces between natural nucleobases are also a non-trivial component of the energetics of DNA duplexes. Indeed, studies of stacking interactions estimate that their energetic contribution is comparable, if not equal to, the stabilization energy of hydrogen bonding between Watson–Crick nucleobase pairs (76). As a consequence, we used a van der Waals-corrected KS-DFT formalism to determine the difference in the van der Waals interactions of **P:Z** and **G:C** nucleobase pairs. Thus, we calculated the interaction energy of all **PZ**-containing dinucleotide steps and corresponding GC-containing dinucleotide steps as a function of the distance between the nucleobase pairs. Interestingly, the interaction of **PZ**-containing pairs is calculated to be more stabilizing than that of GC-containing pairs (Figure 8); this difference in stabilization will be additive for the multiple, consecutive **P:Z** nucleobase pairs in the 16-bp duplex studied in these MD simulations. Wang *et al.* (20) also observed greater stabilization when a **P:Z** pair was involved in stacking interactions with G:C base pairs (**PZ**/GC and **PZ**/CG). These authors did not, however, investigate stacking of solely PZ-containing dinucleotide steps. One can see that whether one considers the counterpoise-corrected gas phase values or the implicit-solvent system, **PZ**-containing dinucleotide steps are far more stabilized than GC-containing steps.

Given these differences in van der Waals interaction energies, we next determined the free energy of hydrogen bonding in the **P:Z** and **G:C** nucleobase pairs (the Watson–Crick binding energy). We reproduced prior estimates of ΔH_298_ for the Watson–Crick hydrogen bonding energy of the **G:C** base pair (77–79). These calculations show that the Gibbs free energy associated with Watson–Crick pairing for **G:C** is more stable than **P:Z** by 1.4 kcal/mol (Table 4). This energetic difference comes mostly from the entropic contribution; the electronic/enthalpy energies are effectively identical. It therefore should be easier to disrupt hydrogen bonding in the **P:Z** nucleobase pair compared to **G:C**. For our calculations we used CCSD(T), which gives an essential exact treatment of dynamic correlation, and aug’-cc-pVTZ, which is a very large basis expansion. Our electronic energies of −29.8 kcal/mol and −30.3 kcal/mol for **P:Z** and G:C, respectively, differ from previously reported values −26 kcal/mol and −26 kcal/mol, respectively, obtained using a more approximate KS-DFT calculations of the hydrogen-bonding energy (20). Thus, M05-2X/6-31+G(d,p) gas-phase energies differ from our calculations by about 4 kcal/mol in estimating the stability. Their implicit-solvent calculations predict effectively isoelectronic energies again (−15 kcal/mol and −15 kcal/mol). The same trends (−28 for each in the gas phase, −13 kcal/mol for each in implicit solvent representation), were observed in a second report, albeit for different functional, basis choice and implicit solvent model (19).

**Table 4.** Watson–Crick pair hydrogen bonding energy contributions (kcal.mol) for **P:Z** and **G:C** nucleobase pairs

	P:Z	G:C	Δ
ΔU (Electronic)	−29.8	−30.3	+0.5
ΔH	−28.0	−27.4	−0.6
−TΔS	13.0	11.0	+2.0
ΔG	−15.0	−16.4	+1.4

**Figure 9.**
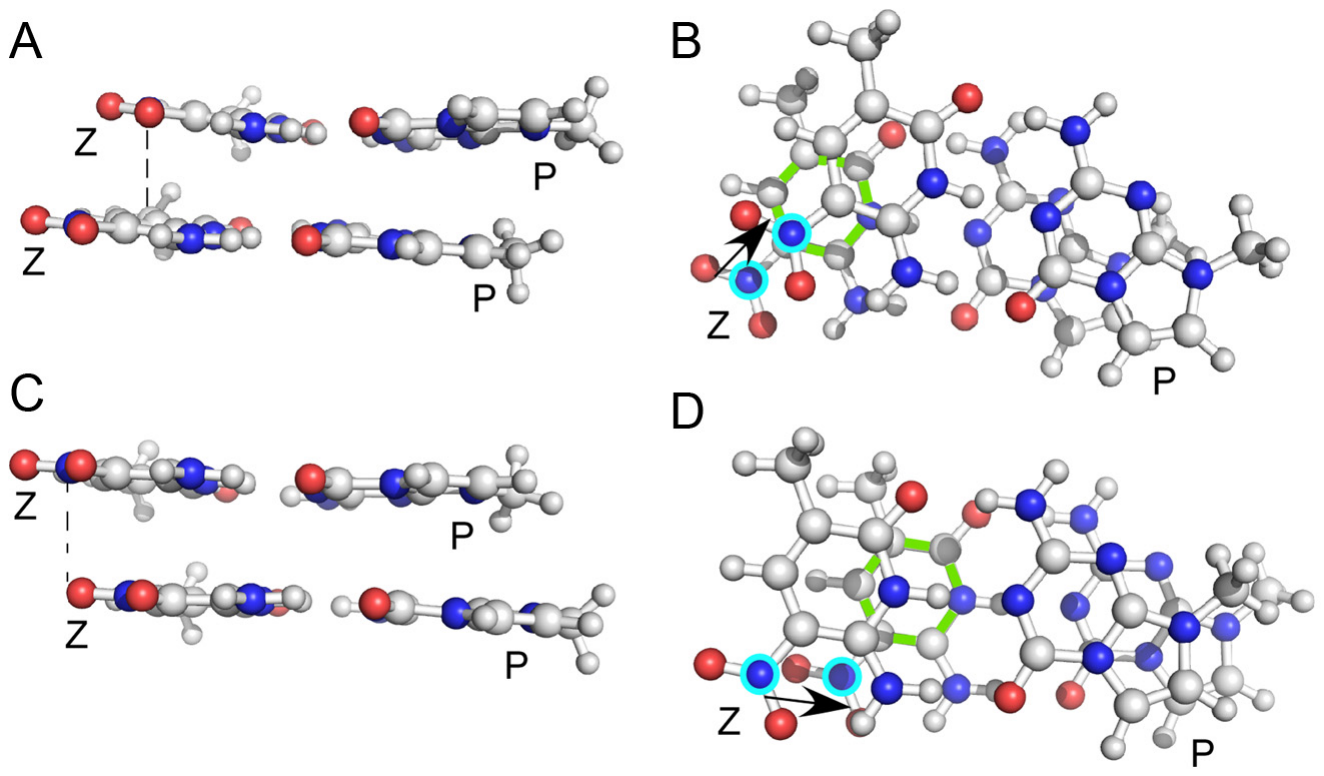
Comparison of energetically favorable *slide* (**A**) and (**B**) and *shift* (**C**) and (**D**) stacking interactions for **PP/ZZ** dinucleotide steps in the PZ oligonucleotide. Ball-and-stick representations of two stack **P:Z** pairs are shown without the associated deoxyribose sugars and associated phosphodiester backbone atoms. C atoms are shown in light gray, O atom in red and N atoms in blue. The N5 atoms of the Z-nitro groups are encircled in cyan in views (B) and (D) for clarity. The pyrimidine ring of the **Z** in the lower plane in this this view is shown with green bonds. Views shown in (B) and (D) are rotated 90° with respect to those shown in (A) and (C). The *slide* conformer represents a unique stacking conformer enabled by the nitro groups present in **Z**, which preferentially stacks over the ring of the adjacent **Z** nucleobase. Its calculated energy is 1.9 kcal/mol less than the standard *shift* conformation observed for stacking interactions in natural DNA. Dashed black lines in (A) and (C) indicate the relative stacking position for a nitro group oxygen with the adjacent **Z** nucleobase, such that (A) O is positioned over C5 of the pyrimidine ring, or (B) approximately over the nitro oxygen. Note that the two conformers are shown for **PP/ZZ** dinucleotide steps with opposite strand sense. The dinucleotide steps are oriented such that the N5 atoms of the **Z** nucleobases are positioned similarly in (A) and (C). In (B) and (D), the black arrows indicate relative directions of movement for **Z** nitro N5 atoms in the upper and lower planes of the stacked **P:Z** pairs.

In light of these observations, we turned our attention to determining *optimized* stacking interactions, in order to examine how altered dinucleotide interactions might perturb the helical preferences of the **PZ**-containing oligonucleotide. From the MD simulations, we observed a large value of the slide parameter; we thus sought conformers of the **PP/ZZ** and GG/CC dinucleotide that might correlate with the observed MD result. We investigated the energetics of stacking **PP/ZZ** dinucleotide optimized structures in the gas-phase using a M06-2X/aug’’-cc-pVDZ geometry optimization. Two conformers were identified for the **PP/ZZ** dinucleotide (Figure 9), which we term the ‘slide’ and ‘shift’ conformers because their geometries arise from displacement of one nucleobase pair along the axes corresponding to either slide or shift. The ‘slide’ conformer is more stable by 1.9 kcal/mol on the basis of M06-2X/aug’’-cc-pVTZ energies and features a staggered stacking of the NO_2_ groups, such that the nitrogen of the NO_2_ moiety in one **Z** nucleobase is placed above the carbon attached to the NO_2_ group of the other **Z**, allowing the NO_2_ group to stack over the pyrimidine ring of the adjacent Z or purine ring of the adjacent P. We observe this same structural feature repeatedly in the MD simulations of the **PZ**-containing duplex DNA rather than the alternate ‘shift’ conformer in which there is merely a vertical displacement of the NO_2_ groups. The GG/CC dinucleotide also features a preference for its ‘slide’ conformation, but there is a big difference between it and the **PP/ZZ** dinucleotide. The stabilizing attraction energy (defined as the difference in the dinucleotide energy minus twice the mononucleotide energy) in a GG/CC dinucleotide (−16 kcal/mol) is less than in **PP/ZZ** (−18 kcal/mol), and hence greater slide values are observed for the **PP/ZZ** dinucleotide than the GG/CC analog. Independent corroboration of structural features observed during the MD simulation is provided by quantum chemistry; this is an important finding given the lack of diverse experimental data for **PZ**-containing DNA duplexes needed for extensive validation of the new force field parameters needed to model such systems. We therefore conclude that nitro group stacking is a dinucleotide property intrinsic to the nature of the van der Waals interactions of **P:Z** nucleobase pairs and results in DNA helices exhibiting larger slide values than those of similar oligonucleotides in which **P:Z** is replaced by **G:C** (Figure 2). Lastly, this stacking interaction of the NO_2_ groups may underpin the A-form properties seen in the MD simulations of the **PZ**-containing duplex and observed in the A-form crystal structure (PDB ID: 4XNO) while the shift conformation is related to that observed in the crystal structure for **PP/ZZ** dinucleotide steps in B-form (PDB ID: 4XO0). In conclusion, the MD simulations reveal a wide array of different structures made possible by combinations of *slide* and *shift* conformations for the **PZ/PZ, PP/ZZ** or**ZP/ZP** dinucleotide steps; thus, **PZ** samples more conformations than GC contributing to the observed conformational flexibility in our MD simulations.

## Supplementary Material

Supplementary Data
