# Supplementary material for: Consecutive non-natural PZ nucleobase pairs in DNA impact helical structure as seen in 50 μs molecular dynamics simulations
Source: Nucleic Acids Res. 2017 Feb 28;45(7):3643–53. doi: 10.1093/nar/gkx144 (PMC5397145; doi:10.1093/nar/gkx144)
Supplement: Supplementary Data [file gkx144_Supp.pdf]

Supporting Information

Consecutive Non-Natural PZ Nucleobase Pairs in DNA Impact Helical Structure as Seen in 50 µs Molecular Dynamics Simulations

Robert W. Molt, Jr., Millie M. Georgiadis, and Nigel G. J. Richards

School of Chemistry, Cardiff University, Park Place, Cardiff, CF10 3AT, United Kingdom, Department of Biochemistry & Molecular Biology, Indiana University School of Medicine, Indianapolis, IN 46202, United States, Department of Chemistry & Chemical Biology, Indiana University Purdue University Indianapolis, Indianapolis, IN 46202, United States, and ENSCO, Inc., 4849 North Wickham Road, Melbourne, FL 32940, United States,

Table S1. Time convergence (μs) of all parameters for the **GC**-containing oligonucleotide.

Shear	0.5	5	25	50
G-C	-0.15	-0.15	-0.15	-0.15
G-C	-0.16	-0.16	-0.16	-0.16
G-C	-0.13	-0.13	-0.13	-0.13
C-G	0.13	0.13	0.13	0.13
C-G	0.16	0.16	0.16	0.16
C-G	0.16	0.16	0.15	0.16
Stretch	0.5	5	25	50
G-C	-0.07	-0.07	-0.07	-0.07
G-C	-0.07	-0.07	-0.07	-0.07
G-C	-0.06	-0.06	-0.06	-0.06
C-G	-0.06	-0.06	-0.06	-0.06
C-G	-0.07	-0.07	-0.07	-0.07
C-G	-0.07	-0.07	-0.07	-0.07
Stagger	0.5	5	25	50
G-C	0.01	0.01	0.01	0.01
G-C	-0.11	-0.11	-0.12	-0.12
G-C	-0.13	-0.13	-0.13	-0.13
C-G	-0.13	-0.13	-0.13	-0.13
C-G	-0.11	-0.11	-0.12	-0.12
C-G	0.01	0.01	0.01	0.01
Buckle	0.5	5	25	50
G-C	3.45	3.52	3.56	3.55
G-C	-0.88	-0.72	-0.79	-0.81
G-C	-4.50	-4.30	-4.27	-4.30
C-G	4.26	4.26	4.33	4.29
C-G	0.77	0.77	0.83	0.83
C-G	-3.65	-3.47	-3.53	-3.52
Propeller	0.5	5	25	50
G-C	-5.52	-5.44	-5.48	-5.49
G-C	-8.86	-8.77	-8.84	-8.84
G-C	-6.22	-6.17	-6.15	-6.16
C-G	-6.13	-6.13	-6.16	-6.14
C-G	-8.99	-8.83	-8.85	-8.83
C-G	-5.57	-5.44	-5.48	-5.47
Opening	0.5	5	25	50
G-C	-0.53	-0.53	-0.52	-0.52
G-C	0.07	0.06	0.07	0.07
G-C	-0.48	-0.48	-0.49	-0.49
C-G	-0.51	-0.49	-0.49	-0.49

C-G	0.12	0.08	0.08	0.07
C-G	-0.51	-0.51	-0.52	-0.52

Shift	0.5	5	25	50
TG/CA	0.19	0.18	0.17	0.17
GG/CC	0.22	0.23	0.23	0.23
GG/CC	-0.11	-0.11	-0.11	-0.11
GC/GC	-0.01	0.00	0.00	0.00
CC/GG	0.12	0.11	0.11	0.11
CC/GG	-0.24	-0.24	-0.23	-0.23
CA/TG	-0.15	-0.17	-0.17	-0.17

Slide	0.5	5	25	50
TG/CA	-0.54	-0.54	-0.54	-0.54
GG/CC	-1.27	-1.25	-1.26	-1.26
GG/CC	-1.34	-1.34	-1.33	-1.33
GC/GC	-0.77	-0.78	-0.78	-0.78
CC/GG	-1.33	-1.33	-1.33	-1.33
CC/GG	-1.26	-1.26	-1.26	-1.26
CA/TG	-0.56	-0.54	-0.54	-0.54

Rise	0.5	5	25	50
TG/CA	3.26	3.26	3.25	3.25
GG/CC	3.46	3.45	3.46	3.46
GG/CC	3.42	3.42	3.42	3.42
GC/GC	3.19	3.19	3.19	3.19
CC/GG	3.42	3.42	3.42	3.42
CC/GG	3.46	3.45	3.46	3.46
CA/TG	3.25	3.25	3.25	3.25

Tilt	0.5	5	25	50
TG/CA	-0.65	-0.61	-0.55	-0.57
GG/CC	0.53	0.53	0.55	0.55
GG/CC	0.15	0.14	0.09	0.10
GC/GC	-0.01	-0.02	0.00	0.00
CC/GG	-0.08	-0.13	-0.10	-0.10
CC/GG	-0.53	-0.57	-0.56	-0.56
CA/TG	0.47	0.54	0.57	0.58

Roll	0.5	5	25	50
TG/CA	9.61	9.62	9.56	9.59
GG/CC	5.20	5.14	5.22	5.21
GG/CC	4.90	4.80	4.89	4.88
GC/GC	2.93	2.81	2.88	2.87
CC/GG	4.93	4.85	4.89	4.88
CC/GG	5.37	5.24	5.24	5.23
CA/TG	9.53	9.62	9.62	9.63

Twist	t/100	t/10	t/2	t
TG/CA	30.42	30.35	30.32	30.32
GG/CC	30.20	30.24	30.20	30.21
GG/CC	29.94	29.99	29.98	29.98
GC/GC	33.61	33.60	33.55	33.56
CC/GG	29.94	29.99	29.97	29.97
CC/GG	30.16	30.13	30.18	30.19
CA/TG	30.31	30.25	30.28	30.30

H-Rise	0.5	5	25	50
TG/CA	2.85	2.84	2.83	2.83
GG/CC	3.12	3.12	3.12	3.12
GG/CC	3.09	3.10	3.09	3.09
GC/GC	3.08	3.08	3.08	3.08
CC/GG	3.08	3.10	3.09	3.09
CC/GG	3.11	3.12	3.12	3.12
CA/TG	2.83	2.83	2.83	2.83

Inclination	0.5	5	25	50
TG/CA	17.87	17.91	17.81	17.86
GG/CC	10.07	9.96	10.11	10.09
GG/CC	9.47	9.28	9.46	9.43
GC/GC	5.19	4.98	5.11	5.09
CC/GG	9.56	9.37	9.45	9.43
CC/GG	10.41	10.17	10.15	10.13
CA/TG	17.78	17.93	17.94	17.94

Tip	0.5	5	25	50
TG/CA	1.19	1.14	1.03	1.06
GG/CC	-1.09	-1.09	-1.11	-1.12
GG/CC	-0.31	-0.28	-0.18	-0.21
GC/GC	0.01	0.03	0.00	0.00
CC/GG	0.17	0.25	0.19	0.20
CC/GG	1.09	1.16	1.14	1.13
CA/TG	-0.87	-1.00	-1.07	-1.08

H-Twist	0.5	5	25	50
TG/CA	32.90	32.83	32.78	32.78
GG/CC	31.41	31.44	31.42	31.42
GG/CC	31.05	31.09	31.09	31.09
GC/GC	34.32	34.30	34.26	34.27
CC/GG	31.06	31.09	31.08	31.08
CC/GG	31.40	31.35	31.40	31.40
CA/TG	32.78	32.74	32.76	32.78

Major Groove Refined	0.5	5	25	50
TG/CA	20	20	20	20
GG/CC	20	20	20	20
GG/CC	21	21	21	21
GC/GC	21	21	21	21
CC/GG	21	21	21	21
CC/GG	20	20	20	20
CA/TG	20	20	20	20

Minor Groove Refined	0.5	5	25	50
TG/CA	14	14	14	14
GG/CC	13	13	13	13
GG/CC	13	13	13	13
GC/GC	13	13	13	13
CC/GG	13	13	13	13
CC/GG	13	13	13	13
CA/TG	14	14	14	14

Major Groove Unrefined	0.5	5	25	50
TG/CA	21	21	21	21
GG/CC	21	21	21	21
GG/CC	22	22	21	22
GC/GC	22	22	22	22
CC/GG	21	22	21	22
CC/GG	21	21	21	21
CA/TG	21	21	21	21

Minor Groove Unrefined	0.5	5	25	50
TG/CA	14	14	14	14
GG/CC	14	14	14	14
GG/CC	14	14	14	14
GC/GC	13	13	13	13
CC/GG	14	14	14	14
CC/GG	14	14	14	14
CA/TG	14	14	14	14

Table S2. Time convergence (μs) of all parameters for the **PZ**-containing oligonucleotide.

Shear		0.5	5	25	50
6=27	P-Z	-0.06	-0.05	-0.06	-0.05
7=26	P-Z	-0.04	-0.04	-0.04	-0.04
8=25	P-Z	0.04	-0.39	-0.21	-0.22
9=24	Z-P	-0.05	0.01	0.00	0.10
10=23	Z-P	0.04	0.04	0.04	0.04
11=22	Z-P	0.05	0.06	0.06	0.06

Stretch		0.5	5	25	50
6=27	P-Z	-0.04	-0.04	-0.04	-0.04
7=26	P-Z	-0.03	-0.03	-0.03	-0.03
8=25	P-Z	-0.04	-0.11	-0.06	-0.05
9=24	Z-P	-0.04	-0.04	-0.04	-0.04
10=23	Z-P	-0.03	-0.03	-0.03	-0.03
11=22	Z-P	-0.04	-0.04	-0.04	-0.04

Stagger		0.5	5	25	50
6=27	P-Z	-0.23	-0.23	-0.24	-0.24
7=26	P-Z	-0.11	-0.10	-0.11	-0.11
8=25	P-Z	-0.22	-0.21	-0.20	-0.21
9=24	Z-P	-0.22	-0.19	-0.20	-0.21
10=23	Z-P	-0.14	-0.11	-0.12	-0.11
11=22	Z-P	-0.23	-0.24	-0.24	-0.24

Buckle		0.5	5	25	50
6=27	P-Z	-5.96	-6.17	-6.15	-6.20
7=26	P-Z	-3.34	-3.65	-3.68	-3.71
8=25	P-Z	-3.18	-2.85	-2.93	-3.13
9=24	Z-P	3.29	2.90	3.22	3.13
10=23	Z-P	4.24	3.61	3.72	3.70
11=22	Z-P	5.58	6.26	6.29	6.29

Propeller		0.5	5	25	50
6=27	P-Z	-6.60	-6.72	-6.68	-6.70
7=26	P-Z	-3.31	-3.36	-3.27	-3.31
8=25	P-Z	-0.20	0.97	0.53	0.51
9=24	Z-P	-0.35	-0.66	-0.57	-0.03
10=23	Z-P	-3.15	-3.32	-3.28	-3.32
11=22	Z-P	-6.90	-6.67	-6.69	-6.69

Opening		0.5	5	25	50
6=27	P-Z	-2.25	-2.30	-2.27	-2.28
7=26	P-Z	-3.37	-3.39	-3.35	-3.35
8=25	P-Z	-3.57	-1.72	-2.21	-2.21
9=24	Z-P	-3.59	-3.46	-3.49	-2.77
10=23	Z-P	-3.21	-3.35	-3.33	-3.33
11=22	Z-P	-2.44	-2.26	-2.26	-2.25

Shift		0.5	5	25	50
5=28/6=27	TP/ZA	0.60	0.60	0.59	0.59
6=27/7=26	PP/ZZ	0.56	0.56	0.57	0.57
7=26/8=25	PP/ZZ	1.02	1.26	1.17	1.16
8=25/9=24	PZ/PZ	-0.01	-0.17	-0.10	-0.04
9=24/10=23	ZZ/PP	-1.00	-0.98	-1.02	-1.09
10=23/11=22	ZZ/PP	-0.54	-0.58	-0.58	-0.57

11=22/12=21	ZA/TP	-0.61	-0.59	-0.59	-0.59
Slide		0.50	5	25	50
5=28/6=27	TP/ZA	-1.73	-1.72	-1.72	-1.72
6=27/7=26	PP/ZZ	-1.99	-1.98	-1.99	-1.98
7=26/8=25	PP/ZZ	-2.46	-2.64	-2.55	-2.55
8=25/9=24	PZ/PZ	-2.83	-2.98	-2.88	-2.86
9=24/10=23	ZZ/PP	-2.36	-2.40	-2.42	-2.48
10=23/11=22	ZZ/PP	-1.96	-1.98	-1.98	-1.98
11=22/12=21	ZA/TP	-1.68	-1.72	-1.72	-1.72
Rise		0.5	5	25	50
5=28/6=27	TP/ZA	3.77	3.77	3.77	3.77
6=27/7=26	PP/ZZ	3.32	3.33	3.33	3.33
7=26/8=25	PP/ZZ	3.46	3.46	3.45	3.45
8=25/9=24	PZ/PZ	3.31	3.30	3.30	3.29
9=24/10=23	ZZ/PP	3.44	3.44	3.45	3.45
10=23/11=22	ZZ/PP	3.36	3.33	3.33	3.33
11=22/12=21	ZA/TP	3.77	3.78	3.78	3.78
Tilt		0.5	5	25	50
5=28/6=27	TP/ZA	1.56	1.63	1.66	1.68
6=27/7=26	PP/ZZ	-0.19	-0.11	-0.07	-0.05
7=26/8=25	PP/ZZ	1.57	0.80	1.15	1.19
8=25/9=24	PZ/PZ	0.00	0.58	0.21	0.15
9=24/10=23	ZZ/PP	-1.30	-1.18	-1.44	-1.31
10=23/11=22	ZZ/PP	-0.05	0.06	0.03	0.04
11=22/12=21	ZA/TP	-1.56	-1.59	-1.60	-1.63
Roll		0.5	5	25	50
5=28/6=27	TP/ZA	5.38	5.36	5.30	5.33
6=27/7=26	PP/ZZ	5.24	5.08	5.14	5.16
7=26/8=25	PP/ZZ	3.92	2.91	3.55	3.60
8=25/9=24	PZ/PZ	2.76	3.67	3.19	3.24
9=24/10=23	ZZ/PP	4.48	3.85	4.01	3.81
10=23/11=22	ZZ/PP	5.08	5.18	5.22	5.22
11=22/12=21	ZA/TP	5.20	5.38	5.40	5.39
Twist		0.5	5	25	50
5=28/6=27	TP/ZA	28.62	28.74	28.75	28.76
6=27/7=26	PP/ZZ	26.60	26.52	26.43	26.41
7=26/8=25	PP/ZZ	24.27	21.91	22.70	22.65
8=25/9=24	PZ/PZ	21.35	19.91	21.11	21.63
9=24/10=23	ZZ/PP	24.31	24.60	24.24	23.45
10=23/11=22	ZZ/PP	26.85	26.33	26.35	26.36
11=22/12=21	ZA/TP	28.28	28.81	28.79	28.80

H-Rise		0.5	5	25	50
5=28/6=27	TP/ZA	3.31	3.31	3.31	3.31
6=27/7=26	PP/ZZ	2.82	2.84	2.84	2.84
7=26/8=25	PP/ZZ	3.01	2.96	2.99	2.97
8=25/9=24	PZ/PZ	3.16	3.14	3.13	3.11
9=24/10=23	ZZ/PP	2.94	2.98	2.99	2.98
10=23/11=22	ZZ/PP	2.88	2.83	2.83	2.83
11=22/12=21	ZA/TP	3.32	3.32	3.32	3.32

Inclination		0.5	5	25	50
5=28/6=27	TP/ZA	10.57	10.49	10.37	10.43
6=27/7=26	PP/ZZ	11.23	10.89	11.02	11.08
7=26/8=25	PP/ZZ	8.95	8.93	9.01	9.21
8=25/9=24	PZ/PZ	2.59	2.09	2.73	2.98
9=24/10=23	ZZ/PP	10.11	8.94	8.94	9.16
10=23/11=22	ZZ/PP	10.76	11.14	11.21	11.20
11=22/12=21	ZA/TP	10.30	10.52	10.56	10.53

Tip		0.5	5	25	50
5=28/6=27	TP/ZA	-3.12	-3.26	-3.32	-3.34
6=27/7=26	PP/ZZ	0.00	-0.23	-0.37	-0.42
7=26/8=25	PP/ZZ	-5.10	-4.87	-5.07	-5.02
8=25/9=24	PZ/PZ	-0.03	-0.10	-0.06	-0.05
9=24/10=23	ZZ/PP	4.34	3.92	4.47	4.70
10=23/11=22	ZZ/PP	0.56	0.39	0.47	0.44
11=22/12=21	ZA/TP	3.13	3.17	3.20	3.24

H-Twist		0.5	5	25	50
5=28/6=27	TP/ZA	30.19	30.30	30.32	30.33
6=27/7=26	PP/ZZ	28.08	28.01	27.95	27.94
7=26/8=25	PP/ZZ	26.34	23.76	24.64	24.58
8=25/9=24	PZ/PZ	21.70	20.24	21.53	22.11
9=24/10=23	ZZ/PP	26.42	26.52	26.30	25.42
10=23/11=22	ZZ/PP	28.31	27.85	27.89	27.90
11=22/12=21	ZA/TP	29.82	30.37	30.36	30.37

Major_Groove_Refined	0.5	5	25	50
TP/ZA	21.5	21.4	21.4	21.4
PP/ZZ	23.6	23.5	23.5	23.5
PP/ZZ	26.8	26.8	26.7	26.7
PZ/PZ	26.9	26.9	26.9	26.8
ZZ/PP	26.6	26.5	26.6	26.6
ZZ/PP	23.6	23.5	23.5	23.5
ZA/TP	21.3	21.4	21.3	21.3

Minor_Groove_Refined	0.5	5	25	50
TP/ZA	13.4	13.4	13.4	13.4

PP/ZZ	14.1	14.2	14.2	14.2
PP/ZZ	13.9	14.0	14.0	14.0
PZ/PZ	13.4	13.4	13.4	13.4
ZZ/PP	14.0	14.0	14.0	14.0
ZZ/PP	14.1	14.1	14.1	14.1
ZA/TP	13.3	13.3	13.3	13.4

Major_Groove_Unrefined	0.5	5	25	50
TP/ZA	22.4	22.3	22.3	22.3
PP/ZZ	24.5	24.7	24.6	24.6
PP/ZZ	28.3	28.8	28.5	28.5
PZ/PZ	29.5	30.0	29.7	29.8
ZZ/PP	28.1	28.5	28.4	28.4
ZZ/PP	24.4	24.5	24.4	24.5

Minor_Groove_Unrefined	0.5	5	25	50
TP/ZA	14.3	14.4	14.3	14.3
PP/ZZ	15.0	15.1	15.0	15.0
PP/ZZ	14.7	14.7	14.7	14.7
PZ/PZ	14.1	14.1	14.1	14.1
ZZ/PP	14.7	14.8	14.7	14.7
ZZ/PP	14.9	15.0	15.0	15.0
ZA/TP	14.2	14.3	14.3	14.3

Table S3. Confidence intervals for selected helical parameters of the PZ-containing oligonucleotide.

Average values of local parameters

Shear		Average	SEM	Interval
1=32	C-G	0.02	0.04	0.077
2=31	T-A	0.02	0.01	0.021
3=30	T-A	-0.01	0.01	0.022
4=29	A-T	0.00	0.00	0.001
5=28	T-A	-0.03	0.00	0.001
6=27	P-Z	-0.05	0.00	0.001
7=26	P-Z	-0.04	0.00	0.002
8=25	P-Z	-0.22	0.09	0.170
9=24	Z-P	0.10	0.07	0.143
10=23	Z-P	0.04	0.00	0.002
11=22	Z-P	0.06	0.00	0.003
12=21	A-T	0.03	0.00	0.001
13=20	T-A	0.00	0.00	0.001
14=19	A-T	0.00	0.02	0.033
15=18	A-T	-0.03	0.02	0.041
16=17	G-C	-0.09	0.05	0.089
Stretch				
1=32	C-G	-0.37	0.06	0.117
2=31	T-A	-0.06	0.03	0.058
3=30	T-A	-0.02	0.01	0.025
4=29	A-T	0.01	0.00	0.000
5=28	T-A	0.02	0.00	0.000
6=27	P-Z	-0.04	0.00	0.001
7=26	P-Z	-0.03	0.00	0.001
8=25	P-Z	-0.05	0.01	0.017
9=24	Z-P	-0.04	0.00	0.006
10=23	Z-P	-0.03	0.00	0.001
11=22	Z-P	-0.04	0.00	0.001
12=21	A-T	0.02	0.00	0.000
13=20	T-A	0.01	0.00	0.000
14=19	A-T	0.01	0.03	0.063
15=18	A-T	-0.03	0.06	0.110
16=17	G-C	0.24	0.07	0.130
Stagger				
1=32	C-G	0.18	0.04	0.071
2=31	T-A	0.14	0.02	0.030
3=30	T-A	-0.18	0.01	0.015
4=29	A-T	-0.05	0.00	0.002
5=28	T-A	-0.08	0.00	0.002
6=27	P-Z	-0.24	0.00	0.002

7=26	P-Z	-0.11	0.00	0.005
8=25	P-Z	-0.21	0.01	0.016
9=24	Z-P	-0.21	0.01	0.013
10=23	Z-P	-0.11	0.00	0.004
11=22	Z-P	-0.24	0.00	0.002
12=21	A-T	-0.09	0.00	0.002
13=20	T-A	-0.06	0.00	0.003
14=19	A-T	-0.22	0.03	0.066
15=18	A-T	0.13	0.04	0.080
16=17	G-C	0.08	0.06	0.111
Buckle				
1=32	C-G	-1.32	0.23	0.453
2=31	T-A	-4.69	0.07	0.136
3=30	T-A	-0.44	0.13	0.260
4=29	A-T	-7.71	0.07	0.134
5=28	T-A	9.73	0.02	0.046
6=27	P-Z	-6.20	0.06	0.126
7=26	P-Z	-3.71	0.04	0.081
8=25	P-Z	-3.13	0.14	0.273
9=24	Z-P	3.13	0.09	0.185
10=23	Z-P	3.70	0.04	0.070
11=22	Z-P	6.29	0.07	0.134
12=21	A-T	-9.95	0.03	0.058
13=20	T-A	7.14	0.09	0.170
14=19	A-T	0.72	0.23	0.448
15=18	A-T	5.42	0.21	0.419
16=17	G-C	0.69	0.16	0.323
Propeller				
1=32	C-G	-10.51	0.28	0.542
2=31	T-A	-12.59	0.14	0.266
3=30	T-A	-11.34	0.06	0.125
4=29	A-T	-6.97	0.06	0.116
5=28	T-A	-8.40	0.05	0.098
6=27	P-Z	-6.70	0.02	0.045
7=26	P-Z	-3.31	0.05	0.094
8=25	P-Z	0.51	0.32	0.634
9=24	Z-P	-0.03	0.32	0.620
10=23	Z-P	-3.32	0.05	0.101
11=22	Z-P	-6.69	0.03	0.051
12=21	A-T	-8.62	0.06	0.119
13=20	T-A	-7.02	0.09	0.176
14=19	A-T	-10.25	0.24	0.478
15=18	A-T	-12.49	0.20	0.393
16=17	G-C	-11.71	0.27	0.534

Opening				
1=32	C-G	0.26	0.50	0.987
2=31	T-A	0.45	0.08	0.164
3=30	T-A	0.61	0.22	0.429
4=29	A-T	0.73	0.01	0.023
5=28	T-A	-0.14	0.01	0.025
6=27	P-Z	-2.28	0.01	0.019
7=26	P-Z	-3.35	0.01	0.018
8=25	P-Z	-2.21	0.55	1.070
9=24	Z-P	-2.77	0.48	0.941
10=23	Z-P	-3.33	0.01	0.018
11=22	Z-P	-2.25	0.01	0.029
12=21	A-T	-0.12	0.01	0.027
13=20	T-A	0.62	0.01	0.029
14=19	A-T	0.53	0.30	0.583
15=18	A-T	1.22	0.45	0.880
16=17	G-C	-7.88	0.98	1.923

Average values of dinucleotide step parameters

Shift		Average	SEM	Interval
1=32/2=31	CT/AG	0.09	0.02	0.03
2=31/3=30	TT/AA	0.14	0.01	0.01
3=30/4=29	TA/TA	0.06	0.02	0.04
4=29/5=28	AT/AT	0.06	0.00	0.00
5=28/6=27	TP/ZA	0.59	0.00	0.00
6=27/7=26	PP/ZZ	0.57	0.00	0.01
7=26/8=25	PP/ZZ	1.16	0.05	0.10
8=25/9=24	PZ/PZ	-0.04	0.05	0.09
9=24/10=23	ZZ/PP	-1.09	0.05	0.09
10=23/11=22	ZZ/PP	-0.57	0.00	0.01
11=22/12=21	ZA/TP	-0.59	0.00	0.00
12=21/13=20	AT/AT	-0.09	0.00	0.00
13=20/14=19	TA/TA	0.03	0.04	0.08
14=19/15=18	AA/TT	-0.17	0.02	0.04
15=18/16=17	AG/CT	-0.04	0.02	0.05
Slide				
1=32/2=31	CT/AG	-0.77	0.02	0.05
3=30/4=29	TA/TA	-0.21	0.01	0.01
4=29/5=28	AT/AT	-0.68	0.00	0.00
5=28/6=27	TP/ZA	-1.72	0.00	0.00
6=27/7=26	PP/ZZ	-1.98	0.00	0.00
7=26/8=25	PP/ZZ	-2.55	0.05	0.09
8=25/9=24	PZ/PZ	-2.86	0.03	0.07
9=24/10=23	ZZ/PP	-2.48	0.04	0.08

10=23/11=22	ZZ/PP	-1.98	0.00	0.00
11=22/12=21	ZA/TP	-1.72	0.00	0.01
12=21/13=20	AT/AT	-0.68	0.00	0.00
13=20/14=19	TA/TA	-0.14	0.02	0.03
14=19/15=18	AA/TT	-0.31	0.03	0.05
15=18/16=17	AG/CT	-1.02	0.03	0.07
Rise				
1=32/2=31	CT/AG	3.22	0.05	0.10
2=31/3=30	TT/AA	3.24	0.01	0.01
3=30/4=29	TA/TA	3.48	0.02	0.04
4=29/5=28	AT/AT	3.00	0.00	0.00
5=28/6=27	TP/ZA	3.77	0.00	0.00
6=27/7=26	PP/ZZ	3.33	0.00	0.00
7=26/8=25	PP/ZZ	3.45	0.01	0.01
8=25/9=24	PZ/PZ	3.29	0.01	0.02
9=24/10=23	ZZ/PP	3.45	0.00	0.01
10=23/11=22	ZZ/PP	3.33	0.00	0.00
11=22/12=21	ZA/TP	3.78	0.00	0.00
12=21/13=20	AT/AT	3.00	0.00	0.00
13=20/14=19	TA/TA	3.48	0.02	0.05
14=19/15=18	AA/TT	3.23	0.03	0.06
15=18/16=17	AG/CT	3.23	0.05	0.10
Tilt				
1=32/2=31	CT/AG	0.75	0.26	0.51
2=31/3=30	TT/AA	1.93	0.27	0.52
3=30/4=29	TA/TA	-0.83	0.37	0.73
4=29/5=28	AT/AT	-0.04	0.01	0.02
5=28/6=27	TP/ZA	1.68	0.01	0.02
6=27/7=26	PP/ZZ	-0.05	0.02	0.05
7=26/8=25	PP/ZZ	1.19	0.13	0.25
8=25/9=24	PZ/PZ	0.147	0.06	0.11
9=24/10=23	ZZ/PP	-1.31	0.10	0.20
10=23/11=22	ZZ/PP	0.04	0.02	0.04
11=22/12=21	ZA/TP	-1.63	0.01	0.02
12=21/13=20	AT/AT	0.07	0.01	0.03
13=20/14=19	TA/TA	1.63	0.23	0.45
14=19/15=18	AA/TT	-2.64	0.31	0.62
15=18/16=17	AG/CT	-0.56	0.28	0.55
Roll				
1=32/2=31	CT/AG	1.00	0.45	0.88
2=31/3=30	TT/AA	-0.17	0.03	0.06
3=30/4=29	TA/TA	4.66	0.29	0.57
4=29/5=28	AT/AT	1.30	0.02	0.04
5=28/6=27	TP/ZA	5.33	0.02	0.05

6=27/7=26	PP/ZZ	5.16	0.02	0.05
7=26/8=25	PP/ZZ	3.60	0.14	0.28
8=25/9=24	PZ/PZ	3.24	0.18	0.35
9=24/10=23	ZZ/PP	3.81	0.15	0.29
10=23/11=22	ZZ/PP	5.22	0.02	0.04
11=22/12=21	ZA/TP	5.39	0.03	0.05
12=21/13=20	AT/AT	1.23	0.02	0.04
13=20/14=19	TA/TA	4.72	0.15	0.28
14=19/15=18	AA/TT	0.43	0.14	0.27
15=18/16=17	AG/CT	0.52	0.53	1.04
Twist				
1=32/2=31	CT/AG	32.51	0.41	0.81
2=31/3=30	TT/AA	34.06	0.08	0.16
3=30/4=29	TA/TA	33.69	0.05	0.11
4=29/5=28	AT/AT	31.13	0.03	0.06
5=28/6=27	TP/ZA	28.76	0.02	0.04
6=27/7=26	PP/ZZ	26.41	0.04	0.07
7=26/8=25	PP/ZZ	22.65	0.59	1.15
8=25/9=24	PZ/PZ	21.63	0.53	1.04
9=24/10=23	ZZ/PP	23.45	0.52	1.01
10=23/11=22	ZZ/PP	26.36	0.03	0.07
11=22/12=21	ZA/TP	28.80	0.02	0.04
12=21/13=20	AT/AT	31.21	0.03	0.07
13=20/14=19	TA/TA	34.01	0.18	0.36
14=19/15=18	AA/TT	33.27	0.18	0.34
15=18/16=17	AG/CT	37.18	0.62	1.21

Average values of helix parameters

H-Rise				
1=32/2=31	CT/AG	3.13	0.05	0.10
2=31/3=30	TT/AA	3.17	0.01	0.02
3=30/4=29	TA/TA	3.25	0.02	0.03
4=29/5=28	AT/AT	2.90	0.00	0.00
5=28/6=27	TP/ZA	3.31	0.00	0.00
6=27/7=26	PP/ZZ	2.84	0.00	0.01
7=26/8=25	PP/ZZ	2.97	0.01	0.02
8=25/9=24	PZ/PZ	3.11	0.02	0.04
9=24/10=23	ZZ/PP	2.98	0.01	0.02
10=23/11=22	ZZ/PP	2.83	0.00	0.01
11=22/12=21	ZA/TP	3.32	0.00	0.00
12=21/13=20	AT/AT	2.91	0.00	0.00
13=20/14=19	TA/TA	3.28	0.02	0.03
14=19/15=18	AA/TT	3.15	0.03	0.05
15=18/16=17	AG/CT	3.08	0.05	0.10

Inclination				
1=32/2=31	CT/AG	1.52	0.32	0.63
2=31/3=30	TT/AA	0.13	0.03	0.06
3=30/4=29	TA/TA	9.35	0.16	0.31
4=29/5=28	AT/AT	3.01	0.04	0.08
5=28/6=27	TP/ZA	10.43	0.04	0.08
6=27/7=26	PP/ZZ	11.08	0.06	0.12
7=26/8=25	PP/ZZ	9.21	0.10	0.20
8=25/9=24	PZ/PZ	2.98	0.25	0.49
9=24/10=23	ZZ/PP	9.16	0.11	0.22
10=23/11=22	ZZ/PP	11.20	0.05	0.10
11=22/12=21	ZA/TP	10.53	0.05	0.09
12=21/13=20	AT/AT	2.86	0.04	0.08
13=20/14=19	TA/TA	8.81	0.17	0.32
14=19/15=18	AA/TT	1.22	0.09	0.18
15=18/16=17	AG/CT	2.16	0.46	0.90
Tip				
1=32/2=31	CT/AG	-1.71	0.24	0.47
2=31/3=30	TT/AA	-3.52	0.23	0.44
3=30/4=29	TA/TA	1.94	0.15	0.29
4=29/5=28	AT/AT	0.04	0.02	0.03
5=28/6=27	TP/ZA	-3.34	0.02	0.05
6=27/7=26	PP/ZZ	-0.42	0.05	0.11
7=26/8=25	PP/ZZ	-5.02	0.15	0.29
8=25/9=24	PZ/PZ	-0.05	0.04	0.09
9=24/10=23	ZZ/PP	4.70	0.14	0.28
10=23/11=22	ZZ/PP	0.44	0.05	0.10
11=22/12=21	ZA/TP	3.24	0.02	0.05
12=21/13=20	AT/AT	-0.10	0.03	0.05
13=20/14=19	TA/TA	-2.27	0.17	0.33
14=19/15=18	AA/TT	4.04	0.24	0.46
15=18/16=17	AG/CT	1.70	0.15	0.30
H-Twist				
1=32/2=31	CT/AG	33.47	0.51	1.00
2=31/3=30	TT/AA	35.02	0.10	0.19
3=30/4=29	TA/TA	35.59	0.18	0.36
4=29/5=28	AT/AT	31.86	0.03	0.05
5=28/6=27	TP/ZA	30.33	0.02	0.04
6=27/7=26	PP/ZZ	27.94	0.03	0.06
7=26/8=25	PP/ZZ	24.58	0.66	1.29
8=25/9=24	PZ/PZ	22.11	0.58	1.15
9=24/10=23	ZZ/PP	25.42	0.59	1.16
10=23/11=22	ZZ/PP	27.90	0.03	0.06
11=22/12=21	ZA/TP	30.37	0.02	0.04
12=21/13=20	AT/AT	31.93	0.03	0.06

13=20/14=19	TA/TA	35.67	0.29	0.57
14=19/15=18	AA/TT	34.39	0.24	0.47
15=18/16=17	AG/CT	39.01	0.70	1.38

Average values of groove widths

Major_Groove_Refined			
AT/AT	20.4	0.02	0.04
TP/ZA	21.4	0.02	0.04
PP/ZZ	23.5	0.02	0.04
PP/ZZ	26.7	0.04	0.07
PZ/PZ	26.8	0.03	0.05
ZZ/PP	26.6	0.04	0.08
ZZ/PP	23.5	0.02	0.04
ZA/TP	21.3	0.03	0.05
AT/AT	20.4	0.04	0.08
Minor_Groove_Refined			
AT/AT	12.9	0.01	0.02
TP/ZA	13.4	0.01	0.02
PP/ZZ	14.2	0.01	0.01
PP/ZZ	14.0	0.01	0.02
PZ/PZ	13.4	0.02	0.03
ZZ/PP	14.0	0.01	0.02
ZZ/PP	14.1	0.01	0.01
ZA/TP	13.4	0.01	0.02
AT/AT	12.9	0.01	0.03
Major_Groove_Unrefined			
TA/TA	19.7	0.02	0.03
AT/AT	21.1	0.01	0.03
TP/ZA	22.3	0.02	0.03
PP/ZZ	24.6	0.05	0.10
PP/ZZ	28.5	0.07	0.14
PZ/PZ	29.8	0.06	0.12
ZZ/PP	28.4	0.07	0.13
ZZ/PP	24.5	0.05	0.09
ZA/TP	22.2	0.02	0.04
AT/AT	21.1	0.02	0.04
TA/TA	19.7	0.02	0.05
Minor_Groove_Unrefined			
TA/TA	13.2	0.01	0.02
AT/AT	13.6	0.01	0.02
TP/ZA	14.3	0.01	0.02
PP/ZZ	15.0	0.01	0.01
PP/ZZ	14.7	0.01	0.02

PZ/PZ	14.1	0.02	0.04
ZZ/PP	14.7	0.01	0.03
ZZ/PP	15.0	0.01	0.01
ZA/TP	14.3	0.01	0.02
AT/AT	13.6	0.01	0.03
TA/TA	13.3	0.02	0.04

Table S4: Statistical Agreement for GC parameters

Mean	Pasi *et. al.*	Our Work
Shift	0	0.03969±0.00035
Slide	-0.9	-1.12290±0.00036
Rise	3.4	3.35531±0.000089
Tilt	0	0.2169±0.0011
Roll	0	4.3210±0.0019
Twist	35	31.2487±0.0026
Σ	Pasi *et. al.*	Our Work
Shift	0.6	0.6118±0.0015
Slide	0.5	0.6389±0.0024
Rise	0.3	0.31732±0.00090
Tilt	4	4.16333±0.0025
Roll	5	5.1268±0.0068
Twist	5	4.662±0.031

The most comprehensive study on natural nucleic acid helix parameters was conducted by Pasi *et. al.* (Pasi,M., Maddocks,J.H., Beveridge,D., Bishop,T.C., Case,D.A., Cheatham,T.E.I., Dans,P.D., Jayaram,B., Lankas,F., Laughton,C., *et al.* (2014) µABC: a systematic microsecond molecular dynamics study of tetranucleotide sequence effects in B-DNA. *Nucl. Acids Res.*, **42**, 12272–12283.); they studied natural nucleic acids for a full microsecond. Our study followed the same protocols as theirs within the following exceptions: They filtered out structures in which the Watson-Crick hydrogen bonds are broken from their statistics; we performed no filtering of the data. We used a cutoff of 8.5Å for non-bonded interactions, they had 9.0Å. Our calculation ran for 50 µs whereas they ran for 1 µs. All other aspects of simulation of the GC simulations are identical to their work. We did not elect to perform the filtering of non-Watson-Crick base pairs for the sake of comparison to experiments in the future. They noted a desire to convey more numerically realistic/stable helix parameters for this filtering; we wish to compare

to experiment (and true base pairs do occasionally lose their hydrogen bonds within the helix, as rare events, albeit on the ms time scale). We believe the difference in non-bonded interaction cutoff to be trivially different. The authors did not report their SEM values, and thus we cannot perform a fair comparison of statistical agreement (we do not know their uncertainty windows). We can assume that their values are the "known" values of benchmark against which to compare (although our increased sampling might argue our numbers are a better benchmark). We opt to use a 95% confidence interval for our two-sided t-test for our 150 samples (there were 150 independent trajectories run and analyzed individually), giving t=1.96. We follow the 2 significant figure convention in intervals. I here discuss the logic of digits assigned in the main paper text. In the vast majority of cases, the precision of each number (as judged by the 95% confidence interval t-test) is far greater than the accuracy of these numbers could possibly be. Consequently, I have arbitrarily truncated all lengths to 0.01Å and 0.1 degrees. We list the full precision of our numbers here to give proper statistical due diligence, but numbers in the main text are truncated for ease of reader understanding. As may be seen, we are in statistical agreement with Pasi *et. al.* for all parameters, given that our data must be rounded to one digit in both the average and the standard deviation. The one exception is slide, in which case both the average and standard deviation are in disagreement. However, -0.9Å is very close to -1.1Å in the means, and 0.5Å standard deviation is close to 0.6Å.

Table S5. Comparison of all local parameters for the **PZ**- and **GC**-containing oligonucleotides.

The left-hand column gives the Watson-Crick pair as well as its rung position in the DNA helix; each rung in the helix is given twice, once for variable PZ pair, once for the control GC helix. Each table entry gives the mean value of the parameter in question as well as its standard deviation, reflecting the range of the distribution of the parameter over time. For emphasis, this is *not* precision error; this is the distributional variation. The precision errors are based on a 95% confidence interval with respect to the standard error of the mean; this is true for all data analyzed in this paper. In each case, the standard error of this mean is at most 1° for angular quantities and at most 0.2Å for length quantities. Additionally, we note that there is a symmetry to all data. Helix rung position 6 is equivalent to position 11, as 7 is to 10 and 8 is to 9. When discussing the data, we will restrict analysis to discussing positions 6-8 for simplicity, since the same conclusions apply to the symmetry counterparts. The fact that the calculated data matches so perfectly to even as small as ±0.1° speaks to the precision of the data due to complete phase space sampling. We emphasize that the act of reporting a mean and standard deviation is *not* to imply a Gaussian distribution, merely as a descriptor of the data set. It is also of interest to note that the middle values in the helix are always slower to converge than the outer rung values (i.e., position 8 converges in all parameters, not just locally, more slowly than positions 7 and 6). It is of interest to see the particular influence of the helix rung position on each nucleotide/parameter; as there are at least 5 nucleotides before the helix end, this is not interpreted to be an edge effect.

Parameter	Shear (Å)	Stretch (Å)	Stagger (Å)	Buckle (°)	Propeller (°)	Opening (°)
P-Z(6)	-0.05±0.31	-0.04±0.12	-0.24±0.39	-6.2±10.1	-6.7±7.6	-2.5±3.2
G-C (6)	-0.15±0.30	-0.07±0.11	0.01±0.38	3.5±10.0	-5.5±7.8	-0.5±3.1
P-Z(7)	-0.04±0.32	-0.03±0.11	-0.11±0.37	-3.7±9.1	-3.3±7.7	-3.3±3.2
G-C (7)	-0.16±0.31	-0.07±0.11	-0.12±0.38	-0.8±10.0	-8.8±8.0	0.1±3.3
P-Z(8)	-0.22±0.59	-0.05±0.20	-0.21±0.48	-3.1±10.2	-0.5±9.9	-2.2±5.4
G-C (8)	-0.13±0.30	-0.06±0.11	-0.13±0.36	-4.3±10.9	-6.2±8.0	-0.5±3.2
Z-P(9)	0.10±0.42	-0.04±0.15	-0.21±0.45	3.1±9.9	0.0±9.4	-2.8±4.2
C-G (9)	0.13±0.30	-0.06±0.11	-0.13±0.36	4.3±10.9	-6.1±8.0	-0.5±3.2
Z-P(10)	0.04±0.32	-0.03±0.11	-0.11±0.37	3.7±9.1	-3.3±7.7	-3.3±3.2

C-G (10)	0.16±0.31	-0.07±0.11	-0.12±0.38	0.8±10.0	-8.8±8.0	0.1±3.3
Z-P(11)	0.06±0.32	-0.04±0.12	-0.24±0.39	6.3±10.1	-6.7±7.6	-2.3±3.3
C-G(11)	0.16±0.30	-0.07±0.11	0.01±0.38	-3.5±10.0	-5.5±7.8	-0.5±3.1

Table S6. Comparison of all step parameters for the **PZ**- and **GC**-containing oligonucleotides.

Parameter	Shift (Å)	Slide (Å)	Rise (Å)	Tilt (°)	Roll (°)	Twist (°)
TP/ZA	0.59±0.53	-1.72±0.52	3.77±0.41	1.7±4.9	5.3±6.2	28.8±4.0
TG/CA	0.17±0.67	-0.54±0.61	3.25±0.35	-0.6±4.7	9.6±6.4	30.3±5.2
PP/ZZ	0.57±0.60	-1.98±0.54	3.33±0.36	-0.1±4.7	5.2±5.6	26.4±5.2
GG/CC	0.23±0.67	-1.26±0.69	3.46±0.32	0.6±4.3	5.2±5.3	30.2±5.1
PP/ZZ	1.16±0.91	-2.55±0.92	3.45±0.44	1.2±5.9	3.6±7.5	22.6±8.5
GG/CC	-0.11±0.61	-1.33±0.66	3.42±0.33	0.1±4.3	4.9±5.1	30.0±4.6
PZ/PZ	-0.04±0.82	-2.86±1.32	3.29±0.32	0.1±4.6	3.2±5.9	21.6±15.3
GC/GC	0.00±0.55	-0.78±0.57	3.19±0.30	0.0±3.9	2.9±5.0	33.6±4.3
ZZ/PP	-1.09±0.84	-2.48±0.84	3.45±0.43	-1.3±5.7	3.8±7.2	23.4±7.4
CC/GG	0.11±0.61	1.33±0.66	3.42±0.33	-0.1±4.3	4.9±5.1	30.0±4.6
ZZ/PP	-0.57±0.60	-1.98±0.53	3.33±0.36	0.0±4.7	5.2±5.6	26.4±5.2
CC/GG	-0.23±0.67	-1.26±0.69	3.46±0.32	-0.6±4.3	5.2±5.3	30.2±5.1
ZA/TP	-0.59±0.53	-1.72±0.52	3.78±0.41	-1.6±4.9	5.4±6.2	28.8±4.1
CA/TG	-0.17±0.67	-0.54±0.61	3.25±0.35	0.6±4.7	9.6±6.4	30.3±5.2

Table S7. Comparison of all helix parameters for the **PZ**- and **GC**-containing oligonucleotides.

Parameter	H-Rise (Å)	Inclination (°)	Tip (°)	H-Twist (°)
TP/ZA	3.31±0.63	10.4±12.0	-3.3±9.7	30.3±4.0
TG/CA	2.83±0.50	17.9±12.2	1.1±8.6	32.8±4.8
PP/ZZ	2.84±0.73	11.1±12.5	-0.4±11.1	28.0±4.8
GG/CC	3.12±0.49	10.1±10.3	-1.1±8.3	31.4±4.9
PP/ZZ	2.97±1.17	9.2±17.8	-5.0±15.9	24.6±8.1
GG/CC	3.09±0.47	9.4±9.9	-0.2±8.2	31.1±4.5
PZ/PZ	3.11±1.15	3.0±16.6	0.0±12.0	22.1±16.4
GC/GC	3.08±0.36	5.1±8.6	0.0±6.8	34.3±4.2
ZZ/PP	2.98±1.14	9.2±17.7	4.7±15.5	25.4±6.8
CC/GG	3.09±0.47	9.4±9.9	0.2±8.2	31.1±4.5
ZZ/PP	2.83±0.72	11.2±12.5	0.4±11.1	27.9±4.8
CC/GG	3.12±0.49	10.1±10.3	1.1±8.3	31.4±4.9

ZA/TP	3.32±0.65	10.5±12.0	3.2±9.6	30.4±4.0
CA/TG	2.83±0.50	17.9±12.2	-1.1±8.6	32.8±4.8

Table S8. Comparison of all groove widths for the **PZ**- and **GC**-containing oligonucleotides.

Parameter	Major Groove Refined (Å)	Minor Groove Refined (Å)
TP/ZA	21.4±2.5	13.4±1.1
TG/CA	20.2±2.1	13.7±1.0
PP/ZZ	23.5±2.2	14.2±1.0
GG/CC	20.3±2.1	13.4±1.0
PP/ZZ	26.7±1.8	14.0±1.1
GG/CC	20.9±2.3	13.0±1.1
PZ/PZ	26.8±1.7	13.4±1.3
GC/GC	21.0±2.4	12.9±1.1
ZZ/PP	26.6±1.8	14.0±1.1
CC/GG	20.9±2.3	13.0±1.1
ZZ/PP	23.5±2.2	14.1±1.0
CC/GG	20.3±2.1	13.4±1.0
ZA/TP	21.3±2.5	13.4±1.1
CA/TG	20.2±2.1	13.7±1.0

Figure S1. Representative histograms for selected helical parameters for the PZ-containing (blue) and the control (red) oligonucleotide in the MD simulations. Density of state is defined as the number of structures for which the parameter falls in a defined range divided by the total number of structures sampled in each trajectory.

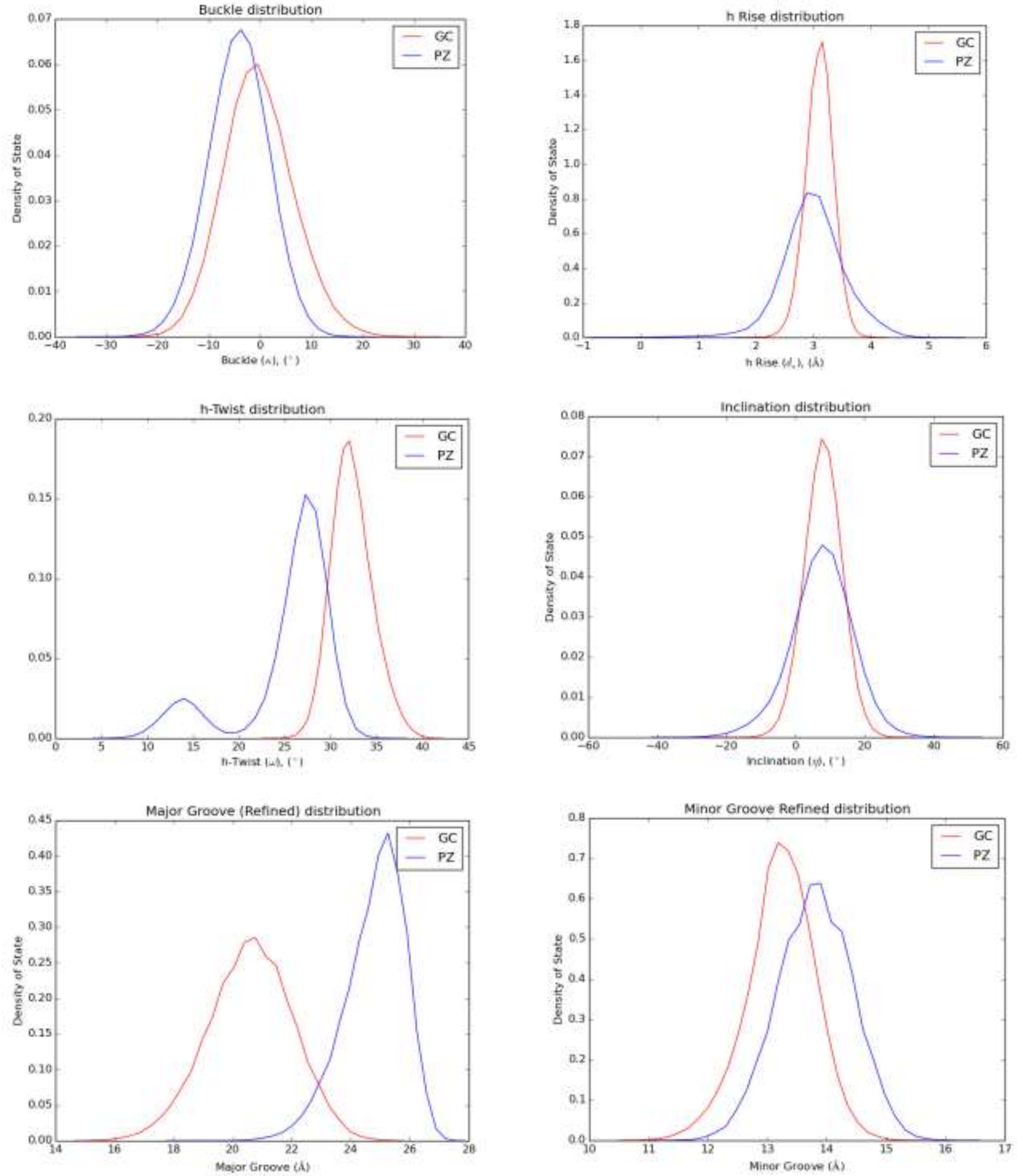

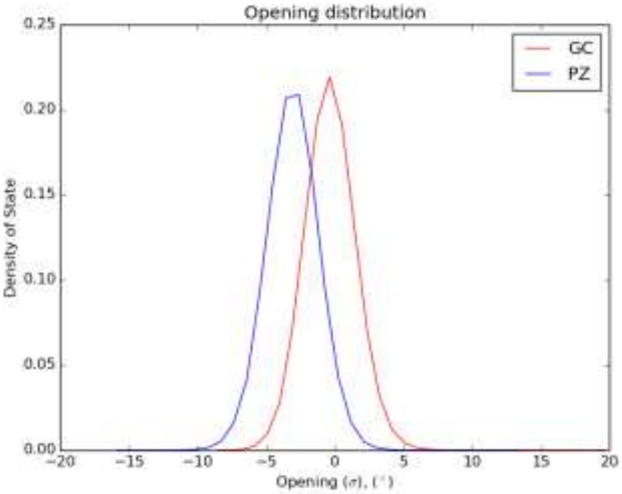
Opening distribution
GC
PZ
Density of State
Opening (σ), (°)

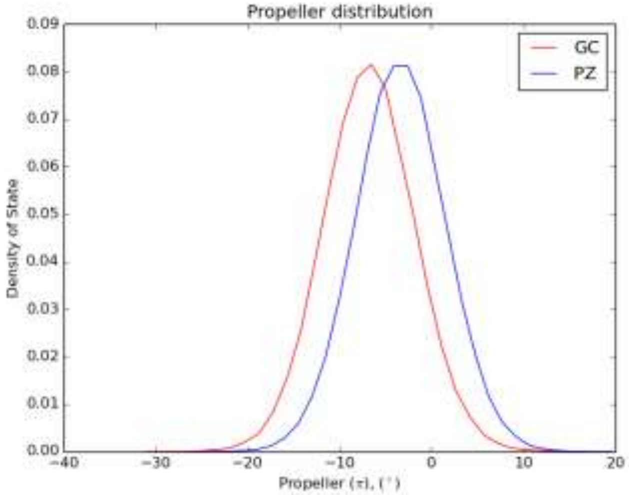
Propeller distribution
GC
PZ
Density of State
Propeller (π), (°)

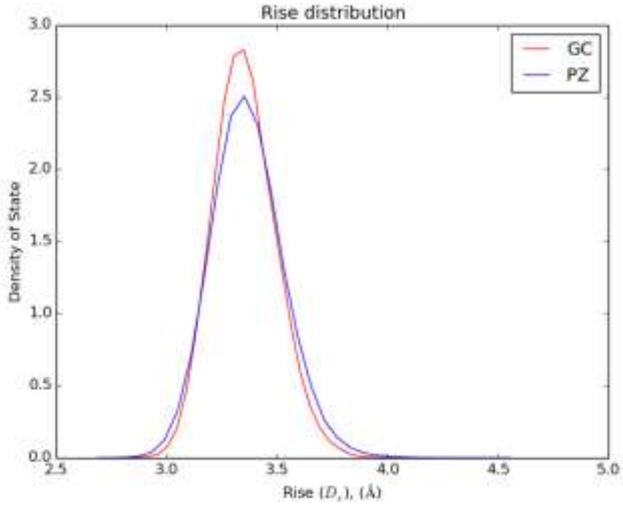
Rise distribution
GC
PZ
Density of State
Rise (D_z), (Å)

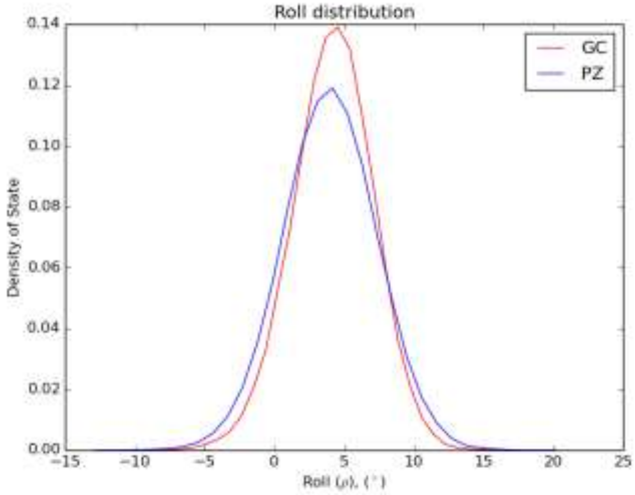
Roll distribution
GC
PZ
Density of State
Roll (ρ), (°)

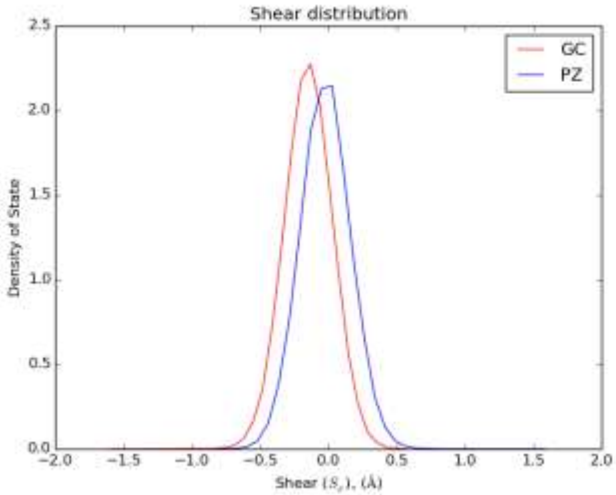
Shear distribution
GC
PZ
Density of State
Shear (S_x), (Å)

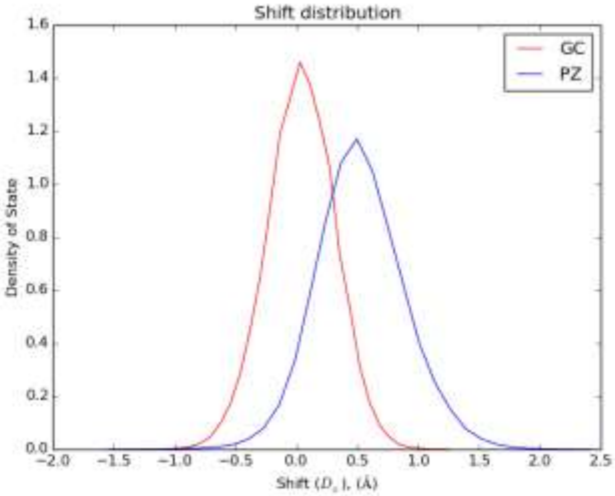
Shift distribution
GC
PZ
Density of State
Shift (D_x), (Å)

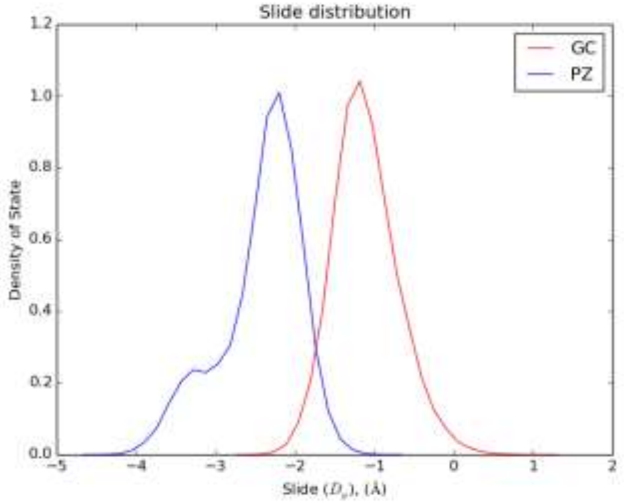
Slide distribution
GC
PZ
Density of State

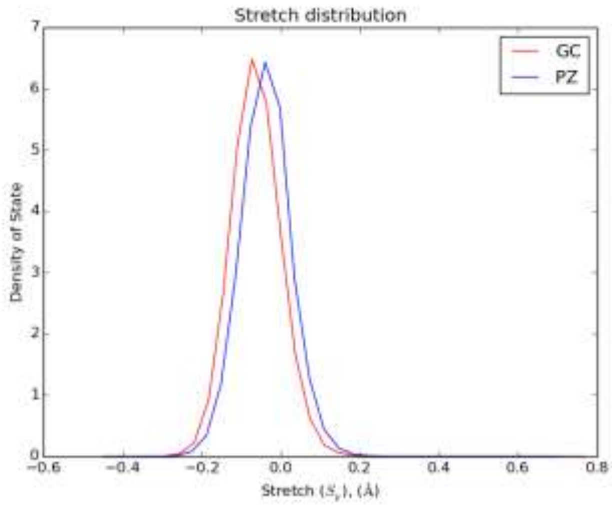
Stretch distribution
GC
PZ
Density of State

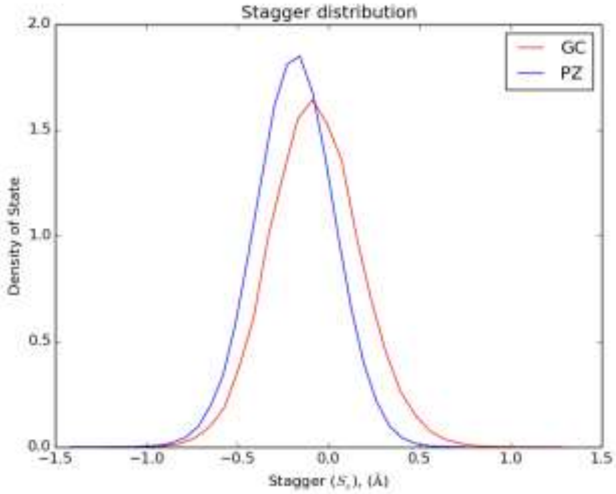
Stagger distribution
GC
PZ
Density of State

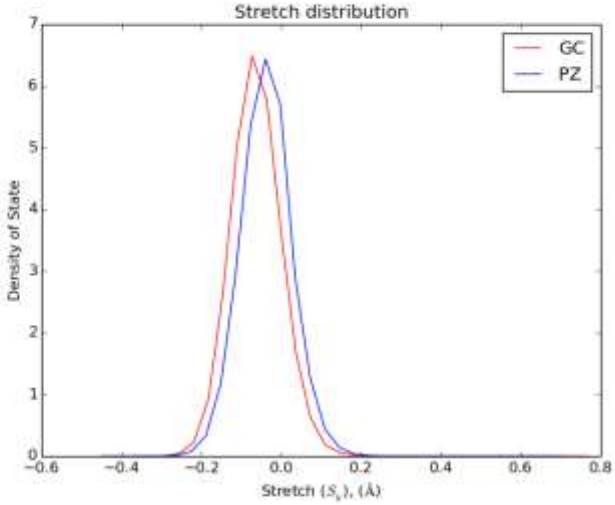
Stretch distribution
GC
PZ
Density of State

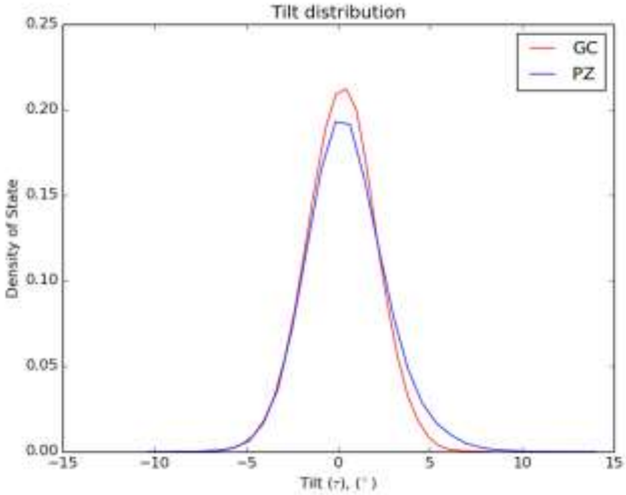
Tilt distribution
GC
PZ
Density of State

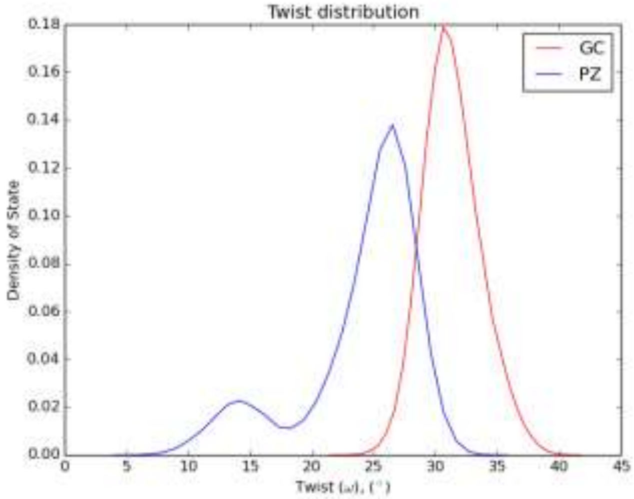
Twist distribution
GC
PZ
Density of State

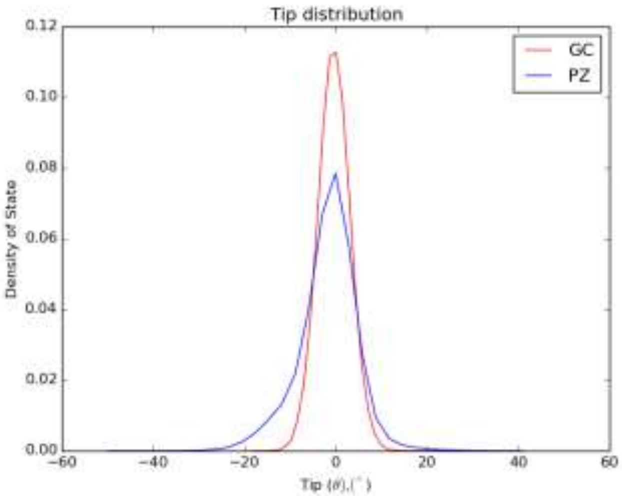
Tip distribution
Density of State
Tip (θ), (°)
GC
PZ

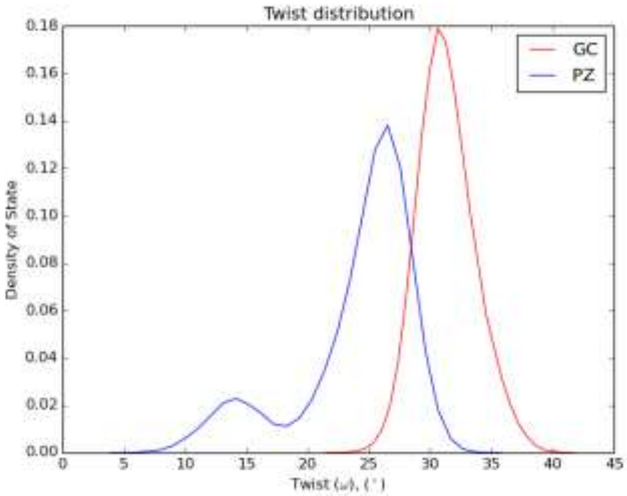
Twist distribution
Density of State
Twist (ω), (°)
GC
PZ

Figure S2. RMSD fluctuations over a 400 ns period in the MD trajectories computed for the (top) PZ and (bottom) GC duplexes. RMSD values are computed relative to an equilibrated structure at the beginning of the production part of the simulation. These data show that there is substantial interconversion between members of the two populations observed in the bimodal distributions for slide, twist, and h-twist in **PZ** (see main text). In each of these plots, values for the specified parameters in the **PZ** helix rapidly oscillate between two extreme values rather than staying in one extreme or the other for any given length of time. The tendency of the GC-rich duplex to adopt similar conformations is also evident in these data.

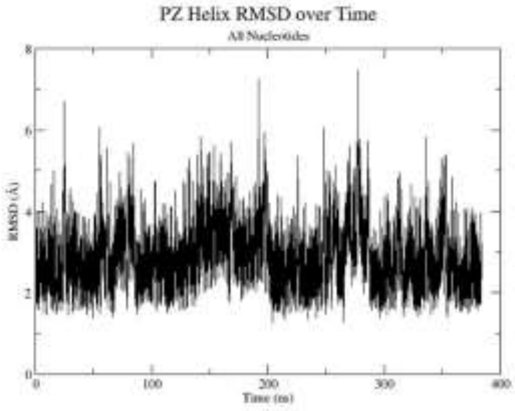

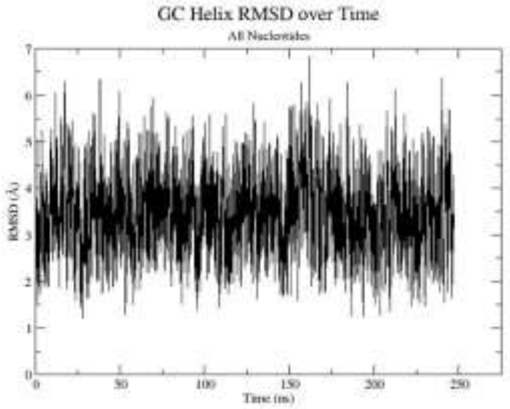

Figure S3. Representative PZ and GC structures from MD simulations. Stick models are shown for ZP in an extended conformation (far left), ZP in an A-like conformation (center), and GC in a B-like conformation. Views are shown parallel (upper panels) and perpendicular (lower panels) to the helical axes.

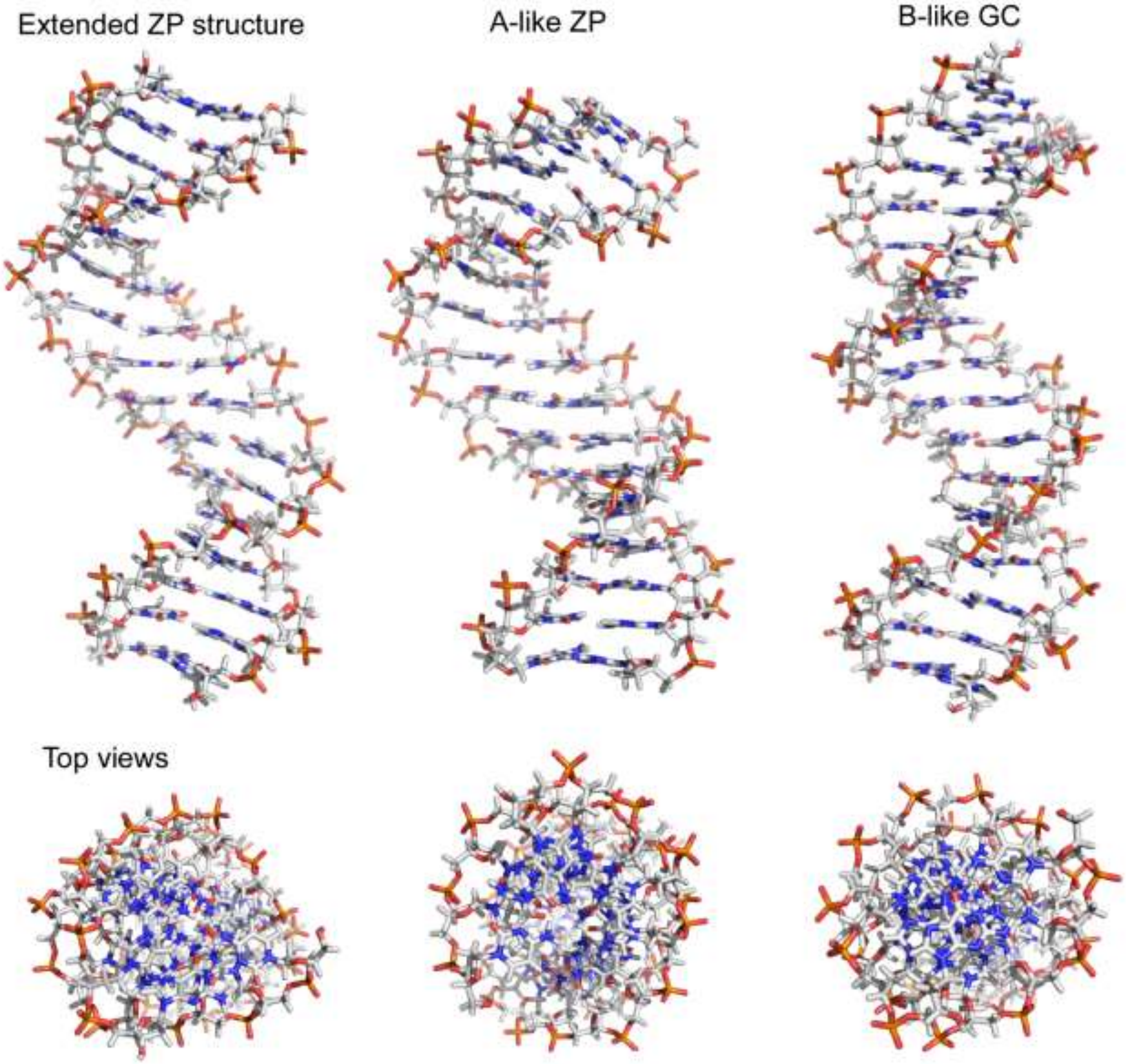

AMBER Parameters for the Z Nucleobase

```
MASS
CA 12.010        0.360              same as c2
C  12.010        0.616              same as c
HA 1.008         0.135              same as hc
CT 12.010        0.878              same as c3
H1 1.008         0.135              same as hc
HC 1.008         0.135              same as hc
OS 16.000        0.465              same as os
P  30.970        1.538              same as p4
O2 16.000        0.434              same as o
NO 14.100        0.530              Pulled from GAFF atom types
OD 16.000        0.434              Made up atom type for nitro oxygens; GAFF o
parameters
O  16.000        0.434              same as o
N2 14.010        0.530              same as n3
H  1.008         0.161              same as hn
NA 14.010        0.530              same as na
OH 16.000        0.465              same as oh
HO 1.008         0.135              same as ho

BOND
CA-C   449.90   1.406       same as c -c2
CA-CA  478.40   1.387       same as ca-ca
CA-CT  328.30   1.508       same as c2-c3
C -O   648.00   1.214       same as c -o
C -NA  478.20   1.345       Pulled from GAFF c-n parameters
CA-N2  449.00   1.364       same as ca-nh
CA-NA  411.10   1.391       same as c2-na
CA-NO  367.40   1.426       Pulled from GAFF cd-no parameters
CA-HA  344.30   1.087       same as c2-hc
CT-H1  337.30   1.092       same as c3-hc
CT-CT  303.10   1.535       same as c3-c3
CT-OS  301.50   1.439       same as c3-os
CT-HC  337.30   1.092       same as c3-hc
OS-P   311.60   1.636       same as os-p4
P -O2  456.40   1.503       same as o -p4
NO-OD  761.20   1.219       Pulled from GAFF no-o parameters
N2-H   394.10   1.018       same as hn-n3
NA-H   406.60   1.011       same as hn-na
P -OH  307.40   1.641       same as oh-p4
OH-HO  369.60   0.974       same as ho-oh

ANGLE
CA-C -O    72.770     119.120   same as c2-c -o
CA-C -NA   70.190     111.860   Pulled from cc-c-n parameters from GAFF
CA-CA-CA   67.180     119.970   same as ca-ca-ca
CA-CA-HA   50.300     119.700   same as c2-c2-hc
CA-CT-H1   47.030     110.490   same as c2-c3-hc
CA-CT-CT   63.530     111.440   same as c2-c3-c3
CA-CT-OS   68.450     108.480   same as c2-c3-os
C -CA-CA   67.930     120.700   same as c -c2-c2
C -CA-CT   63.870     119.700   same as c -c2-c3
C -NA-CA   65.240     124.190   Pulled from c-n-cc parameters from GAFF
C -NA-H    49.210     118.460   Pulled from c-n-hn parameters from GAFF
CA-CA-NO   65.780     128.950   Pulled from cc-cd-no parameters from GAFF
CA-N2-H    49.110     119.380   same as c2-n3-hn
CA-NA-H    47.620     119.280   same as c2-na-hn
CA-CA-N2   69.340     120.130   same as ca-ca-nh
CA-CA-NA   69.830     121.380   same as c2-c2-na
CA-NO-OD   70.340     117.520   Pulled cd-no-o parameters from GAFF
CA-CA-CT   64.330     123.420   same as c2-c2-c3
```

```
CT-CT-HC   46.370     110.050   same as c3-c3-hc
CT-CT-CT   63.210     110.630   same as c3-c3-c3
CT-OS-CT   62.390     112.450   same as c3-os-c3
H1-CT-CT   46.370     110.050   same as c3-c3-hc
H1-CT-OS   50.870     108.700   same as hc-c3-os
CT-CT-OS   67.780     108.420   same as c3-c3-os
HC-CT-HC   39.430     108.350   same as hc-c3-hc
CT-OS-P    77.590     117.480   same as c3-os-p4
OS-P -O2   43.100     116.670   same as o -p4-os
OS-P -OH   72.236      98.025   Calculated with empirical approach
H1-CT-H1   39.430     108.350   same as hc-c3-hc
OS-P -OS   44.740     100.340   same as os-p4-os
O2-P -O2   45.060     117.220   same as o -p4-o
OD-NO-OD   77.150     125.130   Pulled from o-no-o parameters from GAFF
O -C -NA   75.830     122.030   Pulled from o-c-n parameters from GAFF
N2-CA-NA   73.455     113.900   Calculated with empirical approach
H -N2-H    41.300     107.130   same as hn-n3-hn
P -OH-HO   55.270     110.190   same as ho-oh-p4
O2-P -OH   42.880     117.390   same as o -p4-oh

DIHE
CA-C -NA-CA   1    1.450       180.000          -2.000      same as X -c -na-X
CA-C -NA-CA   1    0.350       180.000           4.000      same as X -c -na-X
CA-C -NA-H    1    1.450       180.000          -2.000      same as X -c -na-X
CA-C -NA-H    1    0.350       180.000           4.000      same as X -c -na-X
CA-CA-CA-CA   1    3.625       180.000           2.000      same as X -ca-ca-X
CA-CA-CA-NO   1    4.000       180.000           2.000      Pulled from cc-cd-cd-no
GAFF param
CA-CT-CT-HC   1    0.156         0.000           3.000      same as X -c3-c3-X
CA-CT-CT-CT   1    0.156         0.000           3.000      same as X -c3-c3-X
CA-CT-OS-CT   1    0.383         0.000           3.000      same as X -c3-os-X
C -CA-CA-CA   1    6.650       180.000           2.000      same as X -c2-c2-X
C -CA-CA-HA   1    6.650       180.000           2.000      same as X -c2-c2-X
C -CA-CT-H1   1    0.000         0.000           2.000      same as X -c2-c3-X
C -CA-CT-CT   1    0.000         0.000           2.000      same as X -c2-c3-X
C -CA-CT-OS   1    0.000         0.000           2.000      same as X -c2-c3-X
C -NA-CA-CA   1    0.625       180.000           2.000      same as X -c2-na-X
C -NA-CA-N2   1    0.625       180.000           2.000      same as X -c2-na-X
CA-CA-CA-HA   1    6.650       180.000           2.000      same as X -c2-c2-X
CA-CA-NO-OD   1    0.750       180.000           2.000      Pulled from cc-cd-no-o
GAFF param
CA-NA-C -O    1    1.450       180.000          -2.000      same as X -c -na-X
CA-NA-C -O    1    0.350       180.000           4.000      same as X -c -na-X
CA-CA-N2-H    1    0.300       180.000           2.000      same as X -c2-n3-X
CA-CA-NA-H    1    0.625       180.000           2.000      same as X -c2-na-X
CA-CA-CA-CT   1    6.650       180.000           2.000      same as X -c2-c2-X
CA-CA-C -O    1    2.175       180.000          -2.000      same as c2-c2-c -o
CA-CA-C -O    1    0.300         0.000           3.000      same as c2-c2-c -o
CA-CA-C -NA   1    2.175       180.000           2.000      same as X -c -c2-X
CA-CA-CT-H1   1    0.380       180.000          -3.000      same as hc-c3-c2-c2
CA-CA-CT-H1   1    1.150         0.000           1.000      same as hc-c3-c2-c2
CA-CA-CT-CT   1    0.000         0.000           2.000      same as X -c2-c3-X
CA-CA-CT-OS   1    0.000         0.000           2.000      same as X -c2-c3-X
CA-CA-CA-N2   1    3.625       180.000           2.000      same as X -ca-ca-X
CA-CA-CA-NA   1    6.650       180.000           2.000      same as X -c2-c2-X
HA-CA-CA-CT   1    6.650       180.000           2.000      same as X -c2-c2-X
HA-CA-CA-NO   1    4.000       180.000           2.000      Pulled from ha-cd-cd-no
GAFF param
CT-CA-C -O    1    2.175       180.000           2.000      same as X -c -c2-X
CT-CA-C -NA   1    2.175       180.000           2.000      same as X -c -c2-X
CT-CT-CT-H1   1    0.160         0.000           3.000      same as hc-c3-c3-c3
CT-CT-CT-CT   1    0.180         0.000          -3.000      same as c3-c3-c3-c3
```

```
CT-CT-CT-CT   1    0.250       180.000           -2.000      same as c3-c3-c3-c3
CT-CT-CT-CT   1    0.200       180.000            1.000      same as c3-c3-c3-c3
CT-CT-CT-OS   1    0.156         0.000            3.000      same as X -c3-c3-X
CT-OS-CT-CT   1    0.383         0.000           -3.000      same as c3-c3-os-c3
CT-OS-CT-CT   1    0.100       180.000            2.000      same as c3-c3-os-c3
CT-OS-CT-H1   1    0.383         0.000            3.000      same as X -c3-os-X
H1-CT-CT-HC   1    0.150         0.000            3.000      same as hc-c3-c3-hc
CT-CT-OS-P    1    0.383         0.000            3.000      same as X -c3-os-X
HC-CT-CT-OS   1    0.000         0.000           -3.000      same as hc-c3-c3-os
HC-CT-CT-OS   1    0.250         0.000            1.000      same as hc-c3-c3-os
HC-CT-CT-CT   1    0.160         0.000            3.000      same as hc-c3-c3-c3
CT-OS-P -O2   1    1.050       180.000            2.000      same as X -os-p4-X
CT-OS-P -OH   1    1.050       180.000            2.000      same as X -os-p4-X
H1-CT-CT-H1   1    0.150         0.000            3.000      same as hc-c3-c3-hc
H1-CT-CT-OS   1    0.000         0.000           -3.000      same as hc-c3-c3-os
H1-CT-CT-OS   1    0.250         0.000            1.000      same as hc-c3-c3-os
H1-CT-OS-P    1    0.383         0.000            3.000      same as X -c3-os-X
OS-CT-CT-OS   1    0.144         0.000           -3.000      same as os-c3-c3-os
OS-CT-CT-OS   1    1.175         0.000            2.000      same as os-c3-c3-os
OS-P -OH-HO   1    0.700         0.000            1.000      same as X -oh-p4-X
CT-OS-P -OS   1    1.050       180.000            2.000      same as X -os-p4-X
NO-CA-CA-N2   1    4.000       180.000            2.000      Pulled from no-cd-cc-nh
GAFF param
NO-CA-CA-NA   1    4.000       180.000            2.000      Pulled from no-cd-cc-n
GAFF param
O -C -NA-H    1    1.450       180.000           -2.000      same as X -c -na-X
O -C -NA-H    1    0.350       180.000            4.000      same as X -c -na-X
N2-CA-NA-H    1    0.625       180.000            2.000      same as X -c2-na-X
H -N2-CA-NA   1    0.300       180.000            2.000      same as X -c2-n3-X
O2-P -OH-HO   1    0.700         0.000            1.000      same as X -oh-p4-X

IMPROPER
C -CA-CA-CT          5.6           180.0         2.0         Using default value
CA-NA-C -O           5.6           180.0         2.0         Using default value
CA-N2-CA-NA          5.6           180.0         2.0         Using default value
CA-CA-CA-NO          5.6           180.0         2.0         Using default value
CA-CA-CA-HA          5.6           180.0         2.0         Using default value
CA-H -N2-H           5.6           180.0         2.0         Using default value
C -CA-NA-H           5.6           180.0         2.0         Using default value

NONBON
  CA          1.9080  0.0860             same as ca
  C           1.9080  0.0860             same as c
  HA          1.4870  0.0157             same as hc
  CT          1.9080  0.1094             same as c3
  H1          1.4870  0.0157             same as hc
  HC          1.4870  0.0157             same as hc
  OS          1.6837  0.1700             same as os
  P           2.1000  0.2000             same as p4
  O2          1.6612  0.2100             same as o
  NO          1.8240  0.1700             Pulled from no GAFF parameters
  OD          1.6612  0.2100             Pulled from o GAFF parameters
  O           1.6612  0.2100             same as o
  N2          1.8240  0.1700             same as nh
  H           0.6000  0.0157             same as hn
  NA          1.8240  0.1700             same as na
  OH          1.7210  0.2104             same as oh
  HO          0.0000  0.0000             same as ho
```

AMBER Parameters for the P Nucleobase

```
MASS
CQ 12.010        0.360              same as c2
CB 12.010        0.360              same as c2
C  12.010        0.616              same as c
CA 12.010        0.360              same as c2
H4 1.008         0.135              same as ha
NC 14.010        0.530              same as n2
N* 14.010        0.530              same as na
N2 14.010        0.530              same as n3
H  1.008         0.161              same as hn
O  16.000        0.434              same as o
CT 12.010        0.878              same as c3
H2 1.008         0.135              same as hc
HC 1.008         0.135              same as hc
H1 1.008         0.135              same as hc
OS 16.000        0.465              same as os
O2 16.000        0.434              same as o
P  30.970        1.538              same as p4
OH 16.000        0.465              same as oh
HO 1.008         0.135              same as ho

BOND
CQ-NC  431.60   1.376       same as cc-nc
CQ-N2  449.00   1.364       same as cc-nh
CB-NC  431.60   1.376       same as cc-nc
CB-N*  411.10   1.391       same as c2-na
C -NC  374.60   1.420       same as c -n2
C -N*  438.80   1.371       my particular choice
C -O   648.00   1.214       same as c -o
CA-H4  344.30   1.087       same as c2-ha
CA-CA  478.40   1.387       same as ca-ca
CA-N*  411.10   1.391       same as c2-na
N*-CT  334.70   1.456       same as c3-na
N2-H   394.10   1.018       same as hn-n3
CT-H2  337.30   1.092       same as c3-hc
CT-CT  303.10   1.535       same as c3-c3
CT-OS  301.50   1.439       same as c3-os
CT-HC  337.30   1.092       same as c3-hc
CT-H1  337.30   1.092       same as c3-hc
OS-P   311.60   1.636       same as os-p4
O2-P   456.40   1.503       same as o -p4
P -OH  307.40   1.641       same as oh-p4
OH-HO  369.60   0.974       same as ho-oh

ANGLE
CQ-NC-CB   70.500     104.340   same as cc-nc-cc
CQ-NC-C    66.220     120.970   same as c -n2-c2
CQ-N2-H    49.110     119.380   same as c2-n3-hn
CB-N*-C    68.940     109.900   Pulled from GAFF cd-na-cd parameter
CB-N*-CA   67.800     110.370   same as c2-na-c2
CB-N*-CT   64.230     117.200   same as c2-na-c3
C -N*-CA   65.240     124.190   Pulled from GAFF c-n-cc parameter
CA-CA-H4   50.040     120.940   same as c2-c2-ha
CA-CA-N*   69.830     121.380   same as c2-c2-na
H4-CA-N*   51.180     112.420   same as ha-c2-na
CA-N*-CT   64.230     117.200   same as c2-na-c3
NC-CQ-NC   69.470     125.580   same as nc-cc-nc
NC-CQ-N2   72.330     116.850   same as nc-cc-nh
NC-CB-N*   71.710     123.620   same as n2-c2-na
NC-C -N*   72.350     117.050   Pulled from GAFF nd-c-n parameter
NC-C -O    73.020     122.500   same as n2-c -o
```

```
N*-CB-N*   73.650     109.330   same as na-c2-na
N*-C -O    75.830     122.030   Pulled from GAFF n-c-o parameter
N*-CT-H2   49.900     109.500   same as hc-c3-na
N*-CT-CT   65.730     112.810   same as c3-c3-na
N*-CT-OS   71.270     109.060   same as na-c3-os
H -N2-H    41.300     107.130   same as hn-n3-hn
CT-CT-HC   46.370     110.050   same as c3-c3-hc
CT-CT-CT   63.210     110.630   same as c3-c3-c3
CT-OS-CT   62.390     112.450   same as c3-os-c3
H2-CT-CT   46.370     110.050   same as c3-c3-hc
H2-CT-OS   50.870     108.700   same as hc-c3-os
CT-CT-OS   67.780     108.420   same as c3-c3-os
CT-CT-H1   46.370     110.050   same as c3-c3-hc
HC-CT-HC   39.430     108.350   same as hc-c3-hc
CT-OS-P    77.590     117.480   same as c3-os-p4
H1-CT-OS   50.870     108.700   same as hc-c3-os
OS-P -OH   72.236      98.025   Calculated with empirical approach
OS-P -O2   43.100     116.670   same as o -p4-os
H1-CT-H1   39.430     108.350   same as hc-c3-hc
OS-P -OS   44.740     100.340   same as os-p4-os
O2-P -O2   45.060     117.220   same as o -p4-o
P -OH-HO   55.270     110.190   same as ho-oh-p4
OH-P -O2   42.880     117.390   same as o -p4-oh

DIHE
CQ-NC-CB-N*   1    4.150       180.000           2.000      same as X -c2-n2-X
CQ-NC-C -N*   1    4.150       180.000           2.000      same as X -c -n2-X
CQ-NC-C -O    1    4.150       180.000           2.000      same as X -c -n2-X
CB-NC-CQ-NC   1    4.750       180.000           2.000      same as X -cc-nc-X
CB-NC-CQ-N2   1    4.750       180.000           2.000      same as X -cc-nc-X
CB-N*-C -NC   1    1.450       180.000          -2.000      same as X -c -na-X
CB-N*-C -NC   1    0.350       180.000           4.000      same as X -c -na-X
CB-N*-C -O    1    1.450       180.000          -2.000      same as X -c -na-X
CB-N*-C -O    1    0.350       180.000           4.000      same as X -c -na-X
CB-N*-CA-H4   1    0.625       180.000           2.000      same as X -c2-na-X
CB-N*-CA-CA   1    0.625       180.000           2.000      same as X -c2-na-X
CB-N*-CT-H2   1    0.000         0.000           2.000      same as X -c3-na-X
CB-N*-CT-CT   1    0.000         0.000           2.000      same as X -c3-na-X
CB-N*-CT-OS   1    0.000         0.000          -2.000      same as os-c3-na-c2
CB-N*-CT-OS   1    2.500         0.000           1.000      same as os-c3-na-c2
C -NC-CQ-NC   1    4.150       180.000           2.000      same as X -c2-n2-X
C -NC-CQ-N2   1    4.150       180.000           2.000      same as X -c2-n2-X
C -N*-CB-NC   1    0.625       180.000           2.000      same as X -c2-na-X
C -N*-CB-N*   1    0.625       180.000           2.000      same as X -c2-na-X
C -N*-CA-H4   1    0.625       180.000           2.000      same as X -c2-na-X
C -N*-CA-CA   1    0.625       180.000           2.000      same as X -c2-na-X
CA-CA-N*-CT   1    0.625       180.000           2.000      same as X -c2-na-X
CA-N*-CB-NC   1    0.625       180.000           2.000      same as X -c2-na-X
CA-N*-CB-N*   1    0.625       180.000           2.000      same as X -c2-na-X
CA-N*-C -NC   1    1.450       180.000          -2.000      same as X -c -na-X
CA-N*-C -NC   1    0.350       180.000           4.000      same as X -c -na-X
CA-N*-C -O    1    1.450       180.000          -2.000      same as X -c -na-X
CA-N*-C -O    1    0.350       180.000           4.000      same as X -c -na-X
H4-CA-CA-H4   1    3.625       180.000           2.000      same as X -ca-ca-X
H4-CA-CA-N*   1    6.650       180.000           2.000      same as X -c2-c2-X
CA-N*-CT-H2   1    0.000         0.000           2.000      same as X -c3-na-X
CA-N*-CT-CT   1    0.000         0.000           2.000      same as X -c3-na-X
CA-N*-CT-OS   1    0.000         0.000          -2.000      same as os-c3-na-c2
CA-N*-CT-OS   1    2.500         0.000           1.000      same as os-c3-na-c2
H4-CA-N*-CT   1    0.625       180.000           2.000      same as X -c2-na-X
NC-CQ-N2-H    1    0.300       180.000           2.000      same as X -c2-n3-X
NC-CB-N*-CT   1    0.625       180.000           2.000      same as X -c2-na-X
```

```
N*-CB-N*-CT   1    0.625        180.000            2.000       same as X -c2-na-X
N*-CA-CA-N*   1    6.650        180.000            2.000       same as X -c2-c2-X
N*-CT-CT-HC   1    0.156          0.000            3.000       same as X -c3-c3-X
N*-CT-CT-CT   1    0.156          0.000            3.000       same as X -c3-c3-X
N*-CT-OS-CT   1    0.383          0.000           -3.000       same as c3-os-c3-na
N*-CT-OS-CT   1    0.650          0.000            2.000       same as c3-os-c3-na
CT-CT-CT-H1   1    0.160          0.000            3.000       same as hc-c3-c3-c3
CT-CT-CT-CT   1    0.180          0.000           -3.000       same as c3-c3-c3-c3
CT-CT-CT-CT   1    0.250        180.000           -2.000       same as c3-c3-c3-c3
CT-CT-CT-CT   1    0.200        180.000            1.000       same as c3-c3-c3-c3
CT-CT-CT-OS   1    0.156          0.000            3.000       same as X -c3-c3-X
CT-OS-CT-CT   1    0.383          0.000           -3.000       same as c3-c3-os-c3
CT-OS-CT-CT   1    0.100        180.000            2.000       same as c3-c3-os-c3
CT-OS-CT-H1   1    0.383          0.000            3.000       same as X -c3-os-X
H2-CT-CT-HC   1    0.150          0.000            3.000       same as hc-c3-c3-hc
H2-CT-CT-CT   1    0.160          0.000            3.000       same as hc-c3-c3-c3
H2-CT-OS-CT   1    0.383          0.000            3.000       same as X -c3-os-X
CT-CT-OS-P    1    0.383          0.000            3.000       same as X -c3-os-X
HC-CT-CT-OS   1    0.000          0.000           -3.000       same as hc-c3-c3-os
HC-CT-CT-OS   1    0.250          0.000            1.000       same as hc-c3-c3-os
HC-CT-CT-H1   1    0.150          0.000            3.000       same as hc-c3-c3-hc
HC-CT-CT-CT   1    0.160          0.000            3.000       same as hc-c3-c3-c3
CT-OS-P -OH   1    1.050        180.000            2.000       same as X -os-p4-X
CT-OS-P -O2   1    1.050        180.000            2.000       same as X -os-p4-X
H1-CT-CT-H1   1    0.150          0.000            3.000       same as hc-c3-c3-hc
H1-CT-CT-OS   1    0.000          0.000           -3.000       same as hc-c3-c3-os
H1-CT-CT-OS   1    0.250          0.000            1.000       same as hc-c3-c3-os
H1-CT-OS-P    1    0.383          0.000            3.000       same as X -c3-os-X
OS-CT-CT-OS   1    0.144          0.000           -3.000       same as os-c3-c3-os
OS-CT-CT-OS   1    1.175          0.000            2.000       same as os-c3-c3-os
OS-P -OH-HO   1    0.700          0.000            1.000       same as X -oh-p4-X
CT-OS-P -OS   1    1.050        180.000            2.000       same as X -os-p4-X
HO-OH-P -O2   1    0.700          0.000            1.000       same as X -oh-p4-X

IMPROPER
N2-NC-CQ-NC         5.6           180.0         2.0          Using default value
N*-NC-C -O          5.6           180.0         2.0          Using default value
CA-H4-CA-N*         5.6           180.0         2.0          Using default value
C -CA-N*-CB         5.6           180.0         2.0          Using default value
CA-CB-N*-CT         5.6           180.0         2.0          Using default value
CQ-H -N2-H          5.6           180.0         2.0          Using default value

NONBON
  CQ          1.9080  0.0860             same as cc
  CB          1.9080  0.0860             same as cc
  C           1.9080  0.0860             same as c
  CA          1.9080  0.0860             same as ca
  H4          1.4870  0.0157             same as hc
  NC          1.8240  0.1700             same as nc
  N*          1.8240  0.1700             same as na
  N2          1.8240  0.1700             same as nh
  H           0.6000  0.0157             same as hn
  O           1.6612  0.2100             same as o
  CT          1.9080  0.1094             same as c3
  H2          1.4870  0.0157             same as hc
  HC          1.4870  0.0157             same as hc
  H1          1.4870  0.0157             same as hc
  OS          1.6837  0.1700             same as os
  O2          1.6612  0.2100             same as o
  P           2.1000  0.2000             same as p4
  OH          1.7210  0.2104             same as oh
  HO          0.0000  0.0000             same as ho
```

Coordinates of the gas-phase optimized structure of a P:Z base pair

C	3.556037	-5.347020	0.000000
H	4.260240	-4.520289	0.000000
N	2.219498	-4.786451	0.000000
C	1.018948	-5.493076	0.000000
H	1.008113	-6.567515	0.000000
C	-0.012368	-2.066098	0.000000
O	-1.231170	-1.998735	0.000000
N	0.845886	-1.040655	0.000000
C	2.175471	-1.257273	0.000000
N	2.958127	-0.183692	0.000000
H	2.549735	0.754435	0.000000
H	3.952174	-0.321980	0.000000
N	2.787840	-2.465246	0.000000
C	1.943178	-3.465961	0.000000
C	-2.253419	3.057766	0.000000
C	-1.347849	4.145428	0.000000
C	-0.003396	3.986968	0.000000
C	0.522669	2.643233	0.000000
C	-1.759312	1.744225	0.000000
H	-1.782369	5.135487	0.000000
H	3.707549	-5.954776	0.889919
H	3.707549	-5.954776	-0.889919
O	1.721846	2.365545	0.000000
N	-0.406910	1.614969	0.000000
H	0.000000	0.666484	0.000000
N	-2.472593	0.623909	0.000000
H	-3.474984	0.719809	0.000000
H	-2.022837	-0.290671	0.000000
C	0.006317	-4.609557	0.000000
H	-1.060731	-4.726530	0.000000
N	0.589987	-3.350080	0.000000
N	-3.646262	3.330824	0.000000
O	-4.438198	2.387242	0.000000
O	-4.011749	4.492556	0.000000
C	0.981968	5.111346	0.000000
H	1.631017	5.055818	-0.874609
H	0.469222	6.070890	0.000000
H	1.631017	5.055818	0.874609

Coordinates of the gas-phase optimized structure of a G:C base pair

C	-1.025032	-5.623477	0.000000
H	-1.648721	-5.509622	0.884596
N	0.028175	-4.633623	0.000000
C	1.391384	-4.837179	0.000000
H	1.799593	-5.835858	0.000000
N	2.078233	-3.736467	0.000000
C	1.125839	-2.741529	0.000000
C	1.242631	-1.320495	0.000000
O	2.256522	-0.628083	0.000000
N	-0.014757	-0.708730	0.000000
H	0.000000	0.320102	0.000000
C	-1.221674	-1.350315	0.000000
N	-2.312530	-0.564564	0.000000
H	-2.256133	0.451173	0.000000
H	-3.203326	-1.025249	0.000000
N	-1.345141	-2.658255	0.000000
C	-0.151276	-3.283730	0.000000
H	-0.572235	-6.611744	0.000000
H	-1.648721	-5.509622	-0.884596

C	1.316695	2.875428	0.000000
C	1.370624	4.308442	0.000000
C	0.191821	4.960447	0.000000
C	-1.017662	2.895217	0.000000
H	2.307650	4.840837	0.000000
H	0.127613	6.039823	0.000000
O	-2.112555	2.340402	0.000000
N	-0.988830	4.298479	0.000000
N	0.157903	2.228840	0.000000
N	2.434949	2.155509	0.000000
H	2.389682	1.126068	0.000000
H	3.326391	2.615699	0.000000
C	-2.268642	4.991683	0.000000
H	-2.841449	4.710990	-0.880758
H	-2.841449	4.710990	0.880758
H	-2.084894	6.062588	0.000000

Coordinates of the gas-phase optimized structure of a P nucleobase

C	-2.626708	-1.704608	-0.000008
H	-1.917752	-2.527492	0.000180
N	-1.867840	-0.471772	0.000008
C	-2.378076	0.824382	-0.000331
H	-3.437458	1.003780	-0.000632
C	1.186555	1.327653	0.000530
O	1.416194	2.512318	-0.000354
N	2.061683	0.302816	-0.000085
C	1.622488	-0.949269	-0.000175
N	2.563250	-1.915281	-0.000066
H	3.529924	-1.647884	-0.000075
H	2.286369	-2.878393	0.000100
N	0.341297	-1.392758	0.000088
C	-0.518724	-0.401312	0.000104
H	-3.251451	-1.762793	0.889557
H	-3.251141	-1.762925	-0.889780
C	-1.342696	1.683145	0.000214
H	-1.286888	2.755348	0.000420
N	-0.192418	0.911550	0.000205

Coordinates of the gas-phase optimized structure of a P nucleobase

C	0.790830	-0.231673	0.000230
C	-0.276967	-1.171695	0.000214
C	-1.578813	-0.813725	0.000001
C	-1.911213	0.598615	-0.000171
C	0.508828	1.132727	0.000156
H	0.003295	-2.215883	0.000328
O	-3.025361	1.076666	-0.000158
N	-0.804410	1.470706	-0.000282
H	-1.066161	2.447130	0.000544
N	1.401839	2.129122	0.001091
H	2.379078	1.879344	-0.001009
H	1.104667	3.087557	-0.003837
N	2.128976	-0.711219	-0.000064
O	3.053241	0.104803	-0.000169
O	2.313162	-1.911526	-0.000255
C	-2.724827	-1.772697	0.000081
H	-3.356623	-1.613876	-0.874256
H	-2.367624	-2.799945	0.000407
H	-3.356831	-1.613453	0.874197

Coordinates of the gas-phase optimized structure of a G nucleobase

C	-0.004890	3.152616	0.000000
H	0.616994	3.267289	0.885258
N	-0.640366	1.853149	0.000000
C	-1.986037	1.564931	0.000000
H	-2.720822	2.354480	0.000000
N	-2.239261	0.290866	0.000000
C	-1.000551	-0.303379	0.000000
C	-0.623116	-1.687596	0.000000
O	-1.287307	-2.695609	0.000000
N	0.799878	-1.795119	0.000000
H	1.128739	-2.749690	0.000000
C	1.694457	-0.765009	0.000000
N	3.010976	-1.094617	0.000000
H	3.330462	-2.043473	0.000000
H	3.678117	-0.345677	0.000000
N	1.346998	0.490345	0.000000
C	0.000000	0.652877	0.000000
H	-0.778775	3.915651	0.000000
H	0.616994	3.267289	-0.885258

Coordinates of the gas-phase optimized structure of a C nucleobase

C	1.280266	-0.846519	0.000000
C	0.131913	-1.699031	0.000000
C	-1.065814	-1.075083	0.000000
C	0.000000	1.089484	0.000000
H	0.209748	-2.773638	0.000000
H	-2.001018	-1.618643	0.000000
O	-0.157256	2.292353	0.000000
N	-1.160599	0.268280	0.000000
N	1.210213	0.460922	0.000000
N	2.513847	-1.401569	0.000000
H	3.309949	-0.788709	0.000000
H	2.645915	-2.394277	0.000000
C	-2.442011	0.955463	0.000000
H	-2.519557	1.590940	0.879161
H	-2.519557	1.590940	-0.879161
H	-3.237782	0.215243	0.000000

Coordinates of the "shift" conformer for two stacked P:Z nucleobase pairs (Fig. 9 in the main text)

```
C    5.354   1.046   2.116
H    5.217   1.772   1.304
N    4.281   0.072   2.054
C    4.365  -1.310   2.241
H    5.318  -1.793   2.418
C    0.864  -0.734   1.679
O    0.255  -1.797   1.710
N    0.342   0.485   1.470
C    1.135   1.580   1.464
N    0.539   2.751   1.254
H   -0.475   2.799   1.092
H    1.117   3.576   1.214
N    2.484   1.603   1.660
C    2.982   0.403   1.854
C   -4.704  -0.446   1.101
C   -5.263   0.851   0.969
C   -4.506   1.986   0.958
C   -3.071   1.846   1.063
C   -3.312  -0.593   1.253
H   -6.348   0.904   0.880
H    5.348   1.575   3.079
H    6.308   0.520   1.993
O   -2.272   2.791   1.015
N   -2.579   0.557   1.228
H   -1.549   0.494   1.311
N   -2.643  -1.737   1.411
H   -3.184  -2.592   1.384
H   -1.624  -1.748   1.494
C    3.120  -1.831   2.130
H    2.737  -2.843   2.175
N    2.271  -0.757   1.890
N   -5.581  -1.571   1.105
O   -5.097  -2.698   1.228
O   -6.780  -1.374   0.992
C   -5.065   3.373   0.856
H   -4.661   3.901  -0.019
H   -6.160   3.345   0.780
H   -4.786   3.968   1.738
C    7.316   0.728  -0.990
H    6.870   1.728  -0.972
N    6.237  -0.240  -0.905
C    6.365  -1.628  -0.794
H    7.337  -2.094  -0.689
C    2.819  -1.113  -1.091
O    2.232  -2.182  -0.978
N    2.263   0.096  -1.272
C    3.034   1.205  -1.348
N    2.405   2.356  -1.570
H    1.377   2.379  -1.661
H    2.959   3.197  -1.613
N    4.389   1.254  -1.215
C    4.925   0.064  -1.057
C   -2.765  -0.929  -1.793
C   -3.328   0.353  -2.032
C   -2.583   1.494  -2.062
C   -1.159   1.384  -1.834
C   -1.379  -1.050  -1.570
H   -4.408   0.390  -2.182
H    7.996   0.609  -0.136
H    7.877   0.599  -1.926
```

O	-0.373	2.342	-1.835
N	-0.662	0.108	-1.605
H	0.356	0.065	-1.441
N	-0.705	-2.174	-1.326
H	-1.245	-3.027	-1.268
H	0.295	-2.160	-1.126
C	5.128	-2.172	-0.854
H	4.770	-3.193	-0.801
N	4.242	-1.113	-1.013
N	-3.630	-2.056	-1.722
O	-3.140	-3.173	-1.526
O	-4.831	-1.877	-1.850
C	-3.134	2.868	-2.288
H	-2.712	3.314	-3.201
H	-4.227	2.838	-2.378
H	-2.851	3.523	-1.450

Coordinates of the "slide" conformer for two stacked P:Z nucleobase pairs (Fig. 8 in the main text)

C	-7.26028100	0.98336400	-0.73886000
H	-6.93912900	1.78050100	-0.05920100
N	-6.08573400	0.21689600	-1.11256600
C	-6.06190600	-0.97577500	-1.84199400
H	-6.97998100	-1.48026200	-2.12028200
C	-2.58630300	-0.13435100	-1.45845600
O	-1.88770400	-1.02641100	-1.92336500
N	-2.16461400	1.00213400	-0.88215000
C	-3.05066600	1.87876700	-0.35958400
N	-2.54679200	2.98543600	0.18392200
H	-1.53232000	3.16815600	0.14883600
H	-3.19257900	3.66911800	0.54730300
N	-4.40505000	1.73731000	-0.33150900
C	-4.81079400	0.63996800	-0.93300500
C	2.91777600	0.91941700	-1.58626100
C	3.33192700	2.21066500	-1.15687400
C	2.47722800	3.11427800	-0.60306900
C	1.08949400	2.73447100	-0.44779000
C	1.56972200	0.53754500	-1.44005700
H	4.38847800	2.44773700	-1.28404700
H	-7.72776300	1.43305500	-1.62544500
H	-7.98532400	0.33291500	-0.23274800
O	0.21421000	3.46663100	0.03322800
N	0.73986600	1.46278200	-0.88076600
H	-0.26237800	1.23040800	-0.79414300
N	1.03053800	-0.63726900	-1.76950100
H	1.64471400	-1.32150300	-2.19226400
H	0.02379500	-0.79508400	-1.70866900
C	-4.77054300	-1.29979800	-2.08173100
H	-4.30398500	-2.13377200	-2.59132300
N	-4.00262100	-0.29322600	-1.50721200
N	3.88985900	0.03081000	-2.11763900
O	3.53513100	-1.09636600	-2.48340400
O	5.05086600	0.40597800	-2.18217100
C	2.87504100	4.46864000	-0.10434000
H	2.68926500	4.54297700	0.97812900
H	3.93836600	4.65886100	-0.29896000
H	2.27674900	5.25566600	-0.58583100
C	-5.43207100	-0.37974700	2.21993500
H	-5.19229800	0.66948500	2.00478400
N	-4.39733100	-1.21599000	1.64019400
C	-4.54486300	-2.47208000	1.04997500
H	-5.52223300	-2.92655000	0.93921600

C	-1.00514200	-1.87632200	0.90506100
O	-0.43977400	-2.84028700	0.40397900
N	-0.43203200	-0.74587400	1.34770900
C	-1.18180100	0.23154100	1.90997600
N	-0.53968600	1.33344800	2.28590500
H	0.48257800	1.40433000	2.18904100
H	-1.08210600	2.09360800	2.66632300
N	-2.52697900	0.19865300	2.12497000
C	-3.08002300	-0.89364500	1.64900300
C	4.56656100	-1.52471300	0.50951900
C	5.16394300	-0.31676200	0.95004900
C	4.44801200	0.69641600	1.51825800
C	3.01961800	0.52791000	1.65386000
C	3.17779300	-1.70842100	0.64939200
H	6.23971100	-0.22501000	0.80170100
H	-6.39256400	-0.64478400	1.76092700
H	-5.49207200	-0.52518700	3.30691400
O	2.25535500	1.39501900	2.10280800
N	2.48999200	-0.68487900	1.23329800
H	1.45885600	-0.75512800	1.29570000
N	2.47280400	-2.77631000	0.27300300
H	2.98486100	-3.51367400	-0.19371500
H	1.45316200	-2.78503700	0.33965900
C	-3.32044500	-2.91330000	0.67592200
H	-2.98061100	-3.81355700	0.17941700
N	-2.42060000	-1.92472500	1.05312300
N	5.39564900	-2.52189100	-0.08388300
O	4.87752300	-3.57236100	-0.47202000
O	6.59248300	-2.30441000	-0.17352100
C	5.03572200	1.99226100	1.98644000
H	4.85803000	2.13584700	3.06265700
H	6.11594400	2.02062700	1.79664400
H	4.55723400	2.84080100	1.47462200

Comment on X- and Y-displacement distributions for the ZP-containing oligonucleotide. Although we find very narrowly peaked distributions for X- and Y-displacement values of the ZP-containing oligonucleotide during the trajectory (shown below), we find structures that exhibit very large values resulting from definitions of the standard helix reference frame.

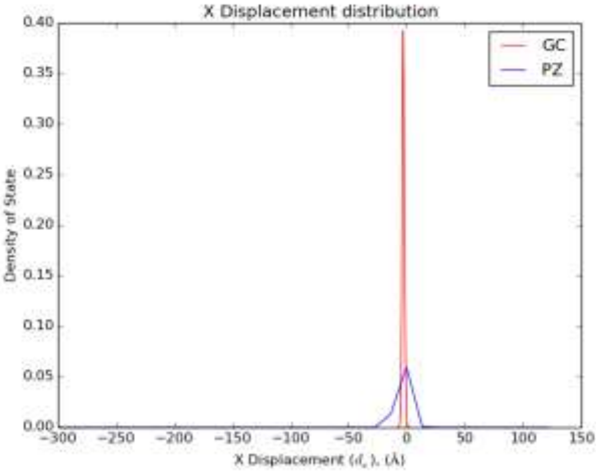

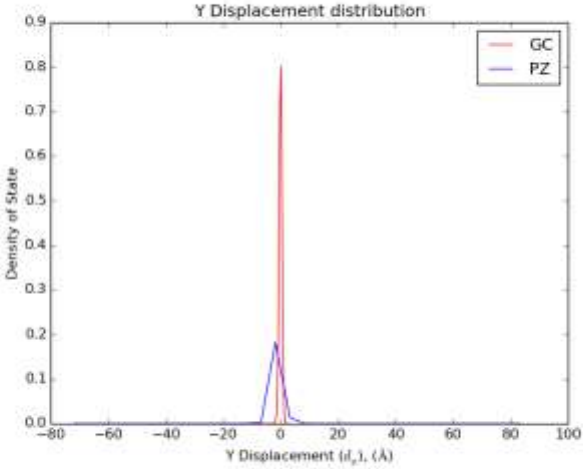

The following coordinates for one structure in which the X-displacement of the central dinucleotide step is measured to be 66 Å.

```
CRYST1   74.576   74.576   74.576 109.47 109.47 109.47               1
ATOM      1 HO5' DC5     1      44.506  51.685  20.062  1.00 0.00           H
ATOM      2  O5' DC5     1      43.892  51.944  20.772  1.00 0.00           O
ATOM      3  C5' DC5     1      44.362  53.131  21.389  1.00 0.00           C
ATOM      4  H5' DC5     1      44.426  53.779  20.515  1.00 0.00           H
ATOM      5 H5'' DC5     1      45.337  53.160  21.876  1.00 0.00           H
ATOM      6  C4' DC5     1      43.350  53.697  22.436  1.00 0.00           C
ATOM      7  H4' DC5     1      43.770  54.611  22.862  1.00 0.00           H
ATOM      8  O4' DC5     1      42.091  54.072  21.785  1.00 0.00           O
ATOM      9  C1' DC5     1      40.994  53.270  22.300  1.00 0.00           C
ATOM     10  H1' DC5     1      40.530  53.920  23.045  1.00 0.00           H
ATOM     11  N1  DC5     1      39.976  52.965  21.208  1.00 0.00           N
ATOM     12  C6  DC5     1      40.196  51.906  20.413  1.00 0.00           C
ATOM     13  H6  DC5     1      41.123  51.354  20.467  1.00 0.00           H
ATOM     14  C5  DC5     1      39.170  51.455  19.618  1.00 0.00           C
ATOM     15  H5  DC5     1      39.361  50.555  19.052  1.00 0.00           H
ATOM     16  C4  DC5     1      37.967  52.275  19.493  1.00 0.00           C
ATOM     17  N4  DC5     1      37.060  51.811  18.684  1.00 0.00           N
ATOM     18  H41 DC5     1      36.095  52.135  18.654  1.00 0.00           H
ATOM     19  H42 DC5     1      37.192  50.919  18.211  1.00 0.00           H
ATOM     20  N3  DC5     1      37.803  53.347  20.197  1.00 0.00           N
ATOM     21  C2  DC5     1      38.836  53.760  21.092  1.00 0.00           C
ATOM     22  O2  DC5     1      38.659  54.731  21.755  1.00 0.00           O
ATOM     23  C3' DC5     1      43.020  52.766  23.637  1.00 0.00           C
ATOM     24  H3' DC5     1      43.734  51.951  23.774  1.00 0.00           H
ATOM     25  C2' DC5     1      41.666  52.222  23.183  1.00 0.00           C
ATOM     26  H2' DC5     1      41.907  51.293  22.662  1.00 0.00           H
ATOM     27 H2'' DC5     1      41.071  51.827  24.009  1.00 0.00           H
ATOM     28  O3' DC5     1      42.884  53.615  24.855  1.00 0.00           O
ATOM     29  P    DT     2      42.686  52.928  26.295  1.00 0.00           P
ATOM     30  OP1  DT     2      43.522  53.441  27.355  1.00 0.00           O
ATOM     31  OP2  DT     2      42.678  51.376  26.220  1.00 0.00           O
ATOM     32  O5'  DT     2      41.147  53.472  26.581  1.00 0.00           O
ATOM     33  C5'  DT     2      40.886  54.868  26.897  1.00 0.00           C
ATOM     34  H5'  DT     2      41.274  55.502  26.100  1.00 0.00           H
ATOM     35 H5''  DT     2      41.409  55.196  27.795  1.00 0.00           H
ATOM     36  C4'  DT     2      39.403  55.154  26.934  1.00 0.00           C
```

ATOM	37	H4'	DT	2	39.201	56.188	27.222	1.00	0.00	H
ATOM	38	O4'	DT	2	38.763	54.836	25.648	1.00	0.00	O
ATOM	39	C1'	DT	2	37.570	54.030	25.855	1.00	0.00	C
ATOM	40	H1'	DT	2	36.801	54.794	25.996	1.00	0.00	H
ATOM	41	N1	DT	2	37.151	53.192	24.667	1.00	0.00	N
ATOM	42	C6	DT	2	37.796	52.051	24.308	1.00	0.00	C
ATOM	43	H6	DT	2	38.682	51.768	24.856	1.00	0.00	H
ATOM	44	C5	DT	2	37.373	51.310	23.220	1.00	0.00	C
ATOM	45	C7	DT	2	38.163	49.991	23.015	1.00	0.00	C
ATOM	46	H71	DT	2	37.736	49.237	23.678	1.00	0.00	H
ATOM	47	H72	DT	2	38.044	49.641	21.987	1.00	0.00	H
ATOM	48	H73	DT	2	39.227	50.122	23.220	1.00	0.00	H
ATOM	49	C4	DT	2	36.188	51.705	22.494	1.00	0.00	C
ATOM	50	O4	DT	2	35.644	51.197	21.536	1.00	0.00	O
ATOM	51	N3	DT	2	35.632	52.798	22.960	1.00	0.00	N
ATOM	52	H3	DT	2	34.868	53.173	22.414	1.00	0.00	H
ATOM	53	C2	DT	2	35.980	53.552	23.976	1.00	0.00	C
ATOM	54	O2	DT	2	35.338	54.536	24.167	1.00	0.00	O
ATOM	55	C3'	DT	2	38.692	54.237	28.015	1.00	0.00	C
ATOM	56	H3'	DT	2	39.414	53.778	28.694	1.00	0.00	H
ATOM	57	C2'	DT	2	37.834	53.258	27.159	1.00	0.00	C
ATOM	58	H2'	DT	2	38.261	52.276	26.944	1.00	0.00	H
ATOM	59	H2''	DT	2	36.894	53.039	27.670	1.00	0.00	H
ATOM	60	O3'	DT	2	37.746	55.006	28.754	1.00	0.00	O
ATOM	61	P	DT	3	37.129	54.389	30.171	1.00	0.00	P
ATOM	62	OP1	DT	3	37.426	55.292	31.367	1.00	0.00	O
ATOM	63	OP2	DT	3	37.549	53.003	30.506	1.00	0.00	O
ATOM	64	O5'	DT	3	35.501	54.467	29.824	1.00	0.00	O
ATOM	65	C5'	DT	3	34.935	55.630	29.330	1.00	0.00	C
ATOM	66	H5'	DT	3	35.573	56.320	28.777	1.00	0.00	H
ATOM	67	H5''	DT	3	34.725	56.274	30.184	1.00	0.00	H
ATOM	68	C4'	DT	3	33.700	55.469	28.462	1.00	0.00	C
ATOM	69	H4'	DT	3	33.401	56.460	28.116	1.00	0.00	H
ATOM	70	O4'	DT	3	34.048	54.572	27.312	1.00	0.00	O
ATOM	71	C1'	DT	3	32.877	53.780	26.972	1.00	0.00	C
ATOM	72	H1'	DT	3	32.215	54.500	26.487	1.00	0.00	H
ATOM	73	N1	DT	3	33.128	52.603	26.236	1.00	0.00	N
ATOM	74	C6	DT	3	34.315	51.829	26.448	1.00	0.00	C
ATOM	75	H6	DT	3	34.976	52.153	27.238	1.00	0.00	H
ATOM	76	C5	DT	3	34.460	50.645	25.777	1.00	0.00	C
ATOM	77	C7	DT	3	35.639	49.748	26.013	1.00	0.00	C
ATOM	78	H71	DT	3	36.210	49.512	25.112	1.00	0.00	H
ATOM	79	H72	DT	3	36.330	50.083	26.790	1.00	0.00	H
ATOM	80	H73	DT	3	35.126	48.827	26.297	1.00	0.00	H
ATOM	81	C4	DT	3	33.485	50.242	24.754	1.00	0.00	C
ATOM	82	O4	DT	3	33.471	49.121	24.236	1.00	0.00	O
ATOM	83	N3	DT	3	32.486	51.141	24.554	1.00	0.00	N
ATOM	84	H3	DT	3	31.798	50.933	23.843	1.00	0.00	H
ATOM	85	C2	DT	3	32.214	52.258	25.210	1.00	0.00	C
ATOM	86	O2	DT	3	31.100	52.807	25.004	1.00	0.00	O
ATOM	87	C3'	DT	3	32.598	54.686	29.312	1.00	0.00	C
ATOM	88	H3'	DT	3	32.988	54.419	30.296	1.00	0.00	H
ATOM	89	C2'	DT	3	32.205	53.535	28.349	1.00	0.00	C
ATOM	90	H2'	DT	3	32.599	52.646	28.847	1.00	0.00	H
ATOM	91	H2''	DT	3	31.122	53.415	28.286	1.00	0.00	H
ATOM	92	O3'	DT	3	31.354	55.518	29.446	1.00	0.00	O
ATOM	93	P	DA	4	30.147	55.286	30.564	1.00	0.00	P
ATOM	94	OP1	DA	4	29.570	56.570	30.965	1.00	0.00	O
ATOM	95	OP2	DA	4	30.820	54.761	31.785	1.00	0.00	O
ATOM	96	O5'	DA	4	29.137	54.335	29.692	1.00	0.00	O
ATOM	97	C5'	DA	4	28.335	54.778	28.571	1.00	0.00	C
ATOM	98	H5'	DA	4	29.027	55.079	27.784	1.00	0.00	H
ATOM	99	H5''	DA	4	27.651	55.566	28.884	1.00	0.00	H
ATOM	100	C4'	DA	4	27.473	53.648	28.224	1.00	0.00	C
ATOM	101	H4'	DA	4	26.670	54.038	27.595	1.00	0.00	H
ATOM	102	O4'	DA	4	28.263	52.726	27.378	1.00	0.00	O
ATOM	103	C1'	DA	4	27.704	51.388	27.431	1.00	0.00	C
ATOM	104	H1'	DA	4	26.847	51.374	26.754	1.00	0.00	H
ATOM	105	N9	DA	4	28.759	50.438	27.025	1.00	0.00	N

ATOM	106	C8	DA	4	30.109	50.511	27.275	1.00	0.00	C
ATOM	107	H8	DA	4	30.422	51.248	28.000	1.00	0.00	H
ATOM	108	N7	DA	4	30.881	49.560	26.797	1.00	0.00	N
ATOM	109	C5	DA	4	29.925	48.807	26.026	1.00	0.00	C
ATOM	110	C6	DA	4	30.006	47.748	25.044	1.00	0.00	C
ATOM	111	N6	DA	4	31.150	47.175	24.685	1.00	0.00	N
ATOM	112	H61	DA	4	31.187	46.571	23.867	1.00	0.00	H
ATOM	113	H62	DA	4	32.056	47.446	25.061	1.00	0.00	H
ATOM	114	N1	DA	4	28.954	47.258	24.420	1.00	0.00	N
ATOM	115	C2	DA	4	27.686	47.786	24.721	1.00	0.00	C
ATOM	116	H2	DA	4	26.862	47.325	24.197	1.00	0.00	H
ATOM	117	N3	DA	4	27.489	48.878	25.530	1.00	0.00	N
ATOM	118	C4	DA	4	28.636	49.376	26.048	1.00	0.00	C
ATOM	119	C3'	DA	4	26.815	52.749	29.358	1.00	0.00	C
ATOM	120	H3'	DA	4	27.161	53.106	30.330	1.00	0.00	H
ATOM	121	C2'	DA	4	27.274	51.286	28.898	1.00	0.00	C
ATOM	122	H2'	DA	4	28.124	50.842	29.422	1.00	0.00	H
ATOM	123	H2''	DA	4	26.414	50.613	28.928	1.00	0.00	H
ATOM	124	O3'	DA	4	25.346	52.858	29.333	1.00	0.00	O
ATOM	125	P	DT	5	24.384	52.481	30.632	1.00	0.00	P
ATOM	126	OP1	DT	5	23.239	53.463	30.659	1.00	0.00	O
ATOM	127	OP2	DT	5	25.115	52.543	31.928	1.00	0.00	O
ATOM	128	O5'	DT	5	24.125	51.036	30.092	1.00	0.00	O
ATOM	129	C5'	DT	5	23.280	50.869	29.058	1.00	0.00	C
ATOM	130	H5'	DT	5	23.890	51.330	28.282	1.00	0.00	H
ATOM	131	H5''	DT	5	22.288	51.280	29.243	1.00	0.00	H
ATOM	132	C4'	DT	5	23.018	49.337	28.874	1.00	0.00	C
ATOM	133	H4'	DT	5	22.129	49.200	28.256	1.00	0.00	H
ATOM	134	O4'	DT	5	24.171	48.811	28.225	1.00	0.00	O
ATOM	135	C1'	DT	5	24.606	47.553	28.906	1.00	0.00	C
ATOM	136	H1'	DT	5	24.042	46.745	28.435	1.00	0.00	H
ATOM	137	N1	DT	5	26.020	47.225	28.563	1.00	0.00	N
ATOM	138	C6	DT	5	27.062	47.645	29.422	1.00	0.00	C
ATOM	139	H6	DT	5	26.792	48.280	30.253	1.00	0.00	H
ATOM	140	C5	DT	5	28.359	47.310	29.179	1.00	0.00	C
ATOM	141	C7	DT	5	29.519	47.914	30.039	1.00	0.00	C
ATOM	142	H71	DT	5	30.039	47.096	30.543	1.00	0.00	H
ATOM	143	H72	DT	5	30.373	48.356	29.521	1.00	0.00	H
ATOM	144	H73	DT	5	29.049	48.532	30.806	1.00	0.00	H
ATOM	145	C4	DT	5	28.630	46.391	28.095	1.00	0.00	C
ATOM	146	O4	DT	5	29.759	46.071	27.736	1.00	0.00	O
ATOM	147	N3	DT	5	27.573	45.894	27.489	1.00	0.00	N
ATOM	148	H3	DT	5	27.830	45.268	26.739	1.00	0.00	H
ATOM	149	C2	DT	5	26.300	46.248	27.596	1.00	0.00	C
ATOM	150	O2	DT	5	25.522	45.654	26.872	1.00	0.00	O
ATOM	151	C3'	DT	5	22.787	48.478	30.132	1.00	0.00	C
ATOM	152	H3'	DT	5	22.470	49.127	30.950	1.00	0.00	H
ATOM	153	C2'	DT	5	24.146	47.821	30.351	1.00	0.00	C
ATOM	154	H2'	DT	5	24.823	48.485	30.892	1.00	0.00	H
ATOM	155	H2''	DT	5	24.090	46.932	30.983	1.00	0.00	H
ATOM	156	O3'	DT	5	21.942	47.442	29.805	1.00	0.00	O
ATOM	157	P	1WA	6	20.789	47.076	30.806	1.00	0.00	P
ATOM	158	OP1	1WA	6	19.591	46.383	30.197	1.00	0.00	O
ATOM	159	OP2	1WA	6	20.270	48.319	31.561	1.00	0.00	O
ATOM	160	O5'	1WA	6	21.578	45.963	31.819	1.00	0.00	O
ATOM	161	C5'	1WA	6	21.541	44.548	31.463	1.00	0.00	C
ATOM	162	H10	1WA	6	20.501	44.241	31.331	1.00	0.00	H
ATOM	163	H11	1WA	6	21.975	44.042	32.328	1.00	0.00	H
ATOM	164	C4'	1WA	6	22.283	44.155	30.196	1.00	0.00	C
ATOM	165	H9	1WA	6	21.908	44.727	29.345	1.00	0.00	H
ATOM	166	O4'	1WA	6	23.619	44.443	30.236	1.00	0.00	O
ATOM	167	C1'	1WA	6	24.291	43.657	29.249	1.00	0.00	C
ATOM	168	N9	1WA	6	25.714	43.588	29.545	1.00	0.00	N
ATOM	169	C4	1WA	6	26.648	42.937	28.782	1.00	0.00	C
ATOM	170	N3	1WA	6	26.457	42.111	27.715	1.00	0.00	N
ATOM	171	C2	1WA	6	27.640	41.599	27.298	1.00	0.00	C
ATOM	172	N1	1WA	6	28.907	41.849	27.760	1.00	0.00	N
ATOM	173	C6	1WA	6	28.949	42.712	28.863	1.00	0.00	C
ATOM	174	N5	1WA	6	27.848	43.318	29.336	1.00	0.00	N

```
ATOM    175  C7  1WA     6      27.651  44.033  30.494  1.00 0.00           C
ATOM    176  H1  1WA     6      28.489  44.394  31.085  1.00 0.00           H
ATOM    177  C8  1WA     6      26.314  44.205  30.690  1.00 0.00           C
ATOM    178  H2  1WA     6      25.777  44.660  31.519  1.00 0.00           H
ATOM    179  O6  1WA     6      30.043  42.945  29.331  1.00 0.00           O
ATOM    180  N2  1WA     6      27.525  40.783  26.326  1.00 0.00           N
ATOM    181  H3  1WA     6      26.611  40.673  25.891  1.00 0.00           H
ATOM    182  H4  1WA     6      28.240  40.119  26.034  1.00 0.00           H
ATOM    183  H5  1WA     6      24.021  44.167  28.321  1.00 0.00           H
ATOM    184  C2' 1WA     6      23.591  42.300  29.226  1.00 0.00           C
ATOM    185  H6  1WA     6      24.094  41.564  29.858  1.00 0.00           H
ATOM    186  H7  1WA     6      23.524  41.825  28.245  1.00 0.00           H
ATOM    187  C3' 1WA     6      22.225  42.666  29.862  1.00 0.00           C
ATOM    188  H8  1WA     6      21.385  42.469  29.194  1.00 0.00           H
ATOM    189  O3' 1WA     6      22.162  41.836  31.089  1.00 0.00           O
ATOM    190  P   1WA     7      21.193  40.506  31.058  1.00 0.00           P
ATOM    191  OP1 1WA     7      19.905  40.801  30.274  1.00 0.00           O
ATOM    192  OP2 1WA     7      20.728  39.908  32.340  1.00 0.00           O
ATOM    193  O5' 1WA     7      22.125  39.481  30.199  1.00 0.00           O
ATOM    194  C5' 1WA     7      23.297  38.935  30.788  1.00 0.00           C
ATOM    195  H10 1WA     7      23.016  38.275  31.611  1.00 0.00           H
ATOM    196  H11 1WA     7      23.892  39.712  31.274  1.00 0.00           H
ATOM    197  C4' 1WA     7      24.108  38.000  29.883  1.00 0.00           C
ATOM    198  H9  1WA     7      23.511  37.418  29.178  1.00 0.00           H
ATOM    199  O4' 1WA     7      25.084  38.790  29.143  1.00 0.00           O
ATOM    200  C1' 1WA     7      26.309  38.080  29.027  1.00 0.00           C
ATOM    201  N9  1WA     7      27.404  38.817  29.660  1.00 0.00           N
ATOM    202  C4  1WA     7      28.776  38.398  29.569  1.00 0.00           C
ATOM    203  N3  1WA     7      29.293  37.277  28.861  1.00 0.00           N
ATOM    204  C2  1WA     7      30.642  37.276  29.042  1.00 0.00           C
ATOM    205  N1  1WA     7      31.373  38.008  29.812  1.00 0.00           N
ATOM    206  C6  1WA     7      30.883  39.047  30.553  1.00 0.00           C
ATOM    207  N5  1WA     7      29.538  39.292  30.415  1.00 0.00           N
ATOM    208  C7  1WA     7      28.647  40.119  31.103  1.00 0.00           C
ATOM    209  H1  1WA     7      28.918  40.813  31.894  1.00 0.00           H
ATOM    210  C8  1WA     7      27.349  39.834  30.595  1.00 0.00           C
ATOM    211  H2  1WA     7      26.418  40.352  30.808  1.00 0.00           H
ATOM    212  O6  1WA     7      31.646  39.768  31.117  1.00 0.00           O
ATOM    213  N2  1WA     7      31.330  36.394  28.296  1.00 0.00           N
ATOM    214  H3  1WA     7      32.330  36.552  28.184  1.00 0.00           H
ATOM    215  H4  1WA     7      30.839  35.720  27.713  1.00 0.00           H
ATOM    216  H5  1WA     7      26.556  37.919  27.976  1.00 0.00           H
ATOM    217  C2' 1WA     7      26.084  36.713  29.798  1.00 0.00           C
ATOM    218  H6  1WA     7      27.024  36.415  30.269  1.00 0.00           H
ATOM    219  H7  1WA     7      25.763  36.024  29.014  1.00 0.00           H
ATOM    220  C3' 1WA     7      24.882  36.970  30.738  1.00 0.00           C
ATOM    221  H8  1WA     7      24.282  36.066  30.860  1.00 0.00           H
ATOM    222  O3' 1WA     7      25.346  37.591  31.984  1.00 0.00           O
ATOM    223  P   1WA     8      25.999  36.741  33.155  1.00 0.00           P
ATOM    224  OP1 1WA     8      25.286  35.403  33.239  1.00 0.00           O
ATOM    225  OP2 1WA     8      25.949  37.656  34.321  1.00 0.00           O
ATOM    226  O5' 1WA     8      27.548  36.563  32.733  1.00 0.00           O
ATOM    227  C5' 1WA     8      28.279  35.379  33.127  1.00 0.00           C
ATOM    228  H10 1WA     8      27.736  34.496  32.784  1.00 0.00           H
ATOM    229  H11 1WA     8      28.305  35.377  34.218  1.00 0.00           H
ATOM    230  C4' 1WA     8      29.626  35.437  32.435  1.00 0.00           C
ATOM    231  H9  1WA     8      29.465  35.596  31.366  1.00 0.00           H
ATOM    232  O4' 1WA     8      30.492  36.450  32.999  1.00 0.00           O
ATOM    233  C1' 1WA     8      31.819  36.174  32.493  1.00 0.00           C
ATOM    234  N9  1WA     8      32.828  36.912  33.228  1.00 0.00           N
ATOM    235  C4  1WA     8      34.219  36.723  33.163  1.00 0.00           C
ATOM    236  N3  1WA     8      34.898  35.804  32.296  1.00 0.00           N
ATOM    237  C2  1WA     8      36.200  35.950  32.500  1.00 0.00           C
ATOM    238  N1  1WA     8      36.884  36.794  33.308  1.00 0.00           N
ATOM    239  C6  1WA     8      36.123  37.722  34.123  1.00 0.00           C
ATOM    240  N5  1WA     8      34.803  37.752  33.969  1.00 0.00           N
ATOM    241  C7  1WA     8      33.759  38.492  34.541  1.00 0.00           C
ATOM    242  H1  1WA     8      33.858  39.359  35.189  1.00 0.00           H
ATOM    243  C8  1WA     8      32.570  38.032  34.025  1.00 0.00           C
```

ATOM	244	H2	1WA	8	31.553	38.403	34.117	1.00	0.00	H
ATOM	245	O6	1WA	8	36.802	38.494	34.833	1.00	0.00	O
ATOM	246	N2	1WA	8	36.880	35.037	31.814	1.00	0.00	N
ATOM	247	H3	1WA	8	37.863	34.826	31.971	1.00	0.00	H
ATOM	248	H4	1WA	8	36.447	34.447	31.106	1.00	0.00	H
ATOM	249	H5	1WA	8	31.949	36.247	31.411	1.00	0.00	H
ATOM	250	C2'	1WA	8	31.962	34.699	32.673	1.00	0.00	C
ATOM	251	H6	1WA	8	32.362	34.456	33.659	1.00	0.00	H
ATOM	252	H7	1WA	8	32.595	34.143	31.978	1.00	0.00	H
ATOM	253	C3'	1WA	8	30.480	34.188	32.626	1.00	0.00	C
ATOM	254	H8	1WA	8	30.293	33.451	31.842	1.00	0.00	H
ATOM	255	O3'	1WA	8	30.185	33.639	33.952	1.00	0.00	O
ATOM	256	P	1W5	9	30.381	32.027	34.264	1.00	0.00	P
ATOM	257	OP1	1W5	9	29.329	31.113	33.684	1.00	0.00	O
ATOM	258	OP2	1W5	9	30.481	31.756	35.741	1.00	0.00	O
ATOM	259	O5'	1W5	9	31.669	31.682	33.374	1.00	0.00	O
ATOM	260	C5'	1W5	9	32.310	30.424	33.538	1.00	0.00	C
ATOM	261	H7	1W5	9	32.378	30.317	34.623	1.00	0.00	H
ATOM	262	H8	1W5	9	31.756	29.596	33.091	1.00	0.00	H
ATOM	263	C4'	1W5	9	33.687	30.406	32.735	1.00	0.00	C
ATOM	264	H6	1W5	9	33.217	30.224	31.766	1.00	0.00	H
ATOM	265	O4'	1W5	9	34.317	31.735	32.935	1.00	0.00	O
ATOM	266	C1'	1W5	9	35.627	31.508	33.476	1.00	0.00	C
ATOM	267	C1	1W5	9	36.162	32.615	34.319	1.00	0.00	C
ATOM	268	C2	1W5	9	37.546	32.863	34.365	1.00	0.00	C
ATOM	269	O2	1W5	9	38.355	32.168	33.780	1.00	0.00	O
ATOM	270	N3	1W5	9	37.902	33.811	35.206	1.00	0.00	N
ATOM	271	C4	1W5	9	37.051	34.544	35.911	1.00	0.00	C
ATOM	272	C5	1W5	9	35.716	34.407	35.887	1.00	0.00	C
ATOM	273	C6	1W5	9	35.205	33.349	35.045	1.00	0.00	C
ATOM	274	H1	1W5	9	34.144	33.125	34.968	1.00	0.00	H
ATOM	275	N5	1W5	9	34.827	35.345	36.542	1.00	0.00	N
ATOM	276	ON1	1W5	9	33.694	35.429	36.082	1.00	0.00	O
ATOM	277	ON2	1W5	9	35.231	35.986	37.497	1.00	0.00	O
ATOM	278	N4	1W5	9	37.683	35.451	36.661	1.00	0.00	N
ATOM	279	H9	1W5	9	38.692	35.419	36.791	1.00	0.00	H
ATOM	280	H10	1W5	9	37.204	36.311	36.917	1.00	0.00	H
ATOM	281	H11	1W5	9	38.890	34.019	35.166	1.00	0.00	H
ATOM	282	H2	1W5	9	36.270	31.393	32.600	1.00	0.00	H
ATOM	283	C2'	1W5	9	35.589	30.124	34.143	1.00	0.00	C
ATOM	284	H3	1W5	9	36.575	29.658	34.202	1.00	0.00	H
ATOM	285	H4	1W5	9	35.213	30.118	35.168	1.00	0.00	H
ATOM	286	C3'	1W5	9	34.619	29.302	33.290	1.00	0.00	C
ATOM	287	H5	1W5	9	34.055	28.584	33.889	1.00	0.00	H
ATOM	288	O3'	1W5	9	35.208	28.568	32.111	1.00	0.00	O
ATOM	289	P	1W5	10	35.772	27.076	32.326	1.00	0.00	P
ATOM	290	OP1	1W5	10	35.873	26.048	31.199	1.00	0.00	O
ATOM	291	OP2	1W5	10	34.870	26.390	33.256	1.00	0.00	O
ATOM	292	O5'	1W5	10	37.234	27.514	32.823	1.00	0.00	O
ATOM	293	C5'	1W5	10	37.937	26.628	33.700	1.00	0.00	C
ATOM	294	H7	1W5	10	37.606	26.755	34.733	1.00	0.00	H
ATOM	295	H8	1W5	10	37.688	25.608	33.401	1.00	0.00	H
ATOM	296	C4'	1W5	10	39.476	26.859	33.627	1.00	0.00	C
ATOM	297	H6	1W5	10	39.780	26.851	32.579	1.00	0.00	H
ATOM	298	O4'	1W5	10	39.689	28.184	34.204	1.00	0.00	O
ATOM	299	C1'	1W5	10	40.732	28.210	35.246	1.00	0.00	C
ATOM	300	C1	1W5	10	40.289	29.341	36.156	1.00	0.00	C
ATOM	301	C2	1W5	10	41.244	30.248	36.664	1.00	0.00	C
ATOM	302	O2	1W5	10	42.416	29.999	36.550	1.00	0.00	O
ATOM	303	N3	1W5	10	40.844	31.318	37.301	1.00	0.00	N
ATOM	304	C4	1W5	10	39.506	31.599	37.461	1.00	0.00	C
ATOM	305	C5	1W5	10	38.455	30.714	37.106	1.00	0.00	C
ATOM	306	C6	1W5	10	38.876	29.615	36.386	1.00	0.00	C
ATOM	307	H1	1W5	10	38.182	28.915	35.926	1.00	0.00	H
ATOM	308	N5	1W5	10	37.012	30.839	37.380	1.00	0.00	N
ATOM	309	ON1	1W5	10	36.355	29.841	37.441	1.00	0.00	O
ATOM	310	ON2	1W5	10	36.673	31.889	37.850	1.00	0.00	O
ATOM	311	N4	1W5	10	39.338	32.742	38.061	1.00	0.00	N
ATOM	312	H9	1W5	10	40.074	33.183	38.608	1.00	0.00	H

```
ATOM    313  H10 1W5    10      38.526  33.340  38.203  1.00 0.00           H
ATOM    314  H11 1W5    10      41.565  31.895  37.711  1.00 0.00           H
ATOM    315  H2  1W5    10      41.753  28.248  34.863  1.00 0.00           H
ATOM    316  C2' 1W5    10      40.573  26.843  35.815  1.00 0.00           C
ATOM    317  H3  1W5    10      41.357  26.496  36.491  1.00 0.00           H
ATOM    318  H4  1W5    10      39.702  26.799  36.473  1.00 0.00           H
ATOM    319  C3' 1W5    10      40.203  25.898  34.593  1.00 0.00           C
ATOM    320  H5  1W5    10      39.536  25.085  34.887  1.00 0.00           H
ATOM    321  O3' 1W5    10      41.420  25.486  34.050  1.00 0.00           O
ATOM    322  P   1W5    11      41.959  23.972  34.485  1.00 0.00           P
ATOM    323  OP1 1W5    11      42.366  23.256  33.269  1.00 0.00           O
ATOM    324  OP2 1W5    11      41.015  23.095  35.292  1.00 0.00           O
ATOM    325  O5' 1W5    11      43.205  24.442  35.366  1.00 0.00           O
ATOM    326  C5' 1W5    11      43.121  24.053  36.756  1.00 0.00           C
ATOM    327  H7  1W5    11      42.126  24.193  37.183  1.00 0.00           H
ATOM    328  H8  1W5    11      43.322  22.980  36.794  1.00 0.00           H
ATOM    329  C4' 1W5    11      44.199  24.786  37.585  1.00 0.00           C
ATOM    330  H6  1W5    11      45.053  24.916  36.917  1.00 0.00           H
ATOM    331  O4' 1W5    11      43.761  26.091  37.908  1.00 0.00           O
ATOM    332  C1' 1W5    11      44.288  26.439  39.207  1.00 0.00           C
ATOM    333  C1  1W5    11      43.384  27.513  39.795  1.00 0.00           C
ATOM    334  C2  1W5    11      44.008  28.649  40.405  1.00 0.00           C
ATOM    335  O2  1W5    11      45.234  28.720  40.448  1.00 0.00           O
ATOM    336  N3  1W5    11      43.207  29.546  41.022  1.00 0.00           N
ATOM    337  C4  1W5    11      41.830  29.500  40.844  1.00 0.00           C
ATOM    338  C5  1W5    11      41.215  28.436  40.173  1.00 0.00           C
ATOM    339  C6  1W5    11      42.007  27.544  39.618  1.00 0.00           C
ATOM    340  H1  1W5    11      41.516  26.669  39.199  1.00 0.00           H
ATOM    341  N5  1W5    11      39.782  28.483  40.102  1.00 0.00           N
ATOM    342  ON1 1W5    11      39.154  28.407  41.166  1.00 0.00           O
ATOM    343  ON2 1W5    11      39.257  28.116  39.062  1.00 0.00           O
ATOM    344  N4  1W5    11      41.205  30.543  41.352  1.00 0.00           N
ATOM    345  H9  1W5    11      41.832  31.216  41.788  1.00 0.00           H
ATOM    346  H10 1W5    11      40.234  30.822  41.236  1.00 0.00           H
ATOM    347  H11 1W5    11      43.592  30.319  41.546  1.00 0.00           H
ATOM    348  H2  1W5    11      45.269  26.831  38.930  1.00 0.00           H
ATOM    349  C2' 1W5    11      44.335  25.123  39.981  1.00 0.00           C
ATOM    350  H3  1W5    11      45.000  25.215  40.842  1.00 0.00           H
ATOM    351  H4  1W5    11      43.387  24.807  40.421  1.00 0.00           H
ATOM    352  C3' 1W5    11      44.681  24.045  38.873  1.00 0.00           C
ATOM    353  H5  1W5    11      44.199  23.091  39.094  1.00 0.00           H
ATOM    354  O3' 1W5    11      46.099  23.974  38.657  1.00 0.00           O
ATOM    355  P    DA    12      47.020  22.972  39.549  1.00 0.00           P
ATOM    356  OP1  DA    12      48.299  22.590  38.868  1.00 0.00           O
ATOM    357  OP2  DA    12      46.393  21.681  40.090  1.00 0.00           O
ATOM    358  O5'  DA    12      47.266  24.042  40.687  1.00 0.00           O
ATOM    359  C5'  DA    12      48.369  24.900  40.791  1.00 0.00           C
ATOM    360  H5'  DA    12      48.477  25.564  39.934  1.00 0.00           H
ATOM    361 H5''  DA    12      49.330  24.388  40.846  1.00 0.00           H
ATOM    362  C4'  DA    12      48.310  25.793  42.054  1.00 0.00           C
ATOM    363  H4'  DA    12      49.131  26.505  41.948  1.00 0.00           H
ATOM    364  O4'  DA    12      47.119  26.543  42.238  1.00 0.00           O
ATOM    365  C1'  DA    12      46.989  26.962  43.671  1.00 0.00           C
ATOM    366  H1'  DA    12      47.681  27.761  43.942  1.00 0.00           H
ATOM    367  N9   DA    12      45.672  27.290  43.981  1.00 0.00           N
ATOM    368  C8   DA    12      44.486  26.915  43.408  1.00 0.00           C
ATOM    369  H8   DA    12      44.484  26.129  42.667  1.00 0.00           H
ATOM    370  N7   DA    12      43.401  27.546  43.718  1.00 0.00           N
ATOM    371  C5   DA    12      43.956  28.548  44.586  1.00 0.00           C
ATOM    372  C6   DA    12      43.415  29.646  45.264  1.00 0.00           C
ATOM    373  N6   DA    12      42.187  30.074  45.193  1.00 0.00           N
ATOM    374  H61  DA    12      41.823  30.917  45.632  1.00 0.00           H
ATOM    375  H62  DA    12      41.521  29.569  44.611  1.00 0.00           H
ATOM    376  N1   DA    12      44.196  30.482  45.951  1.00 0.00           N
ATOM    377  C2   DA    12      45.540  30.204  46.083  1.00 0.00           C
ATOM    378  H2   DA    12      46.099  30.883  46.710  1.00 0.00           H
ATOM    379  N3   DA    12      46.198  29.191  45.371  1.00 0.00           N
ATOM    380  C4   DA    12      45.324  28.401  44.691  1.00 0.00           C
ATOM    381  C3'  DA    12      48.581  25.107  43.425  1.00 0.00           C
```

ATOM	382	H3'	DA	12	48.544	24.016	43.412	1.00	0.00	H
ATOM	383	C2'	DA	12	47.590	25.743	44.339	1.00	0.00	C
ATOM	384	H2'	DA	12	46.739	25.074	44.489	1.00	0.00	H
ATOM	385	H2''	DA	12	48.016	26.032	45.302	1.00	0.00	H
ATOM	386	O3'	DA	12	49.862	25.594	43.969	1.00	0.00	O
ATOM	387	P	DT	13	50.729	24.822	45.029	1.00	0.00	P
ATOM	388	OP1	DT	13	52.119	24.906	44.680	1.00	0.00	O
ATOM	389	OP2	DT	13	50.129	23.418	45.163	1.00	0.00	O
ATOM	390	O5'	DT	13	50.236	25.809	46.187	1.00	0.00	O
ATOM	391	C5'	DT	13	50.757	27.147	46.401	1.00	0.00	C
ATOM	392	H5'	DT	13	50.608	27.891	45.618	1.00	0.00	H
ATOM	393	H5''	DT	13	51.806	27.112	46.697	1.00	0.00	H
ATOM	394	C4'	DT	13	49.993	27.716	47.663	1.00	0.00	C
ATOM	395	H4'	DT	13	50.202	28.770	47.853	1.00	0.00	H
ATOM	396	O4'	DT	13	48.621	27.780	47.386	1.00	0.00	O
ATOM	397	C1'	DT	13	47.886	27.795	48.603	1.00	0.00	C
ATOM	398	H1'	DT	13	47.877	28.797	49.037	1.00	0.00	H
ATOM	399	N1	DT	13	46.536	27.365	48.486	1.00	0.00	N
ATOM	400	C6	DT	13	46.184	26.245	47.785	1.00	0.00	C
ATOM	401	H6	DT	13	46.938	25.504	47.567	1.00	0.00	H
ATOM	402	C5	DT	13	44.867	26.080	47.481	1.00	0.00	C
ATOM	403	C7	DT	13	44.579	24.873	46.649	1.00	0.00	C
ATOM	404	H71	DT	13	45.432	24.205	46.511	1.00	0.00	H
ATOM	405	H72	DT	13	43.754	24.253	47.007	1.00	0.00	H
ATOM	406	H73	DT	13	44.195	25.373	45.758	1.00	0.00	H
ATOM	407	C4	DT	13	43.813	27.030	47.762	1.00	0.00	C
ATOM	408	O4	DT	13	42.636	26.983	47.444	1.00	0.00	O
ATOM	409	N3	DT	13	44.294	28.071	48.486	1.00	0.00	N
ATOM	410	H3	DT	13	43.639	28.744	48.861	1.00	0.00	H
ATOM	411	C2	DT	13	45.583	28.299	48.751	1.00	0.00	C
ATOM	412	O2	DT	13	45.804	29.382	49.240	1.00	0.00	O
ATOM	413	C3'	DT	13	50.112	26.840	48.918	1.00	0.00	C
ATOM	414	H3'	DT	13	50.331	25.779	48.785	1.00	0.00	H
ATOM	415	C2'	DT	13	48.737	26.911	49.547	1.00	0.00	C
ATOM	416	H2'	DT	13	48.319	25.924	49.756	1.00	0.00	H
ATOM	417	H2''	DT	13	48.825	27.387	50.526	1.00	0.00	H
ATOM	418	O3'	DT	13	51.018	27.480	49.854	1.00	0.00	O
ATOM	419	P	DA	14	51.886	26.631	50.960	1.00	0.00	P
ATOM	420	OP1	DA	14	53.369	26.973	50.807	1.00	0.00	O
ATOM	421	OP2	DA	14	51.740	25.168	50.710	1.00	0.00	O
ATOM	422	O5'	DA	14	51.265	27.059	52.411	1.00	0.00	O
ATOM	423	C5'	DA	14	51.540	28.352	52.852	1.00	0.00	C
ATOM	424	H5'	DA	14	51.408	29.137	52.108	1.00	0.00	H
ATOM	425	H5''	DA	14	52.555	28.371	53.250	1.00	0.00	H
ATOM	426	C4'	DA	14	50.620	28.838	53.968	1.00	0.00	C
ATOM	427	H4'	DA	14	50.966	29.833	54.254	1.00	0.00	H
ATOM	428	O4'	DA	14	49.181	28.807	53.623	1.00	0.00	O
ATOM	429	C1'	DA	14	48.467	27.788	54.368	1.00	0.00	C
ATOM	430	H1'	DA	14	47.838	28.325	55.080	1.00	0.00	H
ATOM	431	N9	DA	14	47.624	27.096	53.309	1.00	0.00	N
ATOM	432	C8	DA	14	47.727	25.820	52.755	1.00	0.00	C
ATOM	433	H8	DA	14	48.587	25.214	53.000	1.00	0.00	H
ATOM	434	N7	DA	14	46.656	25.490	52.044	1.00	0.00	N
ATOM	435	C5	DA	14	45.873	26.613	52.076	1.00	0.00	C
ATOM	436	C6	DA	14	44.547	26.851	51.600	1.00	0.00	C
ATOM	437	N6	DA	14	43.863	25.960	50.853	1.00	0.00	N
ATOM	438	H61	DA	14	42.872	26.098	50.668	1.00	0.00	H
ATOM	439	H62	DA	14	44.302	25.081	50.585	1.00	0.00	H
ATOM	440	N1	DA	14	43.861	27.953	51.934	1.00	0.00	N
ATOM	441	C2	DA	14	44.507	28.843	52.766	1.00	0.00	C
ATOM	442	H2	DA	14	43.939	29.735	52.985	1.00	0.00	H
ATOM	443	N3	DA	14	45.821	28.777	53.272	1.00	0.00	N
ATOM	444	C4	DA	14	46.388	27.576	52.853	1.00	0.00	C
ATOM	445	C3'	DA	14	50.791	27.928	55.215	1.00	0.00	C
ATOM	446	H3'	DA	14	51.707	27.334	55.188	1.00	0.00	H
ATOM	447	C2'	DA	14	49.554	27.026	55.105	1.00	0.00	C
ATOM	448	H2'	DA	14	49.876	26.151	54.536	1.00	0.00	H
ATOM	449	H2''	DA	14	49.115	26.725	56.058	1.00	0.00	H
ATOM	450	O3'	DA	14	50.726	28.844	56.360	1.00	0.00	O

```
ATOM    451  P    DA    15      50.409  28.240  57.797  1.00 0.00           P
ATOM    452  OP1  DA    15      51.304  28.949  58.813  1.00 0.00           O
ATOM    453  OP2  DA    15      50.674  26.771  57.989  1.00 0.00           O
ATOM    454  O5'  DA    15      48.852  28.639  58.085  1.00 0.00           O
ATOM    455  C5'  DA    15      48.387  29.983  57.956  1.00 0.00           C
ATOM    456  H5'  DA    15      48.494  30.268  56.909  1.00 0.00           H
ATOM    457 H5''  DA    15      48.910  30.578  58.704  1.00 0.00           H
ATOM    458  C4'  DA    15      46.912  30.098  58.317  1.00 0.00           C
ATOM    459  H4'  DA    15      46.579  31.127  58.163  1.00 0.00           H
ATOM    460  O4'  DA    15      46.114  29.302  57.370  1.00 0.00           O
ATOM    461  C1'  DA    15      45.333  28.321  58.037  1.00 0.00           C
ATOM    462  H1'  DA    15      44.316  28.711  58.113  1.00 0.00           H
ATOM    463  N9   DA    15      45.397  27.139  57.152  1.00 0.00           N
ATOM    464  C8   DA    15      46.179  26.074  57.158  1.00 0.00           C
ATOM    465  H8   DA    15      47.048  25.977  57.792  1.00 0.00           H
ATOM    466  N7   DA    15      45.800  25.035  56.466  1.00 0.00           N
ATOM    467  C5   DA    15      44.612  25.524  55.893  1.00 0.00           C
ATOM    468  C6   DA    15      43.720  24.986  54.976  1.00 0.00           C
ATOM    469  N6   DA    15      43.733  23.809  54.384  1.00 0.00           N
ATOM    470  H61  DA    15      43.042  23.465  53.720  1.00 0.00           H
ATOM    471  H62  DA    15      44.472  23.110  54.331  1.00 0.00           H
ATOM    472  N1   DA    15      42.667  25.614  54.582  1.00 0.00           N
ATOM    473  C2   DA    15      42.473  26.948  54.982  1.00 0.00           C
ATOM    474  H2   DA    15      41.572  27.424  54.623  1.00 0.00           H
ATOM    475  N3   DA    15      43.322  27.649  55.866  1.00 0.00           N
ATOM    476  C4   DA    15      44.386  26.810  56.213  1.00 0.00           C
ATOM    477  C3'  DA    15      46.467  29.634  59.737  1.00 0.00           C
ATOM    478  H3'  DA    15      47.282  29.711  60.460  1.00 0.00           H
ATOM    479  C2'  DA    15      45.945  28.245  59.442  1.00 0.00           C
ATOM    480  H2'  DA    15      46.788  27.564  59.580  1.00 0.00           H
ATOM    481 H2''  DA    15      45.236  27.935  60.213  1.00 0.00           H
ATOM    482  O3'  DA    15      45.348  30.541  60.202  1.00 0.00           O
ATOM    483  P   DG3    16      44.753  30.650  61.679  1.00 0.00           P
ATOM    484  OP1 DG3    16      44.535  32.046  62.214  1.00 0.00           O
ATOM    485  OP2 DG3    16      45.590  29.838  62.600  1.00 0.00           O
ATOM    486  O5' DG3    16      43.307  30.045  61.365  1.00 0.00           O
ATOM    487  C5' DG3    16      42.412  30.814  60.605  1.00 0.00           C
ATOM    488  H5' DG3    16      42.806  31.041  59.614  1.00 0.00           H
ATOM    489 H5'' DG3    16      42.224  31.736  61.155  1.00 0.00           H
ATOM    490  C4' DG3    16      41.159  30.032  60.219  1.00 0.00           C
ATOM    491  H4' DG3    16      40.523  30.674  59.606  1.00 0.00           H
ATOM    492  O4' DG3    16      41.453  28.922  59.408  1.00 0.00           O
ATOM    493  C1' DG3    16      40.748  27.782  59.875  1.00 0.00           C
ATOM    494  H1' DG3    16      39.795  27.858  59.348  1.00 0.00           H
ATOM    495  N9  DG3    16      41.491  26.575  59.484  1.00 0.00           N
ATOM    496  C8  DG3    16      42.770  26.225  59.783  1.00 0.00           C
ATOM    497  H8  DG3    16      43.332  26.808  60.497  1.00 0.00           H
ATOM    498  N7  DG3    16      43.166  25.133  59.089  1.00 0.00           N
ATOM    499  C5  DG3    16      42.077  24.712  58.434  1.00 0.00           C
ATOM    500  C6  DG3    16      41.854  23.593  57.567  1.00 0.00           C
ATOM    501  O6  DG3    16      42.639  22.755  57.283  1.00 0.00           O
ATOM    502  N1  DG3    16      40.710  23.528  56.977  1.00 0.00           N
ATOM    503  H1  DG3    16      40.493  22.854  56.255  1.00 0.00           H
ATOM    504  C2  DG3    16      39.712  24.380  57.231  1.00 0.00           C
ATOM    505  N2  DG3    16      38.576  23.919  56.831  1.00 0.00           N
ATOM    506  H21 DG3    16      38.545  23.051  56.300  1.00 0.00           H
ATOM    507  H22 DG3    16      37.661  24.253  57.126  1.00 0.00           H
ATOM    508  N3  DG3    16      39.783  25.467  58.003  1.00 0.00           N
ATOM    509  C4  DG3    16      41.057  25.609  58.560  1.00 0.00           C
ATOM    510  C3' DG3    16      40.401  29.524  61.430  1.00 0.00           C
ATOM    511  H3' DG3    16      40.663  29.900  62.421  1.00 0.00           H
ATOM    512  C2' DG3    16      40.656  27.961  61.417  1.00 0.00           C
ATOM    513  H2' DG3    16      41.581  27.697  61.935  1.00 0.00           H
ATOM    514 H2'' DG3    16      39.786  27.540  61.926  1.00 0.00           H
ATOM    515  O3' DG3    16      39.044  29.748  61.267  1.00 0.00           O
ATOM    516 HO3' DG3    16      38.616  29.569  62.124  1.00 0.00           H
TER     517      DG3    16
ATOM    517 HO5' DC5    17      37.509  16.560  49.954  1.00 0.00           H
ATOM    518  O5' DC5    17      37.212  17.367  50.413  1.00 0.00           O
```

ATOM	519	C5'	DC5	17	36.174	16.939	51.191	1.00	0.00	C
ATOM	520	H5'	DC5	17	36.498	16.032	51.701	1.00	0.00	H
ATOM	521	H5''	DC5	17	35.388	16.614	50.509	1.00	0.00	H
ATOM	522	C4'	DC5	17	35.713	18.071	52.093	1.00	0.00	C
ATOM	523	H4'	DC5	17	34.814	17.727	52.609	1.00	0.00	H
ATOM	524	O4'	DC5	17	36.613	18.300	53.203	1.00	0.00	O
ATOM	525	C1'	DC5	17	36.931	19.787	53.372	1.00	0.00	C
ATOM	526	H1'	DC5	17	36.181	20.263	54.007	1.00	0.00	H
ATOM	527	N1	DC5	17	38.347	19.984	53.746	1.00	0.00	N
ATOM	528	C6	DC5	17	39.381	19.552	52.857	1.00	0.00	C
ATOM	529	H6	DC5	17	39.173	19.030	51.934	1.00	0.00	H
ATOM	530	C5	DC5	17	40.628	19.868	53.111	1.00	0.00	C
ATOM	531	H5	DC5	17	41.379	19.590	52.387	1.00	0.00	H
ATOM	532	C4	DC5	17	40.936	20.593	54.265	1.00	0.00	C
ATOM	533	N4	DC5	17	42.168	20.918	54.647	1.00	0.00	N
ATOM	534	H41	DC5	17	42.175	21.350	55.569	1.00	0.00	H
ATOM	535	H42	DC5	17	43.014	20.594	54.182	1.00	0.00	H
ATOM	536	N3	DC5	17	40.015	21.143	55.075	1.00	0.00	N
ATOM	537	C2	DC5	17	38.661	20.792	54.826	1.00	0.00	C
ATOM	538	O2	DC5	17	37.840	21.354	55.507	1.00	0.00	O
ATOM	539	C3'	DC5	17	35.479	19.457	51.367	1.00	0.00	C
ATOM	540	H3'	DC5	17	35.575	19.355	50.284	1.00	0.00	H
ATOM	541	C2'	DC5	17	36.576	20.334	51.959	1.00	0.00	C
ATOM	542	H2'	DC5	17	37.465	20.368	51.325	1.00	0.00	H
ATOM	543	H2''	DC5	17	36.170	21.347	51.984	1.00	0.00	H
ATOM	544	O3'	DC5	17	34.160	19.921	51.671	1.00	0.00	O
ATOM	545	P	DT	18	33.357	21.031	50.729	1.00	0.00	P
ATOM	546	OP1	DT	18	31.843	20.965	50.873	1.00	0.00	O
ATOM	547	OP2	DT	18	33.613	20.719	49.307	1.00	0.00	O
ATOM	548	O5'	DT	18	33.863	22.436	51.260	1.00	0.00	O
ATOM	549	C5'	DT	18	33.647	22.830	52.551	1.00	0.00	C
ATOM	550	H5'	DT	18	33.704	21.934	53.169	1.00	0.00	H
ATOM	551	H5''	DT	18	32.619	23.190	52.584	1.00	0.00	H
ATOM	552	C4'	DT	18	34.537	23.881	53.135	1.00	0.00	C
ATOM	553	H4'	DT	18	34.091	24.161	54.091	1.00	0.00	H
ATOM	554	O4'	DT	18	35.991	23.489	53.270	1.00	0.00	O
ATOM	555	C1'	DT	18	36.820	24.596	53.022	1.00	0.00	C
ATOM	556	H1'	DT	18	36.793	25.185	53.942	1.00	0.00	H
ATOM	557	N1	DT	18	38.227	24.230	52.759	1.00	0.00	N
ATOM	558	C6	DT	18	38.440	23.311	51.745	1.00	0.00	C
ATOM	559	H6	DT	18	37.579	23.047	51.150	1.00	0.00	H
ATOM	560	C5	DT	18	39.714	22.906	51.536	1.00	0.00	C
ATOM	561	C7	DT	18	39.904	21.985	50.323	1.00	0.00	C
ATOM	562	H71	DT	18	40.644	22.415	49.645	1.00	0.00	H
ATOM	563	H72	DT	18	40.188	20.997	50.690	1.00	0.00	H
ATOM	564	H73	DT	18	39.036	21.679	49.734	1.00	0.00	H
ATOM	565	C4	DT	18	40.856	23.429	52.282	1.00	0.00	C
ATOM	566	O4	DT	18	41.997	23.048	52.065	1.00	0.00	O
ATOM	567	N3	DT	18	40.497	24.240	53.247	1.00	0.00	N
ATOM	568	H3	DT	18	41.254	24.706	53.730	1.00	0.00	H
ATOM	569	C2	DT	18	39.285	24.756	53.483	1.00	0.00	C
ATOM	570	O2	DT	18	39.108	25.722	54.191	1.00	0.00	O
ATOM	571	C3'	DT	18	34.508	25.154	52.255	1.00	0.00	C
ATOM	572	H3'	DT	18	33.993	24.936	51.317	1.00	0.00	H
ATOM	573	C2'	DT	18	36.029	25.392	51.965	1.00	0.00	C
ATOM	574	H2'	DT	18	36.083	25.002	50.946	1.00	0.00	H
ATOM	575	H2''	DT	18	36.299	26.450	51.980	1.00	0.00	H
ATOM	576	O3'	DT	18	33.938	26.276	52.968	1.00	0.00	O
ATOM	577	P	DT	19	33.364	27.572	52.204	1.00	0.00	P
ATOM	578	OP1	DT	19	31.996	27.999	52.709	1.00	0.00	O
ATOM	579	OP2	DT	19	33.218	27.341	50.748	1.00	0.00	O
ATOM	580	O5'	DT	19	34.521	28.749	52.307	1.00	0.00	O
ATOM	581	C5'	DT	19	34.531	29.520	53.515	1.00	0.00	C
ATOM	582	H5'	DT	19	34.480	28.779	54.313	1.00	0.00	H
ATOM	583	H5''	DT	19	33.687	30.210	53.541	1.00	0.00	H
ATOM	584	C4'	DT	19	35.872	30.314	53.747	1.00	0.00	C
ATOM	585	H4'	DT	19	35.764	30.840	54.697	1.00	0.00	H
ATOM	586	O4'	DT	19	36.941	29.382	53.919	1.00	0.00	O
ATOM	587	C1'	DT	19	38.112	29.940	53.468	1.00	0.00	C

ATOM	588	H1'	DT	19	38.578	30.273	54.397	1.00	0.00	H
ATOM	589	N1	DT	19	38.926	28.985	52.640	1.00	0.00	N
ATOM	590	C6	DT	19	38.301	28.281	51.665	1.00	0.00	C
ATOM	591	H6	DT	19	37.250	28.347	51.423	1.00	0.00	H
ATOM	592	C5	DT	19	39.020	27.423	50.931	1.00	0.00	C
ATOM	593	C7	DT	19	38.264	26.662	49.838	1.00	0.00	C
ATOM	594	H71	DT	19	38.192	25.608	50.113	1.00	0.00	H
ATOM	595	H72	DT	19	37.329	27.193	49.649	1.00	0.00	H
ATOM	596	H73	DT	19	38.779	26.845	48.892	1.00	0.00	H
ATOM	597	C4	DT	19	40.440	27.225	51.169	1.00	0.00	C
ATOM	598	O4	DT	19	41.116	26.336	50.633	1.00	0.00	O
ATOM	599	N3	DT	19	40.941	28.011	52.083	1.00	0.00	N
ATOM	600	H3	DT	19	41.949	27.934	52.102	1.00	0.00	H
ATOM	601	C2	DT	19	40.292	28.907	52.829	1.00	0.00	C
ATOM	602	O2	DT	19	40.868	29.702	53.607	1.00	0.00	O
ATOM	603	C3'	DT	19	36.188	31.323	52.670	1.00	0.00	C
ATOM	604	H3'	DT	19	35.642	31.053	51.764	1.00	0.00	H
ATOM	605	C2'	DT	19	37.674	31.114	52.576	1.00	0.00	C
ATOM	606	H2'	DT	19	37.881	30.974	51.513	1.00	0.00	H
ATOM	607	H2''	DT	19	38.220	31.997	52.914	1.00	0.00	H
ATOM	608	O3'	DT	19	35.827	32.646	53.059	1.00	0.00	O
ATOM	609	P	DA	20	35.850	33.853	51.955	1.00	0.00	P
ATOM	610	OP1	DA	20	34.820	34.773	52.396	1.00	0.00	O
ATOM	611	OP2	DA	20	35.719	33.363	50.555	1.00	0.00	O
ATOM	612	O5'	DA	20	37.329	34.442	52.187	1.00	0.00	O
ATOM	613	C5'	DA	20	37.790	34.909	53.486	1.00	0.00	C
ATOM	614	H5'	DA	20	37.585	34.112	54.201	1.00	0.00	H
ATOM	615	H5''	DA	20	37.176	35.743	53.826	1.00	0.00	H
ATOM	616	C4'	DA	20	39.309	35.190	53.589	1.00	0.00	C
ATOM	617	H4'	DA	20	39.560	35.451	54.619	1.00	0.00	H
ATOM	618	O4'	DA	20	40.013	33.969	53.069	1.00	0.00	O
ATOM	619	C1'	DA	20	40.623	34.197	51.733	1.00	0.00	C
ATOM	620	H1'	DA	20	41.700	34.241	51.909	1.00	0.00	H
ATOM	621	N9	DA	20	40.222	33.155	50.851	1.00	0.00	N
ATOM	622	C8	DA	20	39.183	32.888	50.014	1.00	0.00	C
ATOM	623	H8	DA	20	38.294	33.502	50.042	1.00	0.00	H
ATOM	624	N7	DA	20	39.246	31.738	49.383	1.00	0.00	N
ATOM	625	C5	DA	20	40.530	31.257	49.744	1.00	0.00	C
ATOM	626	C6	DA	20	41.280	30.097	49.351	1.00	0.00	C
ATOM	627	N6	DA	20	40.976	29.025	48.601	1.00	0.00	N
ATOM	628	H61	DA	20	41.722	28.378	48.354	1.00	0.00	H
ATOM	629	H62	DA	20	40.048	28.940	48.191	1.00	0.00	H
ATOM	630	N1	DA	20	42.546	30.070	49.796	1.00	0.00	N
ATOM	631	C2	DA	20	43.070	31.016	50.599	1.00	0.00	C
ATOM	632	H2	DA	20	44.112	30.816	50.796	1.00	0.00	H
ATOM	633	N3	DA	20	42.426	32.102	51.114	1.00	0.00	N
ATOM	634	C4	DA	20	41.145	32.179	50.580	1.00	0.00	C
ATOM	635	C3'	DA	20	39.736	36.317	52.635	1.00	0.00	C
ATOM	636	H3'	DA	20	38.894	36.992	52.470	1.00	0.00	H
ATOM	637	C2'	DA	20	40.172	35.594	51.405	1.00	0.00	C
ATOM	638	H2'	DA	20	39.391	35.561	50.642	1.00	0.00	H
ATOM	639	H2''	DA	20	41.003	36.122	50.934	1.00	0.00	H
ATOM	640	O3'	DA	20	40.835	36.924	53.293	1.00	0.00	O
ATOM	641	P	DT	21	41.416	38.252	52.568	1.00	0.00	P
ATOM	642	OP1	DT	21	41.955	39.220	53.592	1.00	0.00	O
ATOM	643	OP2	DT	21	40.467	38.932	51.620	1.00	0.00	O
ATOM	644	O5'	DT	21	42.694	37.504	51.877	1.00	0.00	O
ATOM	645	C5'	DT	21	43.880	37.216	52.599	1.00	0.00	C
ATOM	646	H5'	DT	21	43.691	36.676	53.527	1.00	0.00	H
ATOM	647	H5''	DT	21	44.291	38.169	52.930	1.00	0.00	H
ATOM	648	C4'	DT	21	44.966	36.494	51.861	1.00	0.00	C
ATOM	649	H4'	DT	21	45.842	36.366	52.501	1.00	0.00	H
ATOM	650	O4'	DT	21	44.631	35.098	51.550	1.00	0.00	O
ATOM	651	C1'	DT	21	45.179	34.702	50.223	1.00	0.00	C
ATOM	652	H1'	DT	21	45.971	33.962	50.360	1.00	0.00	H
ATOM	653	N1	DT	21	44.070	34.236	49.280	1.00	0.00	N
ATOM	654	C6	DT	21	42.930	35.029	48.933	1.00	0.00	C
ATOM	655	H6	DT	21	42.746	35.955	49.457	1.00	0.00	H
ATOM	656	C5	DT	21	42.030	34.579	48.031	1.00	0.00	C

ATOM	657	C7	DT	21	40.798	35.470	47.656	1.00	0.00	C
ATOM	658	H71	DT	21	40.862	35.753	46.604	1.00	0.00	H
ATOM	659	H72	DT	21	39.861	34.922	47.771	1.00	0.00	H
ATOM	660	H73	DT	21	40.719	36.416	48.195	1.00	0.00	H
ATOM	661	C4	DT	21	42.293	33.258	47.325	1.00	0.00	C
ATOM	662	O4	DT	21	41.526	32.588	46.688	1.00	0.00	O
ATOM	663	N3	DT	21	43.504	32.716	47.588	1.00	0.00	N
ATOM	664	H3	DT	21	43.747	31.822	47.182	1.00	0.00	H
ATOM	665	C2	DT	21	44.349	33.129	48.530	1.00	0.00	C
ATOM	666	O2	DT	21	45.405	32.568	48.615	1.00	0.00	O
ATOM	667	C3'	DT	21	45.398	37.112	50.412	1.00	0.00	C
ATOM	668	H3'	DT	21	44.499	37.537	49.960	1.00	0.00	H
ATOM	669	C2'	DT	21	45.923	35.888	49.717	1.00	0.00	C
ATOM	670	H2'	DT	21	45.722	36.109	48.667	1.00	0.00	H
ATOM	671	H2''	DT	21	46.980	35.738	49.950	1.00	0.00	H
ATOM	672	O3'	DT	21	46.461	38.091	50.561	1.00	0.00	O
ATOM	673	P	1WA	22	46.750	38.908	49.259	1.00	0.00	P
ATOM	674	OP1	1WA	22	47.476	40.249	49.610	1.00	0.00	O
ATOM	675	OP2	1WA	22	45.470	39.346	48.605	1.00	0.00	O
ATOM	676	O5'	1WA	22	47.687	37.857	48.522	1.00	0.00	O
ATOM	677	C5'	1WA	22	48.005	38.035	47.195	1.00	0.00	C
ATOM	678	H10	1WA	22	48.859	38.716	47.221	1.00	0.00	H
ATOM	679	H11	1WA	22	47.200	38.543	46.660	1.00	0.00	H
ATOM	680	C4'	1WA	22	48.381	36.652	46.543	1.00	0.00	C
ATOM	681	H9	1WA	22	49.158	36.160	47.131	1.00	0.00	H
ATOM	682	O4'	1WA	22	47.154	35.888	46.487	1.00	0.00	O
ATOM	683	C1'	1WA	22	47.188	34.890	45.462	1.00	0.00	C
ATOM	684	N9	1WA	22	45.830	34.626	45.012	1.00	0.00	N
ATOM	685	C4	1WA	22	45.444	33.470	44.323	1.00	0.00	C
ATOM	686	N3	1WA	22	46.312	32.585	43.676	1.00	0.00	N
ATOM	687	C2	1WA	22	45.575	31.697	42.939	1.00	0.00	C
ATOM	688	N1	1WA	22	44.278	31.656	42.661	1.00	0.00	N
ATOM	689	C6	1WA	22	43.532	32.669	43.300	1.00	0.00	C
ATOM	690	N5	1WA	22	44.064	33.603	44.143	1.00	0.00	N
ATOM	691	C7	1WA	22	43.593	34.768	44.591	1.00	0.00	C
ATOM	692	H1	1WA	22	42.595	35.119	44.343	1.00	0.00	H
ATOM	693	C8	1WA	22	44.687	35.463	45.131	1.00	0.00	C
ATOM	694	H2	1WA	22	44.760	36.480	45.508	1.00	0.00	H
ATOM	695	O6	1WA	22	42.355	32.723	42.963	1.00	0.00	O
ATOM	696	N2	1WA	22	46.219	30.677	42.417	1.00	0.00	N
ATOM	697	H3	1WA	22	45.707	30.086	41.765	1.00	0.00	H
ATOM	698	H4	1WA	22	47.232	30.629	42.333	1.00	0.00	H
ATOM	699	H5	1WA	22	47.722	33.994	45.783	1.00	0.00	H
ATOM	700	C2'	1WA	22	47.984	35.676	44.331	1.00	0.00	C
ATOM	701	H6	1WA	22	47.225	36.111	43.678	1.00	0.00	H
ATOM	702	H7	1WA	22	48.569	35.080	43.626	1.00	0.00	H
ATOM	703	C3'	1WA	22	48.811	36.759	45.078	1.00	0.00	C
ATOM	704	H8	1WA	22	49.848	36.449	44.934	1.00	0.00	H
ATOM	705	O3'	1WA	22	48.536	38.072	44.492	1.00	0.00	O
ATOM	706	P	1WA	23	49.551	38.622	43.362	1.00	0.00	P
ATOM	707	OP1	1WA	23	50.656	39.374	44.123	1.00	0.00	O
ATOM	708	OP2	1WA	23	48.833	39.630	42.526	1.00	0.00	O
ATOM	709	O5'	1WA	23	50.095	37.313	42.584	1.00	0.00	O
ATOM	710	C5'	1WA	23	49.986	37.330	41.133	1.00	0.00	C
ATOM	711	H10	1WA	23	50.993	37.415	40.720	1.00	0.00	H
ATOM	712	H11	1WA	23	49.314	38.084	40.718	1.00	0.00	H
ATOM	713	C4'	1WA	23	49.386	35.984	40.879	1.00	0.00	C
ATOM	714	H9	1WA	23	49.969	35.241	41.427	1.00	0.00	H
ATOM	715	O4'	1WA	23	48.023	35.800	41.292	1.00	0.00	O
ATOM	716	C1'	1WA	23	47.434	34.596	40.748	1.00	0.00	C
ATOM	717	N9	1WA	23	45.956	34.709	40.762	1.00	0.00	N
ATOM	718	C4	1WA	23	45.078	33.779	40.174	1.00	0.00	C
ATOM	719	N3	1WA	23	45.458	32.601	39.557	1.00	0.00	N
ATOM	720	C2	1WA	23	44.379	32.024	38.868	1.00	0.00	C
ATOM	721	N1	1WA	23	43.121	32.479	38.762	1.00	0.00	N
ATOM	722	C6	1WA	23	42.726	33.525	39.583	1.00	0.00	C
ATOM	723	N5	1WA	23	43.738	34.269	40.187	1.00	0.00	N
ATOM	724	C7	1WA	23	43.755	35.443	40.878	1.00	0.00	C
ATOM	725	H1	1WA	23	42.806	35.960	40.993	1.00	0.00	H

ATOM	726	C8	1WA	23	45.148	35.820	41.145	1.00	0.00	C
ATOM	727	H2	1WA	23	45.582	36.699	41.616	1.00	0.00	H
ATOM	728	O6	1WA	23	41.522	33.844	39.623	1.00	0.00	O
ATOM	729	N2	1WA	23	44.674	30.939	38.138	1.00	0.00	N
ATOM	730	H3	1WA	23	45.616	30.620	37.919	1.00	0.00	H
ATOM	731	H4	1WA	23	43.892	30.430	37.731	1.00	0.00	H
ATOM	732	H5	1WA	23	47.809	33.831	41.431	1.00	0.00	H
ATOM	733	C2'	1WA	23	48.131	34.544	39.366	1.00	0.00	C
ATOM	734	H6	1WA	23	48.358	33.518	39.068	1.00	0.00	H
ATOM	735	H7	1WA	23	47.482	34.993	38.611	1.00	0.00	H
ATOM	736	C3'	1WA	23	49.391	35.382	39.466	1.00	0.00	C
ATOM	737	H8	1WA	23	50.273	34.752	39.334	1.00	0.00	H
ATOM	738	O3'	1WA	23	49.412	36.455	38.498	1.00	0.00	O
ATOM	739	P	1WA	24	50.657	36.807	37.467	1.00	0.00	P
ATOM	740	OP1	1WA	24	51.984	36.586	38.100	1.00	0.00	O
ATOM	741	OP2	1WA	24	50.692	38.237	37.011	1.00	0.00	O
ATOM	742	O5'	1WA	24	50.463	35.720	36.283	1.00	0.00	O
ATOM	743	C5'	1WA	24	49.566	35.982	35.183	1.00	0.00	C
ATOM	744	H10	1WA	24	50.105	35.739	34.266	1.00	0.00	H
ATOM	745	H11	1WA	24	49.328	37.043	35.079	1.00	0.00	H
ATOM	746	C4'	1WA	24	48.283	35.123	35.276	1.00	0.00	C
ATOM	747	H9	1WA	24	48.510	34.171	35.760	1.00	0.00	H
ATOM	748	O4'	1WA	24	47.248	35.668	36.094	1.00	0.00	O
ATOM	749	C1'	1WA	24	46.103	34.792	35.814	1.00	0.00	C
ATOM	750	N9	1WA	24	44.819	35.240	36.480	1.00	0.00	N
ATOM	751	C4	1WA	24	43.501	34.898	36.168	1.00	0.00	C
ATOM	752	N3	1WA	24	43.080	33.924	35.285	1.00	0.00	N
ATOM	753	C2	1WA	24	41.681	33.876	35.261	1.00	0.00	C
ATOM	754	N1	1WA	24	40.772	34.562	35.883	1.00	0.00	N
ATOM	755	C6	1WA	24	41.258	35.512	36.688	1.00	0.00	C
ATOM	756	N5	1WA	24	42.617	35.725	36.895	1.00	0.00	N
ATOM	757	C7	1WA	24	43.360	36.457	37.766	1.00	0.00	C
ATOM	758	H1	1WA	24	42.972	37.271	38.374	1.00	0.00	H
ATOM	759	C8	1WA	24	44.693	36.196	37.539	1.00	0.00	C
ATOM	760	H2	1WA	24	45.569	36.633	38.012	1.00	0.00	H
ATOM	761	O6	1WA	24	40.517	36.302	37.188	1.00	0.00	O
ATOM	762	N2	1WA	24	41.163	32.953	34.487	1.00	0.00	N
ATOM	763	H3	1WA	24	41.704	32.267	33.965	1.00	0.00	H
ATOM	764	H4	1WA	24	40.168	32.741	34.469	1.00	0.00	H
ATOM	765	H5	1WA	24	46.154	33.794	36.253	1.00	0.00	H
ATOM	766	C2'	1WA	24	46.058	34.637	34.302	1.00	0.00	C
ATOM	767	H6	1WA	24	45.433	35.399	33.831	1.00	0.00	H
ATOM	768	H7	1WA	24	45.659	33.652	34.050	1.00	0.00	H
ATOM	769	C3'	1WA	24	47.500	34.877	33.927	1.00	0.00	C
ATOM	770	H8	1WA	24	47.810	33.952	33.436	1.00	0.00	H
ATOM	771	O3'	1WA	24	47.528	35.995	33.071	1.00	0.00	O
ATOM	772	P	1W5	25	48.106	35.805	31.601	1.00	0.00	P
ATOM	773	OP1	1W5	25	49.293	34.884	31.573	1.00	0.00	O
ATOM	774	OP2	1W5	25	48.269	37.139	30.903	1.00	0.00	O
ATOM	775	O5'	1W5	25	46.723	35.058	31.115	1.00	0.00	O
ATOM	776	C5'	1W5	25	45.769	35.858	30.336	1.00	0.00	C
ATOM	777	H7	1W5	25	45.625	36.805	30.861	1.00	0.00	H
ATOM	778	H8	1W5	25	46.192	36.009	29.341	1.00	0.00	H
ATOM	779	C4'	1W5	25	44.402	35.079	30.100	1.00	0.00	C
ATOM	780	H6	1W5	25	44.526	34.149	29.542	1.00	0.00	H
ATOM	781	O4'	1W5	25	43.794	34.771	31.395	1.00	0.00	O
ATOM	782	C1'	1W5	25	42.342	34.771	31.203	1.00	0.00	C
ATOM	783	C1	1W5	25	41.782	35.702	32.249	1.00	0.00	C
ATOM	784	C2	1W5	25	40.348	35.672	32.431	1.00	0.00	C
ATOM	785	O2	1W5	25	39.635	34.831	31.933	1.00	0.00	O
ATOM	786	N3	1W5	25	39.818	36.630	33.234	1.00	0.00	N
ATOM	787	C4	1W5	25	40.544	37.586	33.921	1.00	0.00	C
ATOM	788	C5	1W5	25	41.890	37.599	33.846	1.00	0.00	C
ATOM	789	C6	1W5	25	42.522	36.626	33.006	1.00	0.00	C
ATOM	790	H1	1W5	25	43.606	36.706	32.985	1.00	0.00	H
ATOM	791	N5	1W5	25	42.686	38.540	34.510	1.00	0.00	N
ATOM	792	ON1	1W5	25	43.584	39.001	33.768	1.00	0.00	O
ATOM	793	ON2	1W5	25	42.355	39.042	35.589	1.00	0.00	O
ATOM	794	N4	1W5	25	39.764	38.420	34.656	1.00	0.00	N

ATOM	795	H9	1W5	25	38.775	38.239	34.498	1.00	0.00	H
ATOM	796	H10	1W5	25	40.086	38.872	35.510	1.00	0.00	H
ATOM	797	H11	1W5	25	38.823	36.449	33.239	1.00	0.00	H
ATOM	798	H2	1W5	25	41.955	33.754	31.294	1.00	0.00	H
ATOM	799	C2'	1W5	25	42.066	35.317	29.789	1.00	0.00	C
ATOM	800	H3	1W5	25	41.725	34.443	29.231	1.00	0.00	H
ATOM	801	H4	1W5	25	41.243	36.028	29.697	1.00	0.00	H
ATOM	802	C3'	1W5	25	43.436	35.931	29.338	1.00	0.00	C
ATOM	803	H5	1W5	25	43.535	37.008	29.485	1.00	0.00	H
ATOM	804	O3'	1W5	25	43.574	35.693	27.942	1.00	0.00	O
ATOM	805	P	1W5	26	42.672	36.523	26.912	1.00	0.00	P
ATOM	806	OP1	1W5	26	43.066	36.422	25.465	1.00	0.00	O
ATOM	807	OP2	1W5	26	42.614	37.985	27.055	1.00	0.00	O
ATOM	808	O5'	1W5	26	41.311	35.688	27.043	1.00	0.00	O
ATOM	809	C5'	1W5	26	40.224	36.203	26.255	1.00	0.00	C
ATOM	810	H7	1W5	26	40.182	37.278	26.443	1.00	0.00	H
ATOM	811	H8	1W5	26	40.458	36.014	25.205	1.00	0.00	H
ATOM	812	C4'	1W5	26	38.849	35.475	26.514	1.00	0.00	C
ATOM	813	H6	1W5	26	38.997	34.394	26.577	1.00	0.00	H
ATOM	814	O4'	1W5	26	38.299	35.893	27.742	1.00	0.00	O
ATOM	815	C1'	1W5	26	36.872	36.130	27.531	1.00	0.00	C
ATOM	816	C1	1W5	26	36.242	37.127	28.567	1.00	0.00	C
ATOM	817	C2	1W5	26	34.841	37.111	28.742	1.00	0.00	C
ATOM	818	O2	1W5	26	34.118	36.332	28.114	1.00	0.00	O
ATOM	819	N3	1W5	26	34.362	38.012	29.567	1.00	0.00	N
ATOM	820	C4	1W5	26	35.119	38.828	30.316	1.00	0.00	C
ATOM	821	C5	1W5	26	36.499	38.893	30.244	1.00	0.00	C
ATOM	822	C6	1W5	26	37.028	37.953	29.345	1.00	0.00	C
ATOM	823	H1	1W5	26	38.108	37.972	29.221	1.00	0.00	H
ATOM	824	N5	1W5	26	37.382	39.696	30.990	1.00	0.00	N
ATOM	825	ON1	1W5	26	38.032	39.130	31.778	1.00	0.00	O
ATOM	826	ON2	1W5	26	37.061	40.879	31.058	1.00	0.00	O
ATOM	827	N4	1W5	26	34.399	39.660	31.027	1.00	0.00	N
ATOM	828	H9	1W5	26	33.397	39.606	30.858	1.00	0.00	H
ATOM	829	H10	1W5	26	34.731	40.408	31.632	1.00	0.00	H
ATOM	830	H11	1W5	26	33.369	37.957	29.753	1.00	0.00	H
ATOM	831	H2	1W5	26	36.335	35.187	27.655	1.00	0.00	H
ATOM	832	C2'	1W5	26	36.766	36.638	26.079	1.00	0.00	C
ATOM	833	H3	1W5	26	35.739	36.462	25.749	1.00	0.00	H
ATOM	834	H4	1W5	26	37.092	37.679	26.094	1.00	0.00	H
ATOM	835	C3'	1W5	26	37.841	35.793	25.390	1.00	0.00	C
ATOM	836	H5	1W5	26	38.229	36.273	24.489	1.00	0.00	H
ATOM	837	O3'	1W5	26	37.282	34.510	24.959	1.00	0.00	O
ATOM	838	P	1W5	27	36.496	34.266	23.529	1.00	0.00	P
ATOM	839	OP1	1W5	27	36.360	32.802	23.317	1.00	0.00	O
ATOM	840	OP2	1W5	27	37.307	34.788	22.363	1.00	0.00	O
ATOM	841	O5'	1W5	27	35.055	34.764	23.894	1.00	0.00	O
ATOM	842	C5'	1W5	27	34.390	35.787	23.032	1.00	0.00	C
ATOM	843	H7	1W5	27	35.007	36.685	22.962	1.00	0.00	H
ATOM	844	H8	1W5	27	34.282	35.437	22.004	1.00	0.00	H
ATOM	845	C4'	1W5	27	33.017	36.154	23.503	1.00	0.00	C
ATOM	846	H6	1W5	27	32.478	35.226	23.701	1.00	0.00	H
ATOM	847	O4'	1W5	27	33.021	36.898	24.716	1.00	0.00	O
ATOM	848	C1'	1W5	27	31.815	37.572	24.903	1.00	0.00	C
ATOM	849	C1	1W5	27	32.131	38.781	25.749	1.00	0.00	C
ATOM	850	C2	1W5	27	31.016	39.408	26.313	1.00	0.00	C
ATOM	851	O2	1W5	27	29.874	39.131	25.999	1.00	0.00	O
ATOM	852	N3	1W5	27	31.272	40.338	27.268	1.00	0.00	N
ATOM	853	C4	1W5	27	32.545	40.789	27.568	1.00	0.00	C
ATOM	854	C5	1W5	27	33.667	40.348	26.890	1.00	0.00	C
ATOM	855	C6	1W5	27	33.406	39.266	26.025	1.00	0.00	C
ATOM	856	H1	1W5	27	34.240	38.772	25.532	1.00	0.00	H
ATOM	857	N5	1W5	27	34.998	40.809	27.081	1.00	0.00	N
ATOM	858	ON1	1W5	27	35.254	40.968	28.230	1.00	0.00	O
ATOM	859	ON2	1W5	27	35.760	40.801	26.087	1.00	0.00	O
ATOM	860	N4	1W5	27	32.526	41.820	28.420	1.00	0.00	N
ATOM	861	H9	1W5	27	33.322	42.050	29.011	1.00	0.00	H
ATOM	862	H10	1W5	27	31.705	42.383	28.632	1.00	0.00	H
ATOM	863	H11	1W5	27	30.474	40.729	27.749	1.00	0.00	H

ATOM	864	H2	1W5	27	31.041	36.950	25.357	1.00	0.00	H
ATOM	865	C2'	1W5	27	31.438	37.977	23.470	1.00	0.00	C
ATOM	866	H3	1W5	27	30.359	37.871	23.333	1.00	0.00	H
ATOM	867	H4	1W5	27	31.748	38.998	23.235	1.00	0.00	H
ATOM	868	C3'	1W5	27	32.233	36.978	22.543	1.00	0.00	C
ATOM	869	H5	1W5	27	32.969	37.508	21.935	1.00	0.00	H
ATOM	870	O3'	1W5	27	31.288	36.268	21.705	1.00	0.00	O
ATOM	871	P	DA	28	30.700	36.860	20.363	1.00	0.00	P
ATOM	872	OP1	DA	28	30.282	35.830	19.378	1.00	0.00	O
ATOM	873	OP2	DA	28	31.742	37.533	19.564	1.00	0.00	O
ATOM	874	O5'	DA	28	29.504	37.928	20.871	1.00	0.00	O
ATOM	875	C5'	DA	28	28.129	37.415	20.929	1.00	0.00	C
ATOM	876	H5'	DA	28	28.075	36.963	21.919	1.00	0.00	H
ATOM	877	H5''	DA	28	27.954	36.626	20.198	1.00	0.00	H
ATOM	878	C4'	DA	28	26.982	38.393	20.704	1.00	0.00	C
ATOM	879	H4'	DA	28	26.027	37.909	20.919	1.00	0.00	H
ATOM	880	O4'	DA	28	27.079	39.443	21.649	1.00	0.00	O
ATOM	881	C1'	DA	28	27.464	40.735	21.129	1.00	0.00	C
ATOM	882	H1'	DA	28	26.576	41.350	20.967	1.00	0.00	H
ATOM	883	N9	DA	28	28.421	41.191	22.169	1.00	0.00	N
ATOM	884	C8	DA	28	29.779	41.022	22.427	1.00	0.00	C
ATOM	885	H8	DA	28	30.296	40.350	21.759	1.00	0.00	H
ATOM	886	N7	DA	28	30.240	41.674	23.408	1.00	0.00	N
ATOM	887	C5	DA	28	29.187	42.471	23.761	1.00	0.00	C
ATOM	888	C6	DA	28	29.000	43.586	24.572	1.00	0.00	C
ATOM	889	N6	DA	28	30.022	44.214	25.270	1.00	0.00	N
ATOM	890	H61	DA	28	29.942	45.124	25.718	1.00	0.00	H
ATOM	891	H62	DA	28	30.940	43.810	25.445	1.00	0.00	H
ATOM	892	N1	DA	28	27.868	44.341	24.652	1.00	0.00	N
ATOM	893	C2	DA	28	26.842	43.853	23.933	1.00	0.00	C
ATOM	894	H2	DA	28	25.979	44.503	23.908	1.00	0.00	H
ATOM	895	N3	DA	28	26.803	42.772	23.070	1.00	0.00	N
ATOM	896	C4	DA	28	28.066	42.220	23.021	1.00	0.00	C
ATOM	897	C3'	DA	28	27.249	39.040	19.384	1.00	0.00	C
ATOM	898	H3'	DA	28	27.924	38.401	18.810	1.00	0.00	H
ATOM	899	C2'	DA	28	28.034	40.360	19.726	1.00	0.00	C
ATOM	900	H2'	DA	28	29.116	40.216	19.702	1.00	0.00	H
ATOM	901	H2''	DA	28	27.941	41.173	19.003	1.00	0.00	H
ATOM	902	O3'	DA	28	25.952	39.254	18.756	1.00	0.00	O
ATOM	903	P	DT	29	25.988	39.868	17.204	1.00	0.00	P
ATOM	904	OP1	DT	29	24.854	39.532	16.358	1.00	0.00	O
ATOM	905	OP2	DT	29	27.267	39.586	16.404	1.00	0.00	O
ATOM	906	O5'	DT	29	25.671	41.407	17.552	1.00	0.00	O
ATOM	907	C5'	DT	29	24.462	41.864	18.054	1.00	0.00	C
ATOM	908	H5'	DT	29	24.113	41.399	18.975	1.00	0.00	H
ATOM	909	H5''	DT	29	23.704	41.916	17.272	1.00	0.00	H
ATOM	910	C4'	DT	29	24.588	43.287	18.564	1.00	0.00	C
ATOM	911	H4'	DT	29	23.650	43.560	19.051	1.00	0.00	H
ATOM	912	O4'	DT	29	25.618	43.495	19.586	1.00	0.00	O
ATOM	913	C1'	DT	29	26.125	44.885	19.467	1.00	0.00	C
ATOM	914	H1'	DT	29	25.374	45.496	19.973	1.00	0.00	H
ATOM	915	N1	DT	29	27.466	45.048	20.053	1.00	0.00	N
ATOM	916	C6	DT	29	28.492	44.245	19.671	1.00	0.00	C
ATOM	917	H6	DT	29	28.314	43.474	18.936	1.00	0.00	H
ATOM	918	C5	DT	29	29.706	44.218	20.309	1.00	0.00	C
ATOM	919	C7	DT	29	30.814	43.349	19.737	1.00	0.00	C
ATOM	920	H71	DT	29	31.218	42.656	20.478	1.00	0.00	H
ATOM	921	H72	DT	29	30.590	42.708	18.881	1.00	0.00	H
ATOM	922	H73	DT	29	31.611	44.011	19.390	1.00	0.00	H
ATOM	923	C4	DT	29	29.962	45.106	21.503	1.00	0.00	C
ATOM	924	O4	DT	29	31.001	45.276	21.972	1.00	0.00	O
ATOM	925	N3	DT	29	28.863	45.787	21.851	1.00	0.00	N
ATOM	926	H3	DT	29	29.051	46.380	22.648	1.00	0.00	H
ATOM	927	C2	DT	29	27.669	45.893	21.204	1.00	0.00	C
ATOM	928	O2	DT	29	26.828	46.574	21.782	1.00	0.00	O
ATOM	929	C3'	DT	29	24.895	44.324	17.376	1.00	0.00	C
ATOM	930	H3'	DT	29	25.404	43.777	16.579	1.00	0.00	H
ATOM	931	C2'	DT	29	25.984	45.183	17.949	1.00	0.00	C
ATOM	932	H2'	DT	29	26.940	45.171	17.421	1.00	0.00	H

ATOM	933	H2''	DT	29	25.648	46.223	17.945	1.00	0.00	H
ATOM	934	O3'	DT	29	23.648	44.989	17.099	1.00	0.00	O
ATOM	935	P	DA	30	23.702	46.163	15.913	1.00	0.00	P
ATOM	936	OP1	DA	30	22.405	46.170	15.191	1.00	0.00	O
ATOM	937	OP2	DA	30	24.792	45.979	14.948	1.00	0.00	O
ATOM	938	O5'	DA	30	23.999	47.559	16.792	1.00	0.00	O
ATOM	939	C5'	DA	30	23.224	47.967	17.898	1.00	0.00	C
ATOM	940	H5'	DA	30	23.032	47.112	18.546	1.00	0.00	H
ATOM	941	H5''	DA	30	22.229	48.262	17.565	1.00	0.00	H
ATOM	942	C4'	DA	30	23.919	49.071	18.770	1.00	0.00	C
ATOM	943	H4'	DA	30	23.231	49.294	19.588	1.00	0.00	H
ATOM	944	O4'	DA	30	25.087	48.465	19.357	1.00	0.00	O
ATOM	945	C1'	DA	30	26.055	49.582	19.533	1.00	0.00	C
ATOM	946	H1'	DA	30	25.739	50.302	20.292	1.00	0.00	H
ATOM	947	N9	DA	30	27.403	49.077	19.725	1.00	0.00	N
ATOM	948	C8	DA	30	28.223	48.175	18.965	1.00	0.00	C
ATOM	949	H8	DA	30	27.775	47.680	18.115	1.00	0.00	H
ATOM	950	N7	DA	30	29.419	48.054	19.408	1.00	0.00	N
ATOM	951	C5	DA	30	29.415	48.878	20.550	1.00	0.00	C
ATOM	952	C6	DA	30	30.361	49.216	21.524	1.00	0.00	C
ATOM	953	N6	DA	30	31.641	48.780	21.525	1.00	0.00	N
ATOM	954	H61	DA	30	32.314	49.009	22.253	1.00	0.00	H
ATOM	955	H62	DA	30	32.060	48.178	20.820	1.00	0.00	H
ATOM	956	N1	DA	30	30.134	50.096	22.517	1.00	0.00	N
ATOM	957	C2	DA	30	28.868	50.693	22.583	1.00	0.00	C
ATOM	958	H2	DA	30	28.713	51.295	23.466	1.00	0.00	H
ATOM	959	N3	DA	30	27.809	50.375	21.823	1.00	0.00	N
ATOM	960	C4	DA	30	28.214	49.474	20.777	1.00	0.00	C
ATOM	961	C3'	DA	30	24.491	50.264	17.983	1.00	0.00	C
ATOM	962	H3'	DA	30	24.345	50.078	16.917	1.00	0.00	H
ATOM	963	C2'	DA	30	25.979	50.411	18.259	1.00	0.00	C
ATOM	964	H2'	DA	30	26.523	49.898	17.463	1.00	0.00	H
ATOM	965	H2''	DA	30	26.303	51.448	18.369	1.00	0.00	H
ATOM	966	O3'	DA	30	23.864	51.490	18.320	1.00	0.00	O
ATOM	967	P	DA	31	24.165	52.831	17.412	1.00	0.00	P
ATOM	968	OP1	DA	31	22.886	53.515	16.973	1.00	0.00	O
ATOM	969	OP2	DA	31	24.907	52.418	16.173	1.00	0.00	O
ATOM	970	O5'	DA	31	25.171	53.621	18.465	1.00	0.00	O
ATOM	971	C5'	DA	31	24.648	54.203	19.634	1.00	0.00	C
ATOM	972	H5'	DA	31	24.192	53.444	20.270	1.00	0.00	H
ATOM	973	H5''	DA	31	23.917	54.994	19.468	1.00	0.00	H
ATOM	974	C4'	DA	31	25.777	54.871	20.351	1.00	0.00	C
ATOM	975	H4'	DA	31	25.374	55.297	21.272	1.00	0.00	H
ATOM	976	O4'	DA	31	26.777	53.955	20.825	1.00	0.00	O
ATOM	977	C1'	DA	31	28.086	54.613	20.734	1.00	0.00	C
ATOM	978	H1'	DA	31	28.419	55.261	21.547	1.00	0.00	H
ATOM	979	N9	DA	31	29.116	53.605	20.387	1.00	0.00	N
ATOM	980	C8	DA	31	29.050	52.576	19.484	1.00	0.00	C
ATOM	981	H8	DA	31	28.129	52.273	19.009	1.00	0.00	H
ATOM	982	N7	DA	31	30.190	51.996	19.332	1.00	0.00	N
ATOM	983	C5	DA	31	30.992	52.504	20.336	1.00	0.00	C
ATOM	984	C6	DA	31	32.305	52.298	20.799	1.00	0.00	C
ATOM	985	N6	DA	31	33.115	51.330	20.489	1.00	0.00	N
ATOM	986	H61	DA	31	34.061	51.167	20.827	1.00	0.00	H
ATOM	987	H62	DA	31	32.805	50.542	19.925	1.00	0.00	H
ATOM	988	N1	DA	31	32.855	53.083	21.663	1.00	0.00	N
ATOM	989	C2	DA	31	32.181	54.157	22.186	1.00	0.00	C
ATOM	990	H2	DA	31	32.746	54.693	22.935	1.00	0.00	H
ATOM	991	N3	DA	31	30.851	54.485	21.919	1.00	0.00	N
ATOM	992	C4	DA	31	30.383	53.581	20.946	1.00	0.00	C
ATOM	993	C3'	DA	31	26.428	55.892	19.503	1.00	0.00	C
ATOM	994	H3'	DA	31	26.086	55.989	18.470	1.00	0.00	H
ATOM	995	C2'	DA	31	27.950	55.602	19.596	1.00	0.00	C
ATOM	996	H2'	DA	31	28.325	55.222	18.643	1.00	0.00	H
ATOM	997	H2''	DA	31	28.522	56.501	19.838	1.00	0.00	H
ATOM	998	O3'	DA	31	26.143	57.202	20.187	1.00	0.00	O
ATOM	999	P	DG3	32	26.408	58.585	19.419	1.00	0.00	P
ATOM	1000	OP1	DG3	32	25.526	59.715	19.881	1.00	0.00	O
ATOM	1001	OP2	DG3	32	26.136	58.601	17.965	1.00	0.00	O

```
ATOM   1002  O5' DG3    32      27.984  59.036  19.789  1.00 0.00           O
ATOM   1003  C5' DG3    32      28.461  59.047  21.118  1.00 0.00           C
ATOM   1004  H5' DG3    32      28.210  58.139  21.667  1.00 0.00           H
ATOM   1005 H5'' DG3    32      28.093  59.925  21.649  1.00 0.00           H
ATOM   1006  C4' DG3    32      29.977  58.972  21.112  1.00 0.00           C
ATOM   1007  H4' DG3    32      30.329  59.153  22.130  1.00 0.00           H
ATOM   1008  O4' DG3    32      30.517  57.676  20.639  1.00 0.00           O
ATOM   1009  C1' DG3    32      31.683  57.906  19.895  1.00 0.00           C
ATOM   1010  H1' DG3    32      32.486  58.154  20.591  1.00 0.00           H
ATOM   1011  N9  DG3    32      31.960  56.670  19.270  1.00 0.00           N
ATOM   1012  C8  DG3    32      31.256  56.069  18.301  1.00 0.00           C
ATOM   1013  H8  DG3    32      30.364  56.525  17.897  1.00 0.00           H
ATOM   1014  N7  DG3    32      31.867  54.968  17.876  1.00 0.00           N
ATOM   1015  C5  DG3    32      33.079  54.926  18.602  1.00 0.00           C
ATOM   1016  C6  DG3    32      34.173  53.949  18.672  1.00 0.00           C
ATOM   1017  O6  DG3    32      34.379  52.923  18.060  1.00 0.00           O
ATOM   1018  N1  DG3    32      35.189  54.375  19.467  1.00 0.00           N
ATOM   1019  H1  DG3    32      35.961  53.739  19.614  1.00 0.00           H
ATOM   1020  C2  DG3    32      35.129  55.483  20.291  1.00 0.00           C
ATOM   1021  N2  DG3    32      36.052  55.615  21.227  1.00 0.00           N
ATOM   1022  H21 DG3    32      36.911  55.075  21.304  1.00 0.00           H
ATOM   1023  H22 DG3    32      35.947  56.358  21.915  1.00 0.00           H
ATOM   1024  N3  DG3    32      34.163  56.351  20.279  1.00 0.00           N
ATOM   1025  C4  DG3    32      33.165  56.017  19.401  1.00 0.00           C
ATOM   1026  C3' DG3    32      30.632  60.030  20.164  1.00 0.00           C
ATOM   1027  H3' DG3    32      29.900  60.670  19.668  1.00 0.00           H
ATOM   1028  C2' DG3    32      31.371  59.198  19.122  1.00 0.00           C
ATOM   1029  H2' DG3    32      30.656  59.000  18.321  1.00 0.00           H
ATOM   1030 H2'' DG3    32      32.280  59.682  18.760  1.00 0.00           H
ATOM   1031  O3' DG3    32      31.581  60.917  20.835  1.00 0.00           O
ATOM   1032 HO3' DG3    32      31.124  61.267  21.620  1.00 0.00           H
END
```
